# Evolution for enhanced extracellular electron transfer in *Geobacter sulfurreducens* over seventeen years of continuous current generation

**DOI:** 10.3389/fmicb.2026.1771963

**Published:** 2026-05-08

**Authors:** Dawn E. Holmes, Trevor L. Woodard, Kelly P. Nevin, Madeline Vargas, Katherine Romoser, Sydney E. Holmes, Derek R. Lovley

**Affiliations:** 1 Department of Microbiology, University of Massachusetts Amherst, Morrill IVN Science Center, Amherst, MA, United States; 2 Department of Physical and Biological Sciences, Western New England University, Springfield, MA, United States; 3 Department of Biology, College of the Holy Cross, Worcester, MA, United States

**Keywords:** adaptive evolution, bioelectrochemical systems, conductive biofilms, cytochromes, electrogens, extracellular electron transfer, *Geobacter*, pili

## Abstract

Bioelectrochemical systems that harvest electricity from wastes, soils, or sediments are designed to operate over long periods of time. However, little is known about the long-term adaptation of electroactive microbes to such non-natural environments. *Geobacter sulfurreducens* strain KN400 produces the highest current densities and columbic efficiencies of described pure culture electroactive microorganisms. To investigate long-term adaptation to current production, strain KN400 was grown continuously for 17 years on graphite anodes poised at −400 mV. Isolates recovered from the 17-year-old anode biofilms (designated LT strains) exhibited markedly enhanced extracellular electron transfer capacity, growing ~1.8-fold faster than the parental KN400 strain on Fe(III)-oxide. Crystal violet assays revealed increased surface-associated biofilm biomass on glass by the adapted strains. Quantitative transcriptomics revealed strong upregulation of *pilA*, *omcZ*, and *omcB* during Fe(III) oxide reduction, consistent with enhanced extracellular electron transfer capability. Comparative genomic analysis demonstrated genome reduction relative to KN400 and identified numerous mutations in genes for *c*-type cytochromes, signal transduction proteins, and transcriptional regulators, including modifications in *c-di-GMP* riboswitches and diguanylate cyclases that likely promoted expression of extracellular electron transport genes. Genes dispensable for growth on electrodes, including those associated with motility or hydrogen utilization, accumulated mutations consistent with relaxed selective pressure and genetic drift. These results demonstrate that prolonged electrode cultivation can drive genome reduction and regulatory remodeling that optimize electroactive microbes for efficient, biofilm-based electron transfer and may help stabilize long-term bioelectronic systems.

## Introduction

Current-producing bioelectrochemical systems are envisioned as a sustainable source of electricity derived from waste organic matter (Logan et al., 2019; Jalili et al., 2024). In practical applications these “microbial fuel cells” would continuously and stably generate power for years. In contrast, most laboratory studies of current-producing microbes are relatively short-term. This is especially true for studies with pure cultures designed to understand the mechanisms for extracellular electron transfer (EET) to current-harvesting electrodes, which typically last only a few days (Bond and Lovley, 2003; Chaudhuri and Lovley, 2003; Rabaey et al., 2005; Ringeisen et al., 2006).

Bioelectrochemical systems are man-made, and EET to electrodes has some differences from natural forms of EET, such as electron transfer to Fe(III) oxides or other microbial species. For example, the electrode provides a large, relatively flat, contiguous conductive surface that can continuously accept electrons. This contrasts with Fe(III) oxides which are typically smaller than the cell, dispersed throughout the environment, and difficult for the cell to directly contact (Lovley and Holmes, 2022; Lovley, 2026). Once Fe(III) in one location is reduced, microbes that rely on direct contact for EET must hunt for more (Childers et al., 2002). The electron-accepting partners for direct interspecies electron transfer may be more analogous to electrodes (Rotaru et al., 2015), but restrictions on the two syntrophic partners synchronizing growth and metabolic rates and the limitations of electron donors in the natural environment limit the rate and extent of electron transfer. Thus, optimal solutions to EET that have evolved for the reduction of poorly soluble minerals and electron transfer to syntrophic partners, presumably over millions of years (Lovley, 2004, 2026), might not necessarily be an optimal strategy for electron transfer to electrodes. Therefore, understanding how microbes might change to optimize EET in bioelectrochemical systems during long-term operation is relevant for the design and operation of these systems.

*Geobacter* species are among the pure cultures that produce the highest current densities in bioelectrochemical systems and are often the predominant current-producing microbes recovered in open bioelectrochemical systems in which a wide diversity of microbes must compete for space within current-producing biofilms (Lovley, 2006; Logan et al., 2019; Jalili et al., 2024). Studies with *Geobacter sulfurreducens* have led to a model for its generation of high currents (Lovley, 2011; Lovley, 2012; Lovley and Holmes, 2022). Electrons released from the oxidation of acetate or other electron donors are passed across the outer-membrane through porin-cytochrome conduits. Which porin-cytochrome conduit has been found to be important in current production depends upon culture conditions (Nevin et al., 2009; Otero et al., 2018). The outer-surface-facing cytochromes of the porin-cytochrome conduits may be able to establish contacts with electrodes to generate some current (Lovley, 2012). However, achieving high current densities requires long-range EET, which enables cells distant from the anode surface to contribute electrons that are transported thorough electrically conductive *G. sulfurreducens* biofilms to the anode (Lovley, 2012; Malvankar et al., 2012a).

There has been considerable debate about the mechanisms for long-range electron transfer through *G. sulfurreducens* biofilms. Electrochemical analyses have consistently detected responses that have been attributed to long-range EET through networks of *c*-type cytochromes (Bond et al., 2012). However, such analyses provide only an indirect assessment of electron transport, and interpretation of the data relies on models developed for highly defined, simple systems in which electron carriers are uniformly distributed throughout the matrix, conditions not found in heterogenous biofilms, where the majority of cytochromes are retained within cells. It has been proposed that *G. sulfurreducens* cytochromes that assemble into filaments (OmcS, OmcZ, OmcE) are responsible for long-range EET in current-producing biofilms (Tabari and Hochbaum, 2025), but experimental evidence refutes this claim (Lovley, 2025). For example, inhibiting cytochrome-based electron transfer with acetyl methionine, had no impact on biofilm conductivity (Malvankar et al., 2012b). Deleting the genes for the filament-forming cytochromes OmcS and OmcE did not inhibit current production and biofilm conductivities were higher than in the parental strain (Malvankar et al., 2012a,b).

Current production was diminished when the gene for OmcZ was deleted (Nevin et al., 2009). However, OmcZ is specifically localized at the biofilm/anode interface in high current-producing systems, eliminating the possibility that OmcZ filaments could account for substantial electron transport through the bulk of current-producing biofilms (Inoue et al., 2010). There was a lack of correlation between OmcZ abundance and biofilm conductivity, further suggesting that OmcZ did not account for the conductivity throughout the biofilm (Malvankar et al., 2012b). The requirement for OmcZ for high current production and its localization suggests that it plays an important role in electron transfer from biofilms to electrodes (Lovley, 2011; Lovley, 2012; Lovley and Holmes, 2022). None of the filament-forming cytochromes are required for Fe(III) oxide reduction (Schwarz et al., 2024).

In contrast to the lack of evidence for cytochromes being the primary agents for long-range electron transfer through current-producing biofilms or for Fe(III) oxide reduction, there is substantial functional evidence that electrically conductive pili (e-pili) play a key role in long-range EET. e-Pili are the most abundant electrically conductive filament emanating from cells (Liu et al., 2021; Schwarz et al., 2024). The conductivity of the pili can be genetically tailored by modifying the abundance of aromatic amino acids encoded in the gene for the pilin monomer PilA (Adhikari et al., 2016; Tan et al., 2017). Expressing pili with low conductivity inhibits Fe(III) oxide reduction and greatly reduces biofilm conductivity and current production, even though outer surface cytochromes continue to be properly displayed (Vargas et al., 2013; Liu et al., 2014; Steidl et al., 2016; Liu et al., 2022). There is a positive correlation between pilin abundance and biofilm conductivity (Malvankar et al., 2011).

Thus, the model for *G. sulfurreducens* production of high current densities derived from gene expression and functional genetic studies in relatively short-term (<1 month) incubations suggests that electron transfer across the outer membrane is primarily through porin-cytochrome conduits, with e-pili functioning as the primary conduit for long-range electron transport through the biofilm and OmcZ facilitating electron transfer to the anode surface.

After 5 months of operation of a current-producing bioelectrochemical system with an anode poised at −400 mV (standard hydrogen electrode) that was inoculated with the type strain of *G. sulfurreducens* (strain PCA), all of the isolates recovered from the anode were a different strain, designated strain KN400, that was significantly better at EET than strain PCA (Yi et al., 2009; Ueki et al., 2012). Genome sequencing (Butler et al., 2012) and quantification of strain KN400-specific genes in cultures (Shrestha et al., 2013) revealed that strain KN400 was not a product of adaptive evolution during growth on the anode, but rather a rare variant contaminant within the original PCA culture, which was comprised of ca. one cell of strain KN400 for every 10^5^ PCA cells (Shrestha et al., 2013). Strain KN400 grew much better than strain PCA on the negatively poised anodes, accounting for its selection in the bioelectrochemical system. Key physiological characteristics accounting for strain KN400’s greater EET capabilities include higher e-pili expression (Yi et al., 2009; Malvankar et al., 2011), lower expression of outer-surface cytochromes (Yi et al., 2009; Liu et al., 2012), an enhanced capacity to adhere to surfaces despite expressing less exopolysaccharide (Yi et al., 2009), and higher biofilm conductivity (Malvankar et al., 2012a). There was no evidence of novel gene acquisition to improve EET (Butler et al., 2012). However, there was a substantial accumulation of mutations in genes for the outer-surface *c*-type cytochromes OmcS and OmcE suggesting likely changes to cytochrome folding, localization, and redox potentials that could impact their effectiveness in EET. In contrast, genes for e-pili synthesis and expression, including the pilin monomer PilA, were highly conserved (Butler et al., 2012). The gene sequences for the outward-facing porin-cytochrome conduit cytochrome OmcB and the outer-surface cytochrome OmcZ, which other than PilA were the two EET-related genes with the greatest increase in gene expression in current-producing biofilms versus biofilms reducing fumarate (Nevin et al., 2009), were the same in KN400 as in strain PCA (Butler et al., 2012).

In order to further evaluate the impact of long-term operation, the biofilm that yielded strain KN400 was continuously operated for an additional 17 years. The results demonstrate that strains evolved from strain KN400 exhibit genome streamlining and modifications that increase the expression of the OmcB, PilA, and OmcZ components previously identified as contributing to high current density production, resulting in a further increase in EET capacity.

## Materials and methods

### Growth and selection conditions

*Geobacter sulfurreducens* strain PCA^T^ (Caccavo et al., 1994) and *G. sulfurreducens* KN400 (Yi et al., 2009) were routinely cultured at 30 °C under strict anaerobic conditions (80:20 N_2_/CO_2_) on NB medium with acetate (15 mM) provided as the electron donor and fumarate (40 mM) as the electron acceptor as previously described (Coppi et al., 2001). The composition of NB medium per liter was 0.42 g KH_2_PO_4_, 0.22 g K_2_HPO_4_, 0.2 g NH_4_Cl, 0.38 g KCl, 0.36 g NaCl, 0.04 g CaCl_2_·2H_2_O, 0.1 g MgSO_4_·7H_2_O, 1.8 g NaHCO_3_, 0.5 g Na_2_CO_3_, 2.04 g NaC_2_H_3_O_2_·3H_2_O, 6.4 g Na_2_C_4_H_4_O_4_, 1.0 mL 100 mM Na_2_SeO_4_, 10 mL DL vitamin (Lovley et al., 1984) and 10 mL NB trace mineral solution. Per liter, the NB trace mineral solution contained 21.4 g nitriloacetic acid, 0.1 g MnCl_2_·4H_2_O, 0.3 g FeSO_4_·7H_2_O, 0.17 g CoCl_2_·6H_2_O, 0.2 g ZnSO_4_·7H_2_O, 0.3 g CuCl_2_·2H_2_O, 0.005 g AlK(SO_4_)_2_·12H_2_O, 0.005 g H_3_BO_3_, 0.09 g Na_2_MoO_4_, 0.11 g NiSO_4_·6H_2_O, and 0.2 g Na_2_WO_4_·2H_2_O. Cysteine (1 mM) was used as a reductant.

To select for strains that were adapted to growth on a current-harvesting electrode, cells were grown anaerobically in continuous flow two-chambered bioelectrochemical systems, as previously described (Reguera et al., 2006; Nevin et al., 2009). Acetate was the sole electron donor and a graphite electrode (65 cm^2^ solid graphite block, 1 in. by 0.5 in. by 3 in., grade G10, Graphite Engineering and Sales, Greenville, MI) poised at −400 mV (versus Ag/AgCl) with a potentiostat served as the sole electron acceptor. Acetate (10 mM) was the electron donor in freshwater medium (2.5 g/L NaHCO3, 0.25 g/L NH4Cl, 0.06 g/L NaHPO4·H_2_O, 0.1 g/L KCl, vitamins and minerals) (Lovley and Phillips, 1988). Once current production reached a maximum, fresh medium (Freshwater medium) was continuously provided to the anode chamber at a flow rate of 30 mL/h as previously described (Reguera et al., 2006). Fresh medium was continuously provided to the flow-through system for a 17 year period. Current measurements were collected from potentiostat outputs every second with a Power Lab 4SP connected to a Macintosh computer, and data was logged with Chart 5.0 software (ADI instruments, Mountain View, CA).

After 17 years of growth on a current harvesting anode poised at −400 mV, a portion of the biofilm was aseptically scraped off the anode surface into freshwater medium with acetate (15 mM) as the donor and Fe(III) oxide (100 mM) as the electron acceptor (Supplementary Figure S1). Serial dilutions were done in the Fe(III)-oxide-acetate medium and cells that grew in the 10^−5^ dilution were streaked onto NB-acetate-fumarate medium solidified with agar (16 g/L). Isolated colonies were then picked and transferred into freshwater Fe(III) oxide-acetate medium and serially diluted to 10^−5^ to ensure that a pure culture was obtained. Culture purity was confirmed with microscopy and by sequencing the 16S rRNA gene.

Once isolates were obtained, they were grown in freshwater medium with acetate (15 mM) provided as the electron donor and fumarate (40 mM), Fe(III) citrate (60 mM), or Fe(III)-oxide (100 mM) as an electron acceptor. Cysteine (1 mM) was used as a reductant for growth with fumarate, whereas Fe(II)-chloride (1.3 mM) was used as the reductant in Fe(III)-citrate and Fe(III)-oxide media.

### DNA extraction and genome sequencing

DNA was extracted from LT (long term) cultures (LT1, LT2, LT3, LT4, LT5) with the MasterPure Complete DNA Purification Kit (Biosearch Technologies). Genomes were sequenced by MR DNA (Molecular Research LP, info@mrdnalab.com) on an Illumina MiSeq platform, producing paired-end reads. Sequences were submitted to the Sequence Read Archive (SRA) and are available under Bioproject ID PRJNA1391578 and accession numbers SAMN4225968- SAMN4225972.

### Sequence analysis

FASTQ read quality was evaluated using FASTQC. Genomes from *G. sulfurreducens* PCA (GCA_000007985) and *G. sulfurreducens* KN400 (GCA_000210155; SRA: ERX311328) were downloaded from Genbank. Snippy (Seeman, 2015) was used to identify polymorphisms in pairwise genome comparisons between PCA and KN400, and between KN400 and LT strains. Protein secondary structure predictions were made with PSIPRED (Jones, 1999) and DSSP (Dictionary of Secondary Structure of Proteins) (Hekkelman et al., 2025) and tertiary predictions were made with AlphaFold (Jumper et al., 2021). All tertiary structures were visualized and compared with the MatchMaker tool in UCSF ChimeraX version 1.10.1 (Goddard et al., 2018; Pettersen et al., 2021), and TM-score (Zhang and Skolnick, 2004). All statistical analyses (paired *t*-tests, Wilcoxon signed-rank tests, independent *t*-tests, χ^2^ tests, McNemar’s tests) were performed in Rstudio version 1.4.1106 using the base stats package (R Core Team, 2021).

### RNA extraction and quantitative RT-PCR

Quantitative RT-PCR was conducted with mRNA extracted from triplicate cultures of the LT1, LT2, LT3, LT4, LT5, KN400 and PCA strains of *G. sulfurreducens* grown in freshwater medium with acetate (15 mM) as electron donor and fumarate (40 mM), Fe(III) citrate (60 mM), or Fe(III) oxide (100 mM) as the electron acceptor. Cells were harvested at mid-log phase, determined spectrophotometrically by measuring absorbance at 600 nm for fumarate-grown cultures and by monitoring Fe(II) production for cultures grown with Fe(III) citrate or Fe(III) oxide. At the time of harvest, 50 mL of culture was mixed with RNA Protect (Qiagen) in a 1:1 ratio, and cells were pelleted by centrifugation at 3,000 x g for 15 min at 4 °C. After centrifugation, the pellets were frozen in liquid nitrogen and stored at −80 °C until RNA extraction procedures were performed. Total RNA from sample pellets was extracted as previously described (Holmes et al., 2012), cleaned with the RNeasy Mini Kit (Qiagen), and then treated with Turbo DNA-free DNAse (Ambion). Further enrichment of mRNA was done with the MICROBExpress kit (Ambion), according to the manufacturer’s instructions.

In order to ensure that samples were not contaminated with genomic DNA, PCR with primers targeting the 16S rRNA gene was done with RNA that had not been reverse transcribed. Complementary DNA (cDNA) was generated from mRNA using the Invitrogen SuperScript IV First Strand Synthesis System (ThermoFisher Sci).

Primer pairs used for qRT-PCR are provided in Supplementary Table S1. The gene coding for recombinase A, *recA*, was used as the housekeeping gene for transcript standardization. All PCR products were amplified and quantified with Power SYBR green PCR master mix (Applied Biosystems, Foster City, CA) and an ABI 7500 real-time PCR system. Each reaction mixture consisted of forward and reverse primers at a final concentration of 200 nM, 5 ng of cDNA, and 12.5 μL of Power SYBR green PCR master mix (Applied Biosystems). Relative levels of expression of the studied genes were calculated by the 2−^ΔΔ*CT*^ threshold cycle (CT) method.

### Cell attachment assays

Cells from mid-log cultures were inoculated into freshwater medium with acetate (10 mM) as the donor and fumarate (40 mM) as acceptor and incubated at 25 °C for 3 days. Planktonic cells were removed and culture tubes were washed twice with Milli-Q water. Cells attached to the sides of the tubes were stained with 10 mL 0.1% crystal violet solution for 10 min and then washed twice with Milli-Q water. The crystal violet stain bound to cells on the sides of the tubes was then solubilized in 10 mL ethanol overnight. After solubilization, samples were measured at 580 nm in a Genesys 2 spectophotometer (Spectronic Instruments) as previously described (Leang et al., 2013).

### Fe(II) measurements

Growth on Fe(III)-citrate and Fe(III)-oxide was monitored by measuring formation of Fe(II). Briefly, 0.1 mL of culture at various time points was removed aseptically and diluted in 0.9 mL 0.5 N HCl. Samples were then incubated for 1 h to solubilize the Fe(II) and measured with a ferrozine assay at an absorbance of 562 nm in a Genesys 2 spectophotometer (Spectronic Instruments) as previously described (Lovley and Phillips, 1987).

## Results and discussion

### Adaptive evolution during long-term growth on anode

Cells from the electrode biofilm were collected, and serially diluted into Fe(III) oxide-acetate medium. The highest dilution with growth (10^−5^) was then streaked onto solid agar plates containing acetate as the electron donor and fumarate as the electron acceptor. Five of the colonies selected, designated strains LT1-LT5 were studied in detail.

In contrast to the major differences in the strain KN400 and strain PCA genomes (15,115 total single nucleotide polymorphisms (SNPs)), which demonstrated that KN400 underwent purifying selection long ago (Butler et al., 2012), each of the LT1-LT5 strains contained only 115–122 total SNPs relative to KN400, consistent with very recent divergence (Table 1; Supplementary Table S2). However, despite their small SNP burden, these strains exhibited an extremely high LOF (loss of function) mutation frequency (14–15%) and elevated dN/dS (nonsynonymous/synonymous substitution) ratios (0.735–0.776) when they were compared to strain KN400 (Table 1). This pattern of few total mutations but a disproportionately high fraction of nonsynonymous and LOF variants reflects strong, short-term directional selection.

**Table 1:** Genome-wide mutation statistics for KN400 relative to PCA and for five evolved LT strains relative to KN400.

Comparison	Total SNPs	Ts/Tv	Genome wide dN/dS	Total functional SNPs	Genome mutational background (SNPs/kb)	Genes with functional SNPs	Gene hotspots	LOF mutations	LOF %
KN400 vs PCA	15,115	2.50	0.364	3,651	1.052	820	369	28	0.764%
LT1 vs KN400	120	2.76	0.776	41	0.01229	32	32	6	14.29%
LT2 vs KN400	120	2.76	0.776	41	0.01229	32	32	6	14.29%
LT3 vs KN400	122	3.26	0.75	42	0.01243	32	32	6	13.95%
LT4 vs KN400	115	3.11	0.735	39	0.01154	30	30	6	15%
LT5 vs KN400	115	3.11	0.735	39	0.01154	31	30	6	15%

Reported values include the total number of SNPs, transition/transversion (Ts/Tv) ratios, genome wide dN/dS (nonsynonymous/ synonymous substitution) ratios, total functional SNPs (missense, frameshift, or insertion/deletion mutations), genome mutational background (SNPs/kb), number of genes with functional SNPs, gene hotspots (genes with ≥3X mutations than genome background), number of genes with loss of function (LOF) mutations, and LOF frequency (LOF %; the proportion of LOF mutations among all mutations that affect coding sequences). KN400 exhibits a high overall SNP burden but low dN/dS and LOF% consistent with long-term purifying selection and ancient divergence from PCA. LT strains show low total SNP counts but higher dN/dS values and disproportionately high LOF%, indicating recent, targeted adaptive evolution following divergence from KN400.

There was a moderate genome size reduction of 2.3% (3,814,128 bp vs. 3,726,411 bp) when KN400 was compared to PCA, followed by a further 0.32% reduction in genome size in the LT strains (3,726,411 vs. ~ 3,714,300), suggesting modest, stepwise genome reduction consistent with early stages of genome streamlining. Alignment of the genomes revealed eight large gaps in the KN400 genome relative to PCA, corresponding to regions predicted to encode proteins with nonessential functions, including transposons, CRISPR-Cas system proteins, bacteriophage exclusion proteins, phage integrases, conjugative transfer proteins, Type III restriction modification proteins, heavy metal efflux proteins, retron-type reverse transcriptases, and hypothetical proteins. In contrast, no obvious large deletions were apparent when the LT genomes were compared to KN400.

All the LT genomes carried 39–42 functional SNPs and 31–33 hotspots (genes with ≥3X the number of functional mutations than the genome background), with highly similar sets of affected genes (Table 2; Supplementary Table S2). This shared mutation profile indicates that each of these isolates originated from a single successful KN400 sublineage that underwent a clonal sweep within the electrode biofilm rather than multiple independent parallel evolutionary events.

**Table 2:** Hotspot genes in LT genomes compared to the KN400 parent genome.

Locus ID	Gene	Annotation	Presence of hotspot
KN400_0209	*tex*	Tex RNA binding protein	All LT strains
KN400_0250		Cadherin domain surface protein	All LT strains
KN400_0383	*flhB*	Flagellar protein FlbB	All LT strains
KN400_0445		Hypothetical protein	All LT strains
KN400_0686		KAP NTPase	All LT strains
KN400_0713	*ehrA-2*	Hydrogenase-4 component B	All LT strains
KN400_0718	*ehrB*	Formate hydrogenlyase subunit 4	All LT strains
KN400_0731		Fibronectin type III surface protein	All LT strains
KN400_0873		Serine protease	All LT strains
KN400_0956		Bacteriophage tail sheath protein	All LT strains
KN400_1040		Cytochrome c, 1 heme-binding site	All LT strains
KN400_1046	*aplB*	Cation/acetate symporter	All LT strains
KN400_1048	*actP3*	Cation/acetate symporter	All LT strains
KN400_1277	*mcp025*	Methyl-accepting chemotaxis protein	All LT strains
KN400_1559	*recB*	Exodeoxyribonuclease V beta subunit	All LT strains
KN400_1583		SkfB/NifB/PqqE family protein	All LT strains
KN400_1858	*glnB*	Nitrogen regulatory protein P-II	All LT strains
KN400_1937	*ilvB*	Acetolactate synthase, large subunit	All LT strains
KN400_1971		Fibronectin type III surface protein	All LT strains
KN400_2025		Hypothetical protein	Only LT3
KN400_2133		Predicted secreted hydrolase	All LT strains
KN400_2162		HEAT-like repeat-containing protein	LT1 and LT2
KN400_2297	*aplC*	Cation/acetate symporter	All LT strains
KN400_2343		Group II intron maturase	All LT strains
KN400_2362	*tesA*	Acyl-CoA thioesterase I	All LT strains
KN400_2396	*sucB*	2-oxoglutarate dehydrogenase	All LT strains
KN400_2429	*kup2*	KUP system potassium uptake protein	Only LT3
KN400_2431		Zn/Ni/Co-binding GTPase	All LT strains
KN400_2483	*nspC*	Carboxyaminopropylagmatine decarboxylase	All LT strains
KN400_2550		Hypothetical protein	All LT strains
KN400_2827	*omcA*	Cytochrome c, 27 heme-binding sites	All LT strains
KN400_2981	*flgL*	Flagellar hook-filament junction protein FlgL	All LT strains
KN400_3190	*pucG*	Predicted phosphoserine aminotransferase	All LT strains
KN400_3238	*glcF-1*	Glycolate dehydrogenase iron–sulfur subunit	All LT strains

Genes were only considered hotspots if they had accumulated ≥3 times the number of functional SNPs in their genome than the genome mutational background.

As observed for strain KN400, the LT strains contained numerous mutations in the genes encoding OmcS and OmcE relative to strain PCA, whereas the OmcZ sequence remained conserved. Further analysis of the OmcS genes in KN400 and LT strains identified SNPs predicted to alter 11 amino acid residues, six of which involve substitutions with markedly different chemical properties (Table 3; Supplementary Table S2).

**Table 3:** Mutations in *c*-type cytochrome genes in KN400 and LT strains compared to PCA that would change amino acid composition.

PCA locus ID	Gene	Total # of SNPs in gene	# Amino acid changes in protein	# Amino acid category changes	Cell localization
GSU0222	*coxB*	42	10	6	CytoplasmicMembrane
GSU0274	*cbcL*	1	1	1	CytoplasmicMembrane
GSU0702		139	42	25	Extracellular
GSU2724	*extG*	71	19	13	Extracellular
GSU2935	*extM*	31	11	9	Extracellular
GSU0618	*omcE*	38	14	7	Extracellular
GSU0701	*omcJ*	28	5	4	Extracellular
GSU1228	*omcI*	1	1	1	Extracellular
GSU1761	*pgcA*	12 bp deletion	4	4	Extracellular
GSU2203	*omcK*	2	1	1	Extracellular
GSU2503	*omcT*	16	2	1	Extracellular
GSU2504	*omcS*	37	11	6	Extracellular
GSU2513		23	11	7	Extracellular
GSU2731	*omcC*	2	2	2	Extracellular
GSU2808		6	2	1	Extracellular
GSU2898	*omcN*	53	25	19	Extracellular
GSU2899		5	1	1	Extracellular
GSU2912	*omcO*	38	6	4	Extracellular
GSU2299		31	11	8	Periplasmic
GSU2501	*oscC*	39	9	5	Periplasmic
GSU2725	*extF*	28	10	2	Periplasmic
GSU2934	*cbcN*	3	1	1	Periplasmic
GSU2937	*extK*	55	12	4	Periplasmic
GSU3274	*pccH*	Truncation at CDS position 30			Periplasmic
GSU2495	*oscI*	37	14	9	Periplasmic
GSU0615		7	4	3	Periplasmic
GSU2494	*oscJ*	Frameshift at position 868			Periplasmic
GSU2813	*ccpA*	21	5	3	Periplasmic
GSU3218		20	4	3	Periplasmic
GSU3334		1	1	0	Periplasmic
GSU0616		19	5	3	Unknown
GSU1334		75	21	12	Unknown
GSU2210		31	12	5	Unknown
GSU2811		77	13	7	Unknown
GSU3221		3	1	1	Unknown
GSU3226		1	1	1	Unknown

Total # of SNPS refers to the total number of base changes in the gene. # of amino acid changes refers to the number of SNPs that alter an amino acid residue in the protein product. # of amino acid category changes refers to the number of amino acid changes that result in a change in chemical properties of the side group (e.g., acidic side chain changed to a basic side chain). All SNPs have >300 fold coverage, variant frequencies >90% and E values of 0. Further details can be found in Supplementary Table S2A.

**Figure 1:**
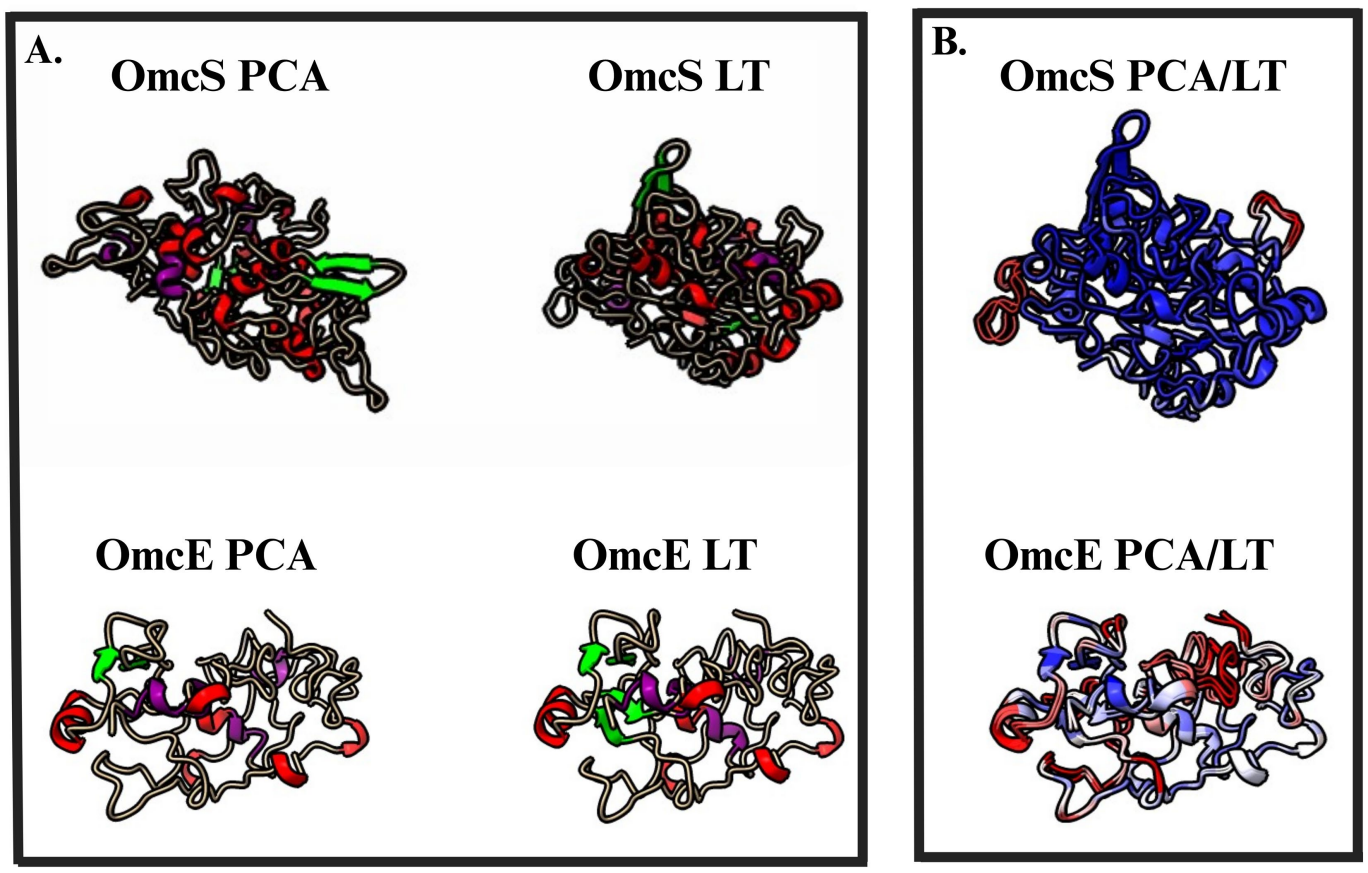
Predicted differences between the structures of OmcS and OmcE from the LT strains and strain PCA. **(A)** Predicted structure of mature (signal sequence removed) OmcS and OmcE proteins predicted by AlphaFold. Red represents *α*-helices, green represents *β*-strands, and purple represents heme prosthetic groups. **(B)** Superimposed model of the two predicted protein structures generated using MatchMaker and colored by per-residue Cα-RMSD method. Color is ramped from blue to red. Residues are colored on a continuous gradient: Blue indicates the highest similarity (RMSD <0A), white indicates intermediate difference (RMSD ~ 1), and red indicates the largest structural difference (RSD > 2 A). Statistical measurements from these models are available in Supplementary Figure S2 and Supplementary Table S3.

Structural modeling predicted differences in both the secondary and tertiary structures of OmcS in the LT and KN400 strains compared to PCA. Superimposition of the LT and PCA OmcS models using Matchmaker in ChimeraX, along with TM-score analysis (Zhang and Skolnick, 2004), indicated that the overall protein fold is highly conserved (RMSD (root mean square deviation) = 0.56 Å across 407 C*α* atoms; TM score = 0.9) (Figure 1; Supplementary Figure S2). However, residue-level comparison of DSSP assignments showed that secondary structures differed at 11 of 407 positions (2.7% of residues). Most differences involved coil-to-sheet or coil-to-helix transitions, with a few helix-to-non-helix substitutions. Additionally, the average *β*-strand length was slightly greater in OmcS from LT and KN400 compared to PCA (paired *t*-test, *p* = 0.001).

Structural mutations were also identified in three paralogous genes encoding OmcS-like proteins (Shen et al., 2025): *omcT* (GSU2503), *oscC* (GSU2501), and *omcJ* (GSU0701) (Table 2; Supplementary Figure S2). The *omcS*, *omcT* and *oscC* genes are part of the larger osc (omcS companion) operon, which is proposed to encode proteins involved in OmcS nanowire folding (OscH), secretion (OscE, OscF, OscG) and morphology (OscD) (Shen et al., 2025). Numerous missense mutations predicted to alter secondary and tertiary structure were detected across all genes in this operon in both the KN400 and LT strains (Supplementary Figure S3). The proteins OscE, OscF, and OscG, which have been suggested to be required for OmcS secretion (Shen et al., 2025), contained 22, 4, and 24 amino acid substitutions, respectively. Structural modeling predicted deviations in both secondary and tertiary structure relative to PCA (Supplementary Figure S3). In addition, OmcD, which has been suggested to influence nanowire morphology (Shen et al., 2025), exhibited 45 amino acid substitutions. Superimposition of PCA and LT OscD models yielded an RMSD of 61.58 Å across 473 aligned amino acids (TM-score: 0.11), indicating substantial divergence in overall protein architecture.

Previous studies have demonstrated that OmcS nanowires are not required for growth in anode biofilms (Nevin et al., 2009; Malvankar et al., 2012a,b) or during Fe(III) oxide reduction (Liu et al., 2022; Schwarz et al., 2024). In fact, deletion of the gene for OmcS yielded strains with increased biofilm conductivities (Malvankar et al., 2012a,b). Therefore, it is not surprising that mutations accumulated in this protein that is not important for current production.

OmcE (GSU0618), another outer surface multiheme *c*-type cytochrome proposed to form conductive nanowires (Wang et al., 2022), carried 38 SNPs in both LT and KN400 strains, 14 of which were nonsynonymous. Despite this sequence variation, structural modeling indicated that the overall fold of OmcE remained conserved (RMSD = 0.8 Å, TM-score = 0.97). However, secondary structure analysis revealed a modest but statistically significant reduction in α-helical content in OmcE from LT and KN400 relative to PCA (2.4% decrease; *p*-value = 0.02). In addition, the average distance between heme groups was slightly greater in LT and KN400 compared to PCA (mean increase = 0.1 Å; *p* = 0.05) (Figure 1B; Supplementary Table S3). OmcE is also not essential for either current production or Fe(III) oxide reduction (Nevin et al., 2009; Malvankar et al., 2012a,b; Schwarz et al., 2024).

None of the genes for assembly of pili had mutations in the KN400 or LT strains compared to strain PCA. There were, however, mutations in three out of five sets of genes coding for porin cytochrome conduits in the KN400 and LT strains compared to PCA (Supplementary Figure S4). Transcriptional analysis has indicated that the OmbBOmaBOmcB complex (GSU2737-GSU2739) is important in biofilms generating high current densities (Nevin et al., 2009), whereas short-term analysis of anode colonization with gene-deletion mutants (Otero et al., 2018) indicated that initial growth and current production is somewhat reduced in the absence of ExtABCD (GSU2642-GSU2645). Notably, there were no mutations in the genes for the components of either of these porin-cytochrome conduits. However, there were mutations in critical genes of the other three porin-cytochrome conduits (*extEFG*: GSU2724-2726; *omabcC*: GSU2731-2733; *extIJKLM*: GSU2935-2939) that have not been implicated in current production (Supplementary Figure S4).

The *coxABCD* gene cluster (GSU0219-GSU0222) encodes the cytochrome c oxidase complex, which is typically involved in aerobic respiration. Its role in *G. sulfurreducens* is unknown, but it might be involved in growth at low oxygen concentrations (Lin et al., 2004). The gene encoding cytochrome c oxidase subunit II (CoxB) was substantially mutated in KN400 and LT strains. Six substitutions resulted in changes to amino acid residues that were predicted to dramatically alter the tertiary structure of the protein (RMSD = 19.61 Å, TM-score = 0.16) (Supplementary Table S2; Supplementary Figure S5). In addition to *coxB*, the other genes in the cytochrome c oxidase complex (*coxA*, *coxC*, and *coxD*) also have multiple mutations that could impair function. This may reflect a diminished need to process oxygen under the strict anaerobic growth conditions found in the bioelectrochemical system.

**Figure 2:**
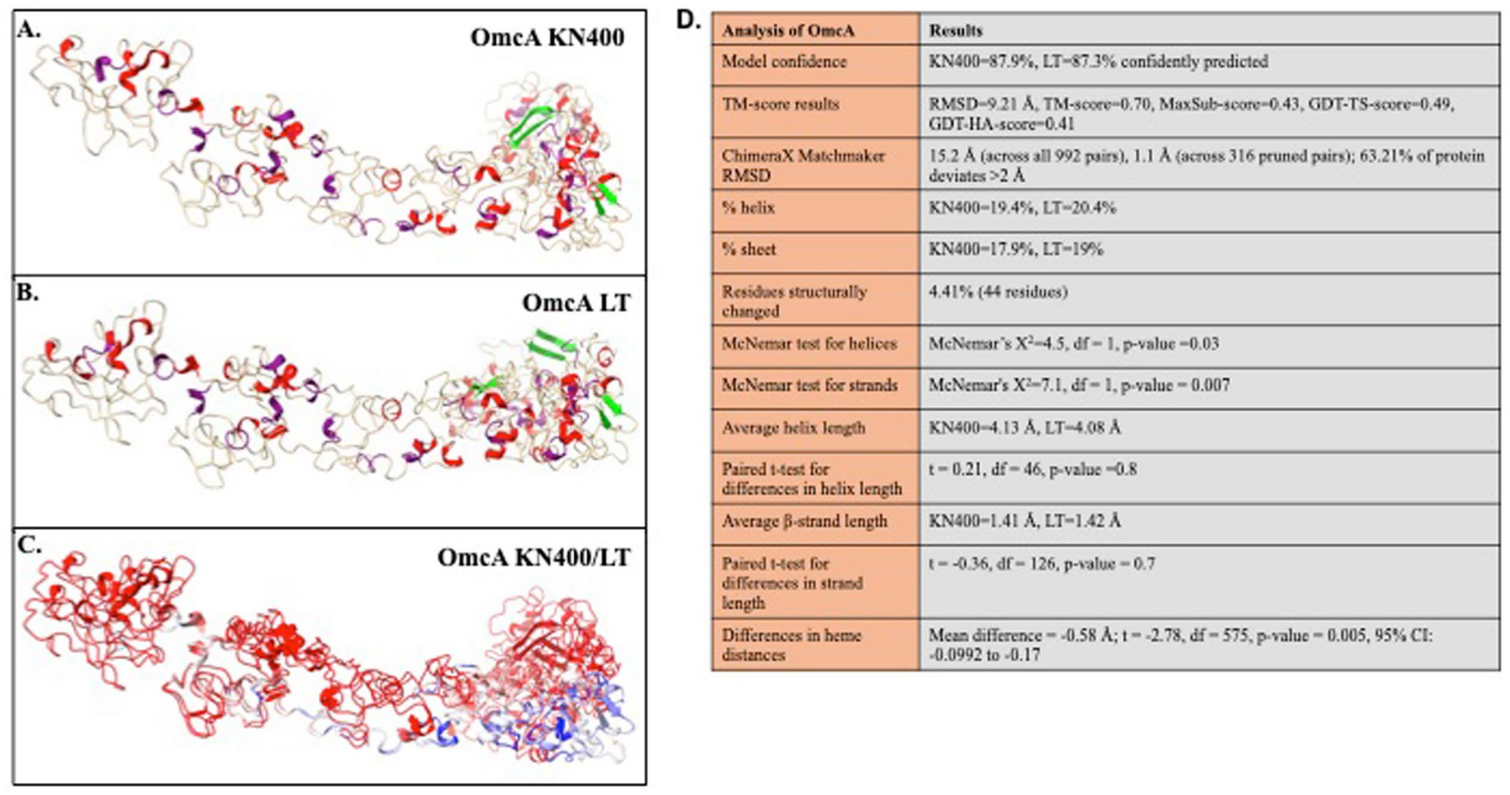
Predicted structural differences in mature OmcA from the LT strains and strain KN400 (signal sequence removed). **(A)** Predicted structure of mature OmcA from KN400; **(B)** Predicted structure of mature OmcA from LT; **(C)** Superimposed model of the two predicted protein structures generated using MatchMaker and colored by per-residue Cα-RMSD method. Color is ramped from blue to red. Residues are colored on a continuous gradient: Blue indicates the highest similarity (RMSD < 0 Å), white indicates intermediate difference (RMSD ~ 1 Å), and red indicates the largest structural difference (RSD > 2 Å). **(D)** Statistics revealing differences in secondary and tertiary structures in LT and KN400 OmcA proteins.

In addition to all of these previously reported genes having major mutations in both KN400 and LT strains compared to PCA, several functional mutations were also identified in direct comparisons between KN400 and LT strains (Table 2). All LT strains carried a missense mutation in a gene encoding an outer surface multiheme c-type cytochrome (*omcA*; KN400_2827) that changed a proline to an alanine. Despite being a single amino acid change, structural modeling predicted effects on both secondary and tertiary structure (Figure 2). Superimposition of the KN400 and LT OmcA models revealed structural divergence (RMSD = 15.156 Å across all 992 aligned amino acids; TM-score = 0.7). In addition, inter-heme distances were smaller in the LT variant (mean decrease ~0.6 Å; *p* = 0.005), and significant differences were detected in both *α*-helix (*p* = 0.03) and *β*-sheet (*p* = 0.007) content, indicating measurable alterations in overall protein architecture. This protein is not required for electrode growth or growth on Fe(III)-oxides (Kim et al., 2006). Growth on electrodes imposes strong selective pressure for traits that enhance EET. In contrast, genes that are not required under these conditions (e.g., *omcA*) are likely subject to relaxed selection, permitting the accumulation of mutations through genetic drift.

Mutations were also identified within two GEMM riboswitches and one adenosylcobalamin-responsive riboswitch in all LT strains (Supplementary Tables S2B–F). In *Geobacter*, GEMM riboswitches bind the secondary messenger cyclic-di-GMP (c-di-GMP) and regulate expression of genes involved in EET, biofilm formation, and exopolysaccharide production (Kellenberger et al., 2015; Tan et al., 2022). Adenosylcobalamin-responsive riboswitches respond to intracellular levels of coenzyme B12 and control expression of genes involved in cobalamin metabolism.

The two GEMM riboswitches were located upstream of KN400_1038, which encodes a hypothetical protein, and KN400_1971, which encodes a fibronectin III (FNIII) domain-containing protein. Proteins containing FNIII domains are frequently associated with surface attachment and biofilm formation (Mccourt et al., 2014). The adenosylcobalamin-responsive riboswitch was positioned upstream of an operon encoding enzymes involved in cobalamin biosynthesis. Disruption of this riboswitch could reduce feedback repression of the biosynthetic pathway, potentially increasing intracellular cobalamin levels. Because cobalamin serves as a cofactor for methionine synthase, elevated cobalamin availability could enhance methionine production and subsequently increase S-adenosylmethionine (SAM) levels. As SAM functions as a universal methyl donor in numerous regulatory processes, altered SAM availability could indirectly influence gene expression patterns, including those associated with surface attachment, biofilm formation, and EET.

Additional evidence for genome reduction toward streamlining in the LT strains was the observation that several paralogous genes were mutational hotspots. For example, three of the four cation/acetate symporter genes (*aplC*, KN400_2297; *aplB*, KN400_1046, *aplA*, KN400_1048) contained mutations (Table 2; Supplementary Figure S6). AplA was most significantly altered, and superimpostion of KN400 and LT AplA models yielded an RMSD of 9.33 Å across 636 aligned residue pairs (TM-score = 0.49), suggesting moderate structural alteration. These three transporters are more highly expressed under acetate-limiting conditions and form a phylogenetic clade designated Group I, whereas the fourth transporter, *alpD* (KN400_0507), classified as a Group II transporter, is not induced under acetate limitation (Risso et al., 2008). Notably, *aplD* was the only acetate transporter gene that lacked mutations in the LT strains. The absence of mutations in *aplD* suggests that this transporter may serve a constitutive role in acetate uptake and could be under stabilizing selection to preserve basal metabolic function. However, further studies are needed to examine this possibility.

Missense mutations were also detected in two subunits of the Ehr (Ech hydrogenase related) complex. The Ehr complex is homologous to Ech hydrogenase found in a diversity of H_2_ metabolizing organisms, but it lacks the catalytic NiFe binding center and is therefore not capable of H_2_ oxidation (Coppi, 2005). Thus, Ehr was already nonfunctional in KN400 prior to long-term electrode cultivation. Because hydrogenase activity is not required for growth on acetate with electrodes, additional mutations in *ehr* genes would not impair fitness under these conditions. Instead, these genes would be expected to accumulate mutations through relaxed selection and genetic drift, potentially contributing to genome streamlining.

### Other genes with polymorphisms

A major difference between strain KN400 and strain PCA is that strain KN400 expresses functional flagella, whereas strain PCA does not (Ueki et al., 2012). However, there were nonsynonymous mutations in two flagellar genes of the LT strains, suggesting a lack of functional flagella (Table 2). For example, the gene coding for the flagellar protein FlbB (KN400_0383) has 2 missense mutations and the gene for the flagellar hook-filament junction protein FlgL (KN400_2981) has 1 missense mutation. These mutations are predicted to cause changes in secondary and tertiary structures (Supplementary Figure S6). This accumulation of mutations in genes associated with flagella-based motility is consistent with the fact that motility is not required in mature biofilms (Guttenplan and Learns, 2014; Lewis et al., 2022). However, further examination of flagella in these strains is needed to determine their functional status in the LT strains.

### Mutations in regulatory genes found in KN400 and LT strains

Numerous mutations were also present in regulatory genes in KN400 and LT strains including those encoding signal transduction proteins and transcriptional regulators (Supplementary Table S2). Changes in regulatory systems are a common strategy for rapid adaptation because they can rewire entire transcriptional networks and induce broad shifts in gene expression patterns. This systems-level change is much more efficient than acquiring multiple independent mutations in structural or metabolic genes.

At least 274 genes in the *G. sulfurreducens* genome encode signal transduction proteins, and 74 of these (27%) in the LT and KN400 strains contained mutations predicted to alter amino acid composition and likely protein function. Among the 77 genes encoding proteins with histidine kinase function, 23 (30%) contained amino acid altering mutations. Histidine kinases initiate bacterial two-component signaling systems by sensing stimuli, autophosphorylating a conserved histidine residue, and then transferring the phosphoryl group to a response regulator that can modulate gene expression. Of the 23 mutated histidine kinases, 13 (54%) contained missense mutations within the conserved phosphoacceptor (pfam00512) or ATPase (pfam02518) domains required for autophosphorylation. In addition, one histidine kinase (GSU2507) previously shown to repress transcription of *omcB* and *pilA* during growth on Fe(III) (Schwarz et al., 2024) accumulated multiple mutations in the LT and KN400 strains.

Similarly, among 76 response regulators involved in phosphorelay that contain receiver domains (pfam00072) or histidine phosphotransfer (Hpt) domains (pfam01627), 14 (18%) contained amino acid-altering mutations. Of these, 11 had mutations within a receiver domain and 3 had mutations within an Hpt domain. Many response regulators also possess C-terminal DNA-binding domains (e.g., pfam00486, pfam00196) that regulate transcription upon phosphorylation. Two of these two-component system proteins contained missense mutations within their predicted DNA binding motifs.

Despite the substantial number of mutations observed across two-component regulatory systems, no mutations were detected in components of the electrode sensing network (Esn; GSU1704, GSU2220, GSU2222, and GSU3376) which is suggested to be important for electrode dependent growth (Chan et al., 2017).

Diguanylate cyclases were also affected. Of the 29 proteins containing diguanylate cyclase (GGDEF) domains (pfam00990) (Kellenberger et al., 2015), six carried nonsynonymous mutations, and four of these contained missense substitutions within the GGDEF domain. These proteins synthesize the secondary messenger c-di-GMP, a central regulator of biofilm formation (Jenal et al., 2017). In *G. sulfurreducens*, reduced intracellular levels of c-di-GMP have been shown to enhance EET by up-regulating expression of *pilA, omcS, omcZ*, *omcB*, and *omcE* (Hu et al., 2024).

Thirty-five transcriptional regulators were also significantly modified, spanning diverse families including sigma-54 dependent Fis, AraC, ArsR, BadM/Rrf2, Fur, GntR, LysR, LytTR, MarR, MerR, TetR/AcrR, and TraR/DksA. Most of these proteins can act as repressors (Hinrichs et al., 1994; Alekshun and Levy, 1999; Hantke, 2001; Busenlehner et al., 2003; Peres and Harwood, 2006; Maddocks and Oyston, 2008; Schleif, 2010; Embree et al., 2014; Zhu et al., 2023). Mutations in AraC/TetR regulators was previously observed during adaptive evolution of *G. sulfurreducens* in co-culture with *Pseudomonas aeruginosa* (Semenec et al., 2020). OmcZ, a critical outer-surface cytochrome involved in electron transfer to electrodes, is under the control of the Fur repressor (Inoue et al., 2010; Embree et al., 2014). Mutations in Fur may therefore contribute to the enhanced *omcZ* expression observed in the LT strains.

### LT strains grew significantly faster on Fe(III) oxide

**Figure 3:**
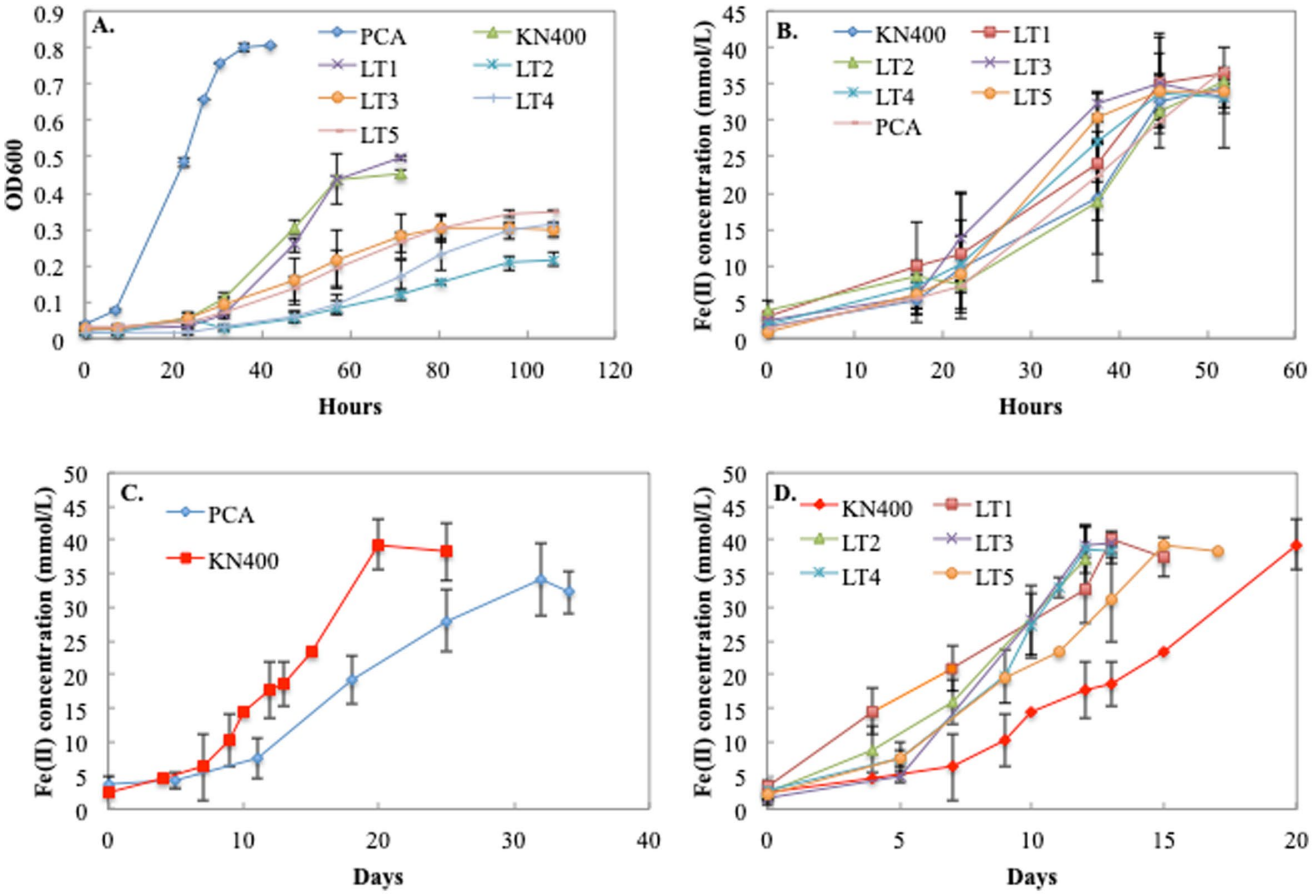
Growth of various *G. sulfurreducens* strains (PCA, KN400, LT1, LT2, LT3, LT4, and LT5) with acetate (20 mM) as the electron donor and different electron acceptors: **(A)** Growth with fumarate (40 mM); **(B)** Growth with Fe(III) citrate (50 mM); **(C)** Growth of PCA and KN400 with Fe(III) oxide (50 mM); **(D)** Growth of KN400 and LT strains with Fe(III) oxide (50 mM). All points represent the average and standard deviation of triplicate samples and statistical significance was determined with unpaired *t*-tests done with the R-stats package. Average fumarate specific growth rates (k): PCA = 0.07 h^−1^, KN400 = 0.12 h^−1^, LT1 = 0.15 h^−1^, LT2 = 0.22 h^−1^, LT3 = 0.26 h^−1^, LT4 = 0.21 h^−1^, LT5 = 0.19 h^−1^. Average Fe(III)-citrate specific growth rates (*k*): PCA = 0.06 h^−1^, KN400 = 0.06 h^−1^, LT1 = 0.05 h^−1^, LT2 = 0.045 h^−1^, LT3 = 0.06 h^−1^, LT4 = 0.06 h^−1^, LT5 = 0.07 h^−1^. Average Fe(III)-oxide growth rates (*k*): PCA = 0.07 day^−1^, KN400 = 0.12 day^−1^, LT1 = 0.15 day^−1^, LT2 = 0.22 day^−1^, LT3 = 0.26 day^−1^, LT4 = 0.22 day^−1^, LT5 = 0.27 day^−1^.

The LT strains and strain KN400 grew significantly slower with fumarate as the electron acceptor than strain PCA, and all but one of the LT strains (LT1) grew slower on fumarate than strain KN400 (Figure 3A). Growth rates for all strains were similar with soluble Fe(III) citrate as the electron acceptor (Figure 3B). As previously reported (Ueki et al., 2012), strain KN400 grew faster on Fe(III) oxide than strain PCA (Figure 3C). On average, the LT strains grew 1.8-fold faster (*p* = 0.0015) than strain KN 400 (Figure 3D). In contrast to Fe(III) citrate, which can be reduced at the outer-cell surface, effective reduction of Fe(III) oxides requires conductive filaments that extend EET beyond the cell surface (Levar et al., 2013; Lovley and Holmes, 2022; Lovley, 2026). These results indicate that the initial short-term selection of strain KN400 on the anode and the subsequent adaptive evolution of strains over 17 years of current production specifically selects for features that enhance long-range EET.

### Higher expression of PilA, OmcZ and OmcB genes

**Figure 4:**
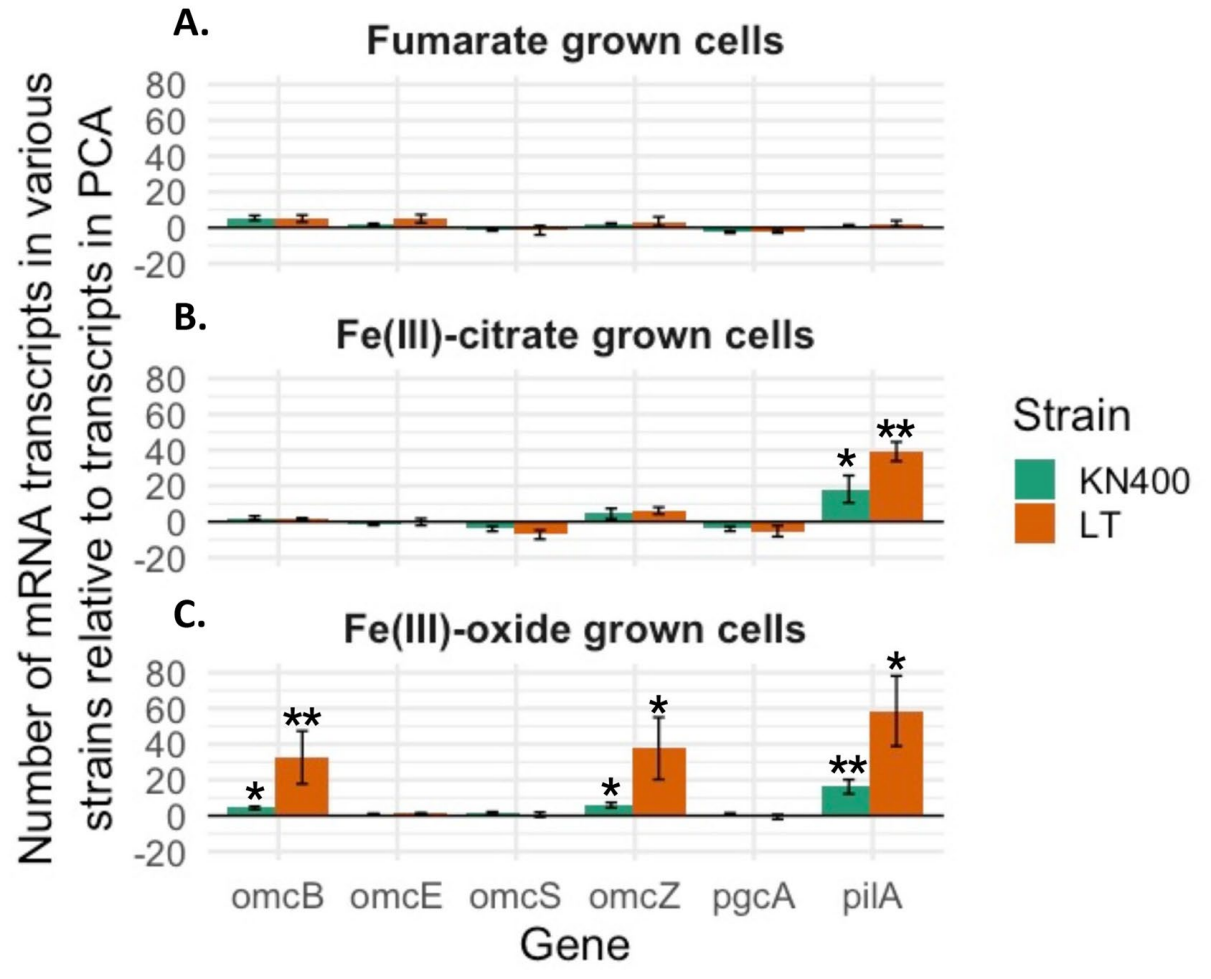
Quantitative RT-PCR results showing relative gene expression of EET genes in KN400 and the average of all five LT strains (LT1, LT2, LT3, LT4, and LT5) compared to PCA cells grown under various conditions. All transcripts were normalized against the housekeeping gene, *recA*, and then compared to *recA*-normalized PCA values. **(A)** qRT-PCR of transcripts from mRNA extracted from cells grown on acetate and fumarate during mid-log phase; **(B)** qRT-PCR of transcripts from mRNA extracted from cells grown on acetate and Fe(III) citrate during mid-log phase; **(C)** qRT-PCR of transcripts from mRNA extracted from cells grown on acetate and Fe(III)-oxide during mid-log phase. All values and standard deviations represent triplicate biological and technical replicates.

Quantitative RT-PCR revealed that during growth on fumarate, transcript abundances for genes coding for possible EET proteins (OmcS, PgcA, PilA, OmcZ, OmcE, OmcB) were comparable for the LT strains, strain KN400, and strain PCA (Figure 4A). During growth on Fe(III) citrate, transcripts for the PilA gene were significantly higher for the LT strains than for strain K400, which had significantly higher PilA gene transcripts than strain PCA (Figure 4B). The transcript abundance of strain KN400 and the LT strains for the PilA, OmcZ, and OmcB genes were significantly higher than strain PCA during growth on Fe(III) oxide (Figure 4C). The LT strains also had higher expression of these genes than strain KN400.

Adaptive evolution for growth on Fe(III) oxides selects for increased expression of the outer-surface cytochrome PgcA (Tremblay et al., 2011; Smith et al., 2014), but PgcA does not appear to be important for electron transfer to electrodes (Zacharoff et al., 2017). Levels of PgcA transcripts were comparable in all strains (Figure 4). These results demonstrate a selection for the ability to specifically express genes for key components of high-density current production during growth on the anode.

### Attachment of strain KN400 and LT strains

**Figure 5:**
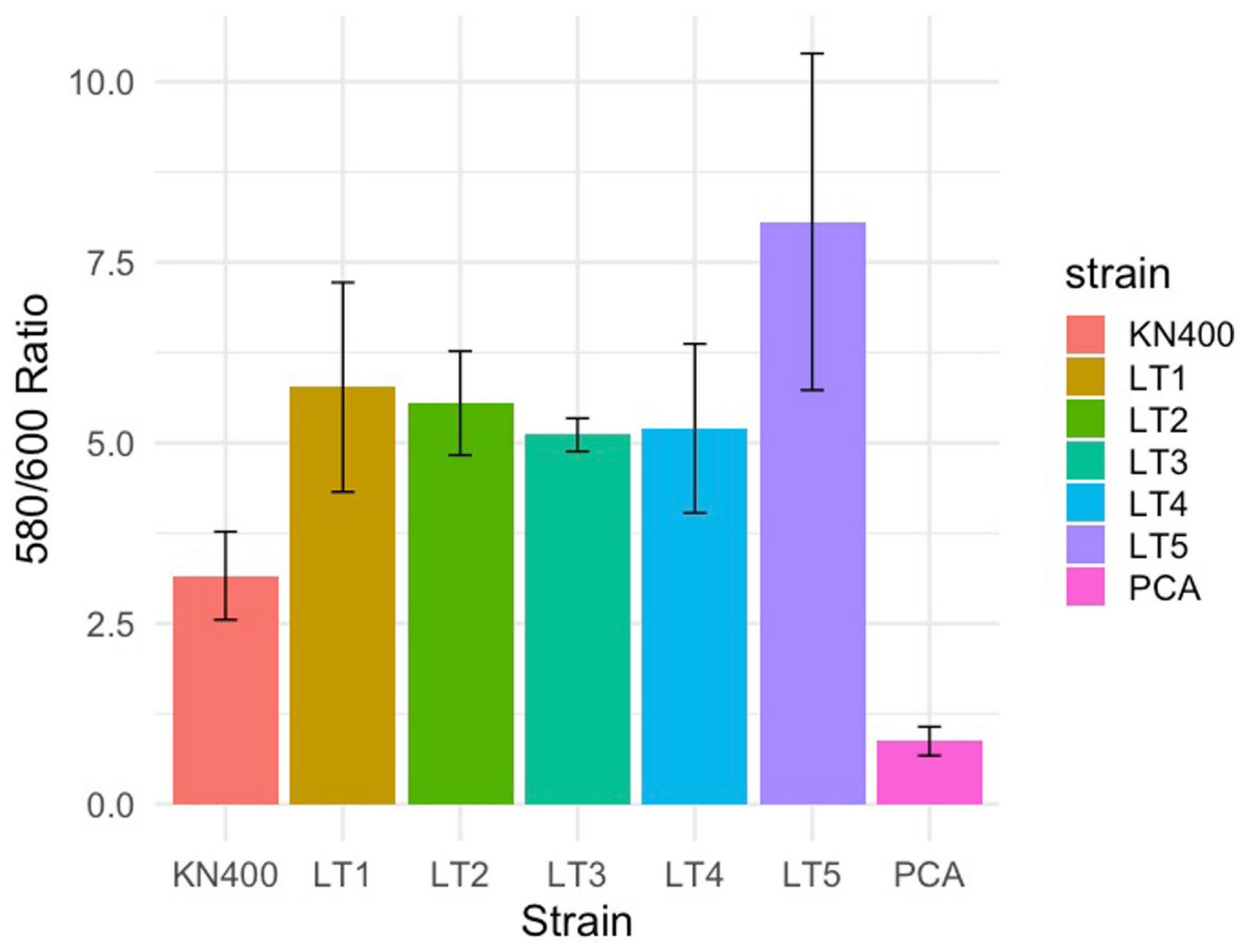
Differences in biofilm formation between the various *G. sulfurreducens* strains determined with the crystal violet assay. Absorbance at 580 nm of crystal violet from the biofilm solubilized in ethanol divided by absorbance at 600 nm of the culture at early stationary phase. All data represent the average and standard deviations from 5 replicates for each strain.

Strain KN400 exhibits greater surface-associated biomass on glass than strain PCA, despite expressing lower concentrations of exopolysaccharides (Yi et al., 2009). The LT strains showed an even greater level of crystal violet staining than KN400 (Figure 5), indicating increased surface-associated biofilm biomass and suggesting selection for enhanced surface attachment. However, further studies under EET conditions using methods that directly assess biofilm structure and activity will be necessary to better define strain-specific differences.

## Conclusion

The results demonstrate that although the *G. sulfurreducens* mechanisms for long-range EET were originally evolved for purposes other than electron transfer to electrodes, long-term growth as a current-producing biofilm selected for further optimization of the primary EET route that leads to high current densities when cells are first inoculated into a bioelectrochemical system. During years of continuous growth on an anode, the KN400 strain further evolved to streamline its genome and enhance the expression of key EET components. *G. sulfurreducens* generation of high current densities requires: porin-cytochrome conduits to transport electrons to the outer cell surface, e-pili that are required for long-range electron transport through the biofilm (i.e., biofilm conductivity), and OmcZ to facilitate electron transfer from the biofilm to the anode. There were no mutations in any of these systems and the capacity for expression of the relevant genes was substantially increased under growth conditions that required long-range EET. In contrast, genes of outer-surface cytochromes (OmcS, OmcE, OmcA), flagella proteins, and porin-cytochrome conduit proteins that are not required for current production were heavily mutated.

Adoption of an alternative route for electron transfer to electrodes was not readily apparent. For example, if, as commonly proposed, OmcS and OmcE serve as important conduits for long-range EET (Tabari and Hochbaum, 2025), it might be expected that there would be a selection for increased expression of these proteins. However, both cytochromes were highly mutated. There was no increased expression of PgcA, which can enhance Fe(III) oxide reduction (Tremblay et al., 2011; Smith et al., 2014).

In order to obtain results on EET mechanisms that can be extrapolated to long-running systems, it is important to carry out incubations long enough to reach steady-state maximum current production because the selective pressures for short-term and long-term current production may differ (Nevin et al., 2009). However, the finding that the basic route for *G. sulfurreducens* current production under initial steady state conditions was amplified, but basically the same after 17 years of operation of the bioelectrochemical system suggests that the understanding of EET mechanisms derived from short-term incubations of steady-state systems producing stabilized maximum currents can provide insights into EET mechanisms relevant to long-term applications.

## Data Availability

The datasets presented in this study can be found in online repositories. The names of the repository/repositories and accession number(s) can be found in the article/Supplementary material.

## References

[ref1] Adhikari R. Y. Malvankar N. S. Tuominen M. T. Lovley D. R. (2016). Conductivity of individual Geobacter pili. RSC Adv. 6, 8354–8357. doi: 10.1039/C5RA28092C

[ref2] Alekshun M. N. Levy S. B. (1999). The mar regulon: multiple resistance to antibiotics and other toxic chemicals. Trends Microbiol. 7, 410–413. doi: 10.1016/S0966-842X(99)01589-9, 10498949

[ref3] Bond D. R. Lovley D. R. (2003). Electricity production by *Geobacter sulfurreducens* attached to electrodes. Appl. Environ. Microbiol. 69, 1548–1555. doi: 10.1128/AEM.69.3.1548-1555.2003, 12620842 PMC150094

[ref4] Bond D. R. Strycharz-Glaven S. M. Tender L. M. Torres C. I. (2012). On electron transport through Geobacter biofilms. ChemSusChem 5, 1099–1105. doi: 10.1002/cssc.201100748 22615023

[ref5] Busenlehner L. S. Pennella M. A. Giedroc D. P. (2003). The SmtB/ArsR family of metalloregulatory transcriptional repressors: structural insights into prokaryotic metal resistance. FEMS Microbiol. Rev. 27, 131–143. doi: 10.1016/S0168-6445(03)00054-8, 12829264

[ref6] Butler J. Young N. Aklujkar M. Lovley D. (2012). Comparative genomic analysis of *Geobacter sulfurreducens* KN400, a strain with enhanced capacity for extracellular electron transfer and electricity production. BMC Genomics 13:471. doi: 10.1186/1471-2164-13-471, 22967216 PMC3495685

[ref7] Caccavo F. Jr. Lonergan D. J. Lovley D. R. Davis M. Stolz J. F. Mcinerney M. J. (1994). *Geobacter sulfurreducens* sp. nov., a hydrogen- and acetate-oxidizing dissimilatory metal-reducing microorganism. Appl. Environ. Microbiol. 60, 3752–3759. doi: 10.1128/aem.60.10.3752-3759.1994, 7527204 PMC201883

[ref8] Chan C. H. Levar C. E. Jimenez-Otero F. Bond D. R. (2017). Genome scale mutational analysis of *Geobacter sulfurreducens* reveals distinct molecular mechanisms for respiration and sensing of poised electrodes versus Fe(III) Oxides. J. Bacteriol. 199:e00340-17. doi: 10.1128/JB.00340-17, 28674067 PMC5585712

[ref9] Chaudhuri S. K. Lovley D. R. (2003). Electricity generation by direct oxidation of glucose in mediatorless microbial fuel cells. Nat. Biotechnol. 21, 1229–1232. doi: 10.1038/nbt867, 12960964

[ref10] Childers S. E. Ciufo S. Lovley D. R. (2002). *Geobacter metallireducens* accesses insoluble Fe(III) oxide by chemotaxis. Nature 416, 767–769. doi: 10.1038/416767a, 11961561

[ref11] Coppi M. V. (2005). The hydrogenases of *Geobacter sulfurreducens*: a comparative genomic perspective. Microbiology (Reading) 151, 1239–1254. doi: 10.1099/mic.0.27535-0, 15817791

[ref12] Coppi M. V. Leang C. Sandler S. J. Lovley D. R. (2001). Development of a genetic system for *Geobacter sulfurreducens*. Appl. Environ. Microbiol. 67, 3180–3187. doi: 10.1128/AEM.67.7.3180-3187.2001, 11425739 PMC92998

[ref13] Embree M. Qiu Y. Shieu W. Nagarajan H. O'neil R. Lovley D. . (2014). The iron stimulon and fur regulon of Geobacter sulfurreducens and their role in energy metabolism. Appl. Environ. Microbiol. 80, 2918–2927. doi: 10.1128/AEM.03916-13, 24584254 PMC3993298

[ref14] Goddard T. D. Huang C. C. Meng E. C. Pettersen E. F. Couch G. S. Morris J. H. . (2018). UCSF ChimeraX: meeting modern challenges in visualization and analysis. Protein Sci. 27, 14–25. doi: 10.1002/pro.3235, 28710774 PMC5734306

[ref15] Guttenplan S. Learns D. (2014). Regulation of flagellar motility during biofilm formation. FEMS Microbiol. Rev. 37, 849–871. doi: 10.1111/1574-6976.12018, 23480406 PMC3718880

[ref16] Hantke K. (2001). Regulation of ferric iron transport in *Escherichia coli* K-12: isolation of a constitutive mutant. Mol. Gen. Genet. 182, 288–292. doi: 10.1007/BF00269672 7026976

[ref17] Hekkelman M. Salmoral D. Perrakis A. Joosten R. (2025). DSSP 4: FAIR annotation of protein secondary structure. Protein Sci. 34:e70208. doi: 10.1002/pro.70208, 40671631 PMC12268231

[ref18] Hinrichs W. Kisker C. Duvel M. Muller A. Tovar K. Hillen W. . (1994). Structure of the Tet repressor-tetracycline complex and regulation of antibiotic resistance. Science 264, 418–420. doi: 10.1126/science.8153629, 8153629

[ref19] Holmes D. E. Risso C. Smith J. A. Lovley D. R. (2012). Genome-scale analysis of anaerobic benzoate and phenol metabolism in the hyperthermophilic archaeon *Ferroglobus placidus*. ISME J. 6, 146–157. doi: 10.1038/ismej.2011.88, 21776029 PMC3246244

[ref20] Hu Y. F. Han X. Luo Y. Hjiang J. Jiang Y. Cao B. . (2024). All roads lead to Rome: cyclic di-GMP differentially regulates extracellular electron transfer in Geobacter biofilms. Innov. Life 2:100052. doi: 10.59717/j.xinn-life.2024.100052

[ref21] Inoue K. Qian X. Morgado L. Kim B. C. Mester T. Izallalen M. . (2010). Purification and characterization of OmcZ, an outer-surface, octaheme c-type cytochrome essential for optimal current production by *Geobacter sulfurreducens*. Appl. Environ. Microbiol. 76, 3999–4007. doi: 10.1128/AEM.00027-10, 20400562 PMC2893489

[ref22] Jalili P. Ala A. Nazari P. Jalili B. Ganji D. D. (2024). A comprehensive review of microbial fuel cells considering materials, methods, structures, and microorganisms. Heliyon 10:e25439. doi: 10.1016/j.heliyon.2024.e25439, 38371992 PMC10873675

[ref23] Jenal U. Reinders A. Lori C. (2017). Cyclic di-GMP: second messenger extraordinaire. Nat. Rev. Microbiol. 15, 271–284. doi: 10.1038/nrmicro.2016.190, 28163311

[ref24] Jones D. (1999). Protein secondary structure prediction based on position-specific scoring matrices. J. Mol. Biol. 292, 195–202. doi: 10.1006/jmbi.1999.3091, 10493868

[ref25] Jumper J. Evans R. Pritzel A. Green T. Figurnov M. Ronneberger O. . (2021). Highly accurate protein structure prediction with AlphaFold. Nature 596, 583–589. doi: 10.1038/s41586-021-03819-2, 34265844 PMC8371605

[ref26] Kellenberger C. A. Wilson S. C. Hickey S. F. Gonzalez T. L. Su Y. Hallberg Z. F. . (2015). GEMM-I riboswitches from Geobacter sense the bacterial second messenger cyclic AMP-GMP. Proc. Natl. Acad. Sci. USA 112, 5383–5388. doi: 10.1073/pnas.1419328112, 25848022 PMC4418906

[ref27] Kim B. C. Qian X. Leang C. Coppi M. V. Lovley D. R. (2006). Two putative c-type multiheme cytochromes required for the expression of OmcB, an outer membrane protein essential for optimal Fe(III) reduction in *Geobacter sulfurreducens*. J. Bacteriol. 188, 3138–3142. doi: 10.1128/JB.188.8.3138-3142.2006, 16585776 PMC1447008

[ref28] Leang C. Malvankar N. S. Franks A. E. Nevin K. P. Lovley D. R. (2013). Engineering *Geobacter sulfurreducens* to produce a highly cohesive conductive matrix with enhanced capacity for current production. Energy Environ. Sci. 6, 1901–1908. doi: 10.1039/c3ee40441b

[ref29] Levar C. Rollefson J. Bond D. (2013). “Energetic and molecular constraints on the mechanism of environmental Fe(III) reduction by Geobacter,” in Microbial Metal Respiration, ed. Kappler J. G. A. (Berlin: Springer-Verlag), 29–48.

[ref30] Lewis K. A. Vermilyea D. M. Webster S. S. Geiger C. J. De Anda J. Wong G. C. L. . (2022). Nonmotile subpopulations of *Pseudomonas aeruginosa* repress Flagellar motility in motile cells through a type IV pilus- and Pel-dependent mechanism. J. Bacteriol. 204:e0052821. doi: 10.1128/jb.00528-21, 35377166 PMC9112919

[ref9001] Lin W. C. Coppi M. V. Lovley D. R. (2004).Geobacter sulfurreducens Can Grow with Oxygen as a Terminal Electron Acceptor. Appl Environ Microbiol 70. doi: 10.1128/AEM.70.4.2525-2528.2004 PMC383164 15066854

[ref31] Liu X. Y. Holmes D. E. Walker D. J. F. Li Y. Meier D. Pinches S. . (2022). Cytochrome OmcS is not essential for extracellular Electron transport via conductive pili in *Geobacter sulfurreducens* strain KN400. Appl. Environ. Microbiol. 88:e0162221. doi: 10.1128/AEM.01622-21, 34669448 PMC8752155

[ref32] Liu F. Rotaru A. Shrestha P. Malvankar N. Nevin K. Lovley D. (2012). Promoting direct Interspecies electron transfer with Activated carbon. Energy Environ. Sci. 5:8982. doi: 10.1039/c2ee22459c

[ref33] Liu X. Tremblay P. L. Malvankar N. S. Nevin K. P. Lovley D. R. Vargas M. (2014). A *Geobacter sulfurreducens* strain expressing *pseudomonas aeruginosa* type IV pili localizes OmcS on pili but is deficient in Fe(III) oxide reduction and current production. Appl. Environ. Microbiol. 80, 1219–1224. doi: 10.1128/AEM.02938-13, 24296506 PMC3911229

[ref34] Liu X. Walker D. J. F. Nonnenmann S. S. Sun D. Lovley D. R. (2021). Direct observation of electrically conductive pili emanating from *Geobacter sulfurreducens*. mBio 12:e0220921. doi: 10.1128/mBio.02209-21, 34465020 PMC8406130

[ref35] Logan B. E. Rossi R. Ragab A. Saikaly P. E. (2019). Electroactive microorganisms in bioelectrochemical systems. Nat. Rev. Microbiol. 17, 307–319. doi: 10.1038/s41579-019-0173-x 30846876

[ref36] Lovley D. (2004). “Potential role of dissimilatory iron reduction in the early evolution of microbial respiration,” in Origins, Evolution and Biodiversity of Microbial Life, (Dordrecht: Kluwer Academic Publishers), 301–313.

[ref37] Lovley D. R. (2006). Bug juice: harvesting electricity with microorganisms. Nat. Rev. Microbiol. 4, 497–508. doi: 10.1038/nrmicro1442, 16778836

[ref38] Lovley D. (2011). Live wires: direct extracellular electron exchange for bioenergy and the bioremediation of energy-related contamination. Energy Environ. Sci. 4, 4896–4906. doi: 10.1039/c1ee02229f

[ref39] Lovley D. R. (2012). Electromicrobiology. Annu. Rev. Microbiol. 66, 391–409. doi: 10.1146/annurev-micro-092611-150104 22746334

[ref40] Lovley D. R. (2025). Commentary: Electron transport across the cell envelope via multiheme c-type cytochromes in *Geobacter sulfurreducens*. Front. Chem. 13:1674350. doi: 10.3389/fchem.2025.1674350, 41189673 PMC12580347

[ref41] Lovley D. (2026). Extracellular Electron transfer: from early life to modern biogeochemistry and applications. Adv. Microb. Physiol. 88, 1–125. doi: 10.1016/bs.ampbs.2026.02.004 42203390

[ref42] Lovley D. Greening R. Ferry J. G. (1984). Rapidly growing rumen methanogenic organism that synthesizes coenzyme M and has a high affinity for formate. Appl. Environ. Microbiol. 48, 81–87. doi: 10.1128/aem.48.1.81-87.1984, 6433795 PMC240316

[ref43] Lovley D. R. Holmes D. E. (2022). Electromicrobiology: the ecophysiology of phylogenetically diverse electroactive microorganisms. Nat. Rev. Microbiol. 20, 5–19. doi: 10.1038/s41579-021-00597-6, 34316046

[ref44] Lovley D. R. Phillips E. J. (1987). Rapid assay for microbially reducible ferric iron in aquatic sediments. Appl. Environ. Microbiol. 53, 1536–1540. doi: 10.1128/aem.53.7.1536-1540.1987, 16347384 PMC203906

[ref45] Lovley D. R. Phillips E. J. P. (1988). Novel mode of microbial energy-metabolism - organic-carbon oxidation coupled to dissimilatory reduction of Iron or manganese. Appl. Environ. Microbiol. 54, 1472–1480. doi: 10.1128/aem.54.6.1472-1480.1988, 16347658 PMC202682

[ref46] Maddocks S. E. Oyston P. C. F. (2008). Structure and function of the LysR-type transcriptional regulator (LTTR) family proteins. Microbiology (Reading) 154, 3609–3623. doi: 10.1099/mic.0.2008/022772-0, 19047729

[ref47] Malvankar N. S. Tuominen M. T. Lovley D. R. (2012a). Biofilm conductivity is a decisive variable for high-current-density microbial fuel cells. Energy Environ. Sci. 5, 5790–5797. doi: 10.1039/c2ee03388g

[ref48] Malvankar N. S. Tuominen M. T. Lovley D. R. (2012b). Lack of cytochrome involvement in long-range electron transport through conductive biofilms and nanowires of *Geobacter sulfurreducens*. Energy Environ. Sci. 5, 8651–8659. doi: 10.1039/c2ee22330a

[ref49] Malvankar N. S. Vargas M. Nevin K. P. Franks A. E. Leang C. Kim B. C. . (2011). Tunable metallic-like conductivity in microbial nanowire networks. Nat. Nanotechnol. 6, 573–579. doi: 10.1038/nnano.2011.119 21822253

[ref50] Mccourt J. O'halloran D. P. Mccarthy H. O'gara J. P. Geoghegan J. A. (2014). Fibronectin-binding proteins are required for biofilm formation by community-associated methicillin-resistant *Staphylococcus aureus* strain LAC. FEMS Microbiol. Lett. 353, 157–164. doi: 10.1111/1574-6968.12424, 24628034

[ref51] Nevin K. P. Kim B. C. Glaven R. H. Johnson J. P. Woodard T. L. Methe B. A. . (2009). Anode biofilm transcriptomics reveals outer surface components essential for high density current production in *Geobacter sulfurreducens* fuel cells. PLoS One 4:e5628. doi: 10.1371/journal.pone.0005628, 19461962 PMC2680965

[ref52] Otero F. J. Chan C. H. Bond D. R. (2018). Identification of different putative outer membrane electron conduits necessary for Fe(III) citrate, Fe(III) oxide, Mn(IV) oxide, or electrode reduction by *Geobacter sulfurreducens*. J. Bacteriol. 200:e00347-18. doi: 10.1128/JB.00347-18, 30038047 PMC6148476

[ref53] Peres C. M. Harwood C. S. (2006). BadM is a transcriptional repressor and one of three regulators that control benzoyl coenzyme a reductase gene expression in *Rhodopseudomonas palustris*. J. Bacteriol. 188, 8662–8665. doi: 10.1128/JB.01312-06, 17041049 PMC1698244

[ref54] Pettersen E. F. Goddard T. D. Huang C. C. Meng E. C. Couch G. S. Croll T. I. . (2021). UCSF ChimeraX: structure visualization for researchers, educators, and developers. Protein Sci. 30, 70–82. doi: 10.1002/pro.3943, 32881101 PMC7737788

[ref55] R Core Team (2021). R: A Language and Environment for Statistical Computing. Vienna: R Foundation for Statistical Computing.

[ref56] Rabaey K. Boon N. Hofte M. Verstraete W. (2005). Microbial phenazine production enhances electron transfer in biofuel cells. Environ. Sci. Technol. 39, 3401–3408. doi: 10.1021/es048563o, 15926596

[ref57] Reguera G. Nevin K. P. Nicoll J. S. Covalla S. F. Woodard T. L. Lovley D. R. (2006). Biofilm and nanowire production leads to increased current in *Geobacter sulfurreducens* fuel cells. Appl. Environ. Microbiol. 72, 7345–7348. doi: 10.1128/AEM.01444-06, 16936064 PMC1636155

[ref58] Ringeisen B. Henderson E. Wu P. Pietron J. Ray R. I. Little B. . (2006). High power density from a miniature microbial fuel cell using *Shewanella oneidensis* DSP10. Environ. Sci. Technol. 40, 2629–2634. doi: 10.1021/es052254w 16683602

[ref9002] Risso C. Methé B. A., Elifantz H. Holmes D. E. Lovley D. R. (2008). Highly conserved genes in Geobacter species with expression patterns indicative of acetate limitation. Microbiol 154. doi: 10.1099/mic.0.2008/017244-0 18757793

[ref59] Rotaru A. E. Woodard T. L. Nevin K. P. Lovley D. R. (2015). Link between capacity for current production and syntrophic growth in Geobacter species. Front. Microbiol. 6:744. doi: 10.3389/fmicb.2015.00744, 26284037 PMC4523033

[ref60] Schleif R. (2010). AraC protein, regulation of the l-arabinose operon in Escherichia coli, and the light switch mechanism of AraC action. FEMS Microbiol. Rev. 34, 779–796. doi: 10.1111/j.1574-6976.2010.00226.x, 20491933

[ref61] Schwarz I. A. Alsaqri B. Lekbach Y. Henry K. Gorman S. Woodard T. . (2024). Lack of physiological evidence for cytochrome filaments functioning as conduits for extracellular electron transfer. mBio 15:e0069024. doi: 10.1128/mbio.00690-24, 38717196 PMC11077965

[ref62] Seeman T. (2015). Snippy: Fast Bacterial Variant Calling from NGS Reads. Available online at: https://github.com/tseemann/snippy (Accessed September 28, 2025).

[ref63] Semenec L. Vergara I. A. Laloo A. E. Petrovski S. Bond P. L. Franks A. E. (2020). Adaptive evolution of *Geobacter sulfurreducens* in Coculture with *Pseudomonas aeruginosa*. mBio 11:11. doi: 10.1128/mBio.02875-19, 32265334 PMC7157779

[ref64] Shen C. Salazar-Morales A. I. Jung W. Erwin J. Gu Y. Coelho A. . (2025). A widespread and ancient bacterial machinery assembles cytochrome OmcS nanowires essential for extracellular electron transfer. Cell Chem. Biol. 32, 239–254.e7. doi: 10.1016/j.chembiol.2024.12.013, 39818215 PMC11845295

[ref65] Shrestha P. Nevin K. Shrestha M. Lovley D. (2013). When is a microbial culture “pure”? Persistent cryptic contaminant escapes detection even with deep genome sequencing. mBio 4:e00591-12. doi: 10.1128/mBio.00591-12, 23481604 PMC3604776

[ref66] Smith J. A. Tremblay P. L. Shrestha P. M. Snoeyenbos-West O. L. Franks A. E. Nevin K. P. . (2014). Going wireless: Fe(III) oxide reduction without pili by *Geobacter sulfurreducens* strain JS-1. Appl. Environ. Microbiol. 80, 4331–4340. doi: 10.1128/AEM.01122-14, 24814783 PMC4068678

[ref67] Steidl R. J. Lampa-Pastirk S. Reguera G. (2016). Mechanistic stratification in electroactive biofilms of *Geobacter sulfurreducens* mediated by pilus nanowires. Nat. Commun. 7:12217. doi: 10.1038/ncomms12217, 27481214 PMC4974642

[ref68] Tabari M. Z. Hochbaum A. I. (2025). Electron transport across the cell envelope via multiheme c-type cytochromes in *Geobacter sulfurreducens*. Front. Chem. 13:1621274. doi: 10.3389/fchem.2025.1621274, 40740311 PMC12307469

[ref69] Tan Y. Adhikari R. Y. Malvankar N. S. Ward J. E. Woodard T. L. Nevin K. P. . (2017). Expressing the *Geobacter metallireducens* PilA in *Geobacter sulfurreducens* yields pili with exceptional conductivity. mBio 8:e02203-16. doi: 10.1128/mBio.02203-16, 28096491 PMC5241403

[ref70] Tan Z. S. Chan C. H. Maleska M. Jara B. B. Lohman B. K. Ricks N. J. . (2022). The signaling pathway that cGAMP riboswitches found: analysis and application of riboswitches to study cGAMP signaling in. Int. J. Mol. Sci. 23:1183. doi: 10.3390/ijms23031183, 35163114 PMC8835794

[ref71] Tremblay P. L. Summers Z. M. Glaven R. H. Nevin K. P. Zengler K. Barrett C. L. . (2011). A c-type cytochrome and a transcriptional regulator responsible for enhanced extracellular electron transfer in revealed by adaptive evolution. Environ. Microbiol. 13, 13–23. doi: 10.1111/j.1462-2920.2010.02302.x, 20636372

[ref72] Ueki T. Leang C. Inoue K. Lovley D. R. (2012). Identification of multicomponent histidine-aspartate Phosphorelay system controlling Flagellar and motility gene expression in Geobacter species. J. Biol. Chem. 287, 10958–10966. doi: 10.1074/jbc.M112.345041, 22362768 PMC3322880

[ref73] Vargas M. Malvankar N. S. Tremblay P. L. Leang C. Smith J. A. Patel P. . (2013). Aromatic amino acids required for pili conductivity and long-range extracellular Electron transport in *Geobacter sulfurreducens*. mBio 4, e00105–e00113. doi: 10.1128/mBio.00105-13, 23481602 PMC3604773

[ref74] Wang F. Mustafa K. Suciu V. Joshi K. Chan C. Choi S.-C. . (2022). Cryo-EM structure of an extracellular Geobacter OmcE cytochrome filament reveals tetrahaem packing. Nat. Microbiol. 7, 1291–1300. doi: 10.1038/s41564-022-01159-z, 35798889 PMC9357133

[ref75] Yi H. N. Nevin K. P. Kim B. C. Franks A. E. Klimes A. Tender L. M. . (2009). Selection of a variant of with enhanced capacity for current production in microbial fuel cells. Biosens. Bioelectron. 24, 3498–3503. doi: 10.1016/j.bios.2009.05.004, 19487117

[ref76] Zacharoff L. A. Morrone D. J. Bond D. R. (2017). Extracellular Multiheme cytochrome PgcA facilitates respiration to Fe(III) oxides but not electrodes. Front. Microbiol. 8:2481. doi: 10.3389/fmicb.2017.02481, 29312190 PMC5732950

[ref77] Zhang Y. Skolnick J. (2004). Scoring function for automated assessment of protein structure template quality. Proteins 57, 702–710. doi: 10.1002/prot.20264, 15476259

[ref78] Zhu Q. Bai X. Li Q. Zhang M. Hu G. Pan K. . (2023). PcaR, a GntR/FadR family transcriptional repressor controls the transcription of Phenazine-1-carboxylic acid 1,2-dioxygenase gene cluster in *Sphingomonas histidinilytica* DS-9. Appl. Environ. Microbiol. 89:e0212122. doi: 10.1128/aem.02121-22, 37191535 PMC10304782

