# Supplementary material for: Evolution for enhanced extracellular electron transfer in Geobacter sulfurreducens over seventeen years of continuous current generation
Source: Front Microbiol. 2026 May 8;17:1771963. doi: 10.3389/fmicb.2026.1771963 (PMC13194489; doi:10.3389/fmicb.2026.1771963)
Supplement: Supplementary file 1 [file Supplementary_file_1.zip › Supplementary Figure 3.PPTX]

## Slide 1
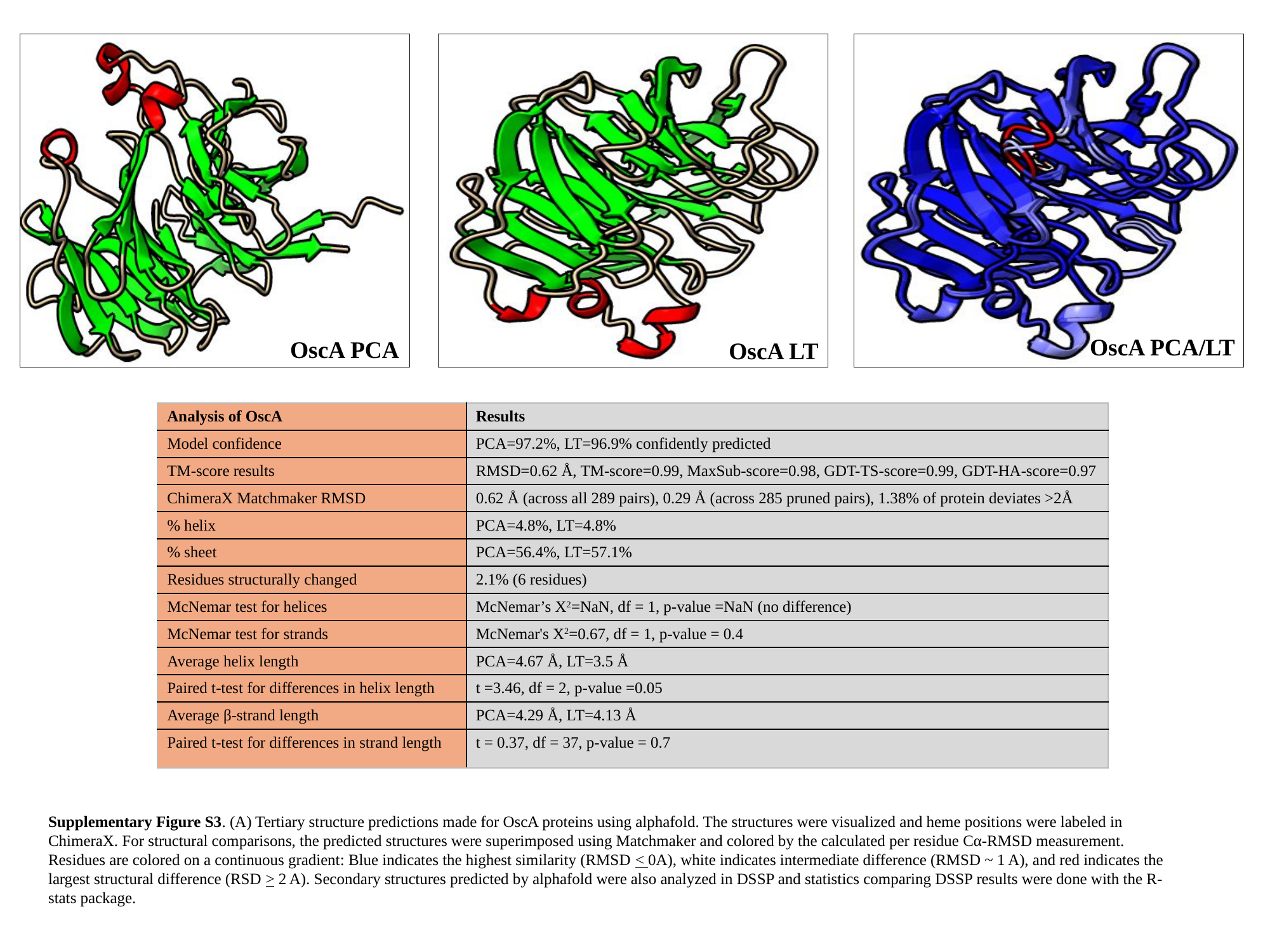

OscA PCA/LT
OscA PCA
OscA LT
| Analysis of OscA | Results |
| --- | --- |
| Model confidence | PCA=97.2%, LT=96.9% confidently predicted |
| TM-score results | RMSD=0.62 Å, TM-score=0.99, MaxSub-score=0.98, GDT-TS-score=0.99, GDT-HA-score=0.97 |
| ChimeraX Matchmaker RMSD | 0.62 Å (across all 289 pairs), 0.29 Å (across 285 pruned pairs), 1.38% of protein deviates >2Å |
| % helix | PCA=4.8%, LT=4.8% |
| % sheet | PCA=56.4%, LT=57.1% |
| Residues structurally changed | 2.1% (6 residues) |
| McNemar test for helices | McNemar’s X2=NaN, df = 1, p-value =NaN (no difference) |
| McNemar test for strands | McNemar's X2=0.67, df = 1, p-value = 0.4 |
| Average helix length | PCA=4.67 Å, LT=3.5 Å |
| Paired t-test for differences in helix length | t =3.46, df = 2, p-value =0.05 |
| Average β-strand length | PCA=4.29 Å, LT=4.13 Å |
| Paired t-test for differences in strand length | t = 0.37, df = 37, p-value = 0.7 |
Supplementary Figure S3. (A) Tertiary structure predictions made for OscA proteins using alphafold. The structures were visualized and heme positions were labeled in ChimeraX. For structural comparisons, the predicted structures were superimposed using Matchmaker and colored by the calculated per residue Cα-RMSD measurement. Residues are colored on a continuous gradient: Blue indicates the highest similarity (RMSD < 0A), white indicates intermediate difference (RMSD ~ 1 A), and red indicates the largest structural difference (RSD > 2 A). Secondary structures predicted by alphafold were also analyzed in DSSP and statistics comparing DSSP results were done with the R-stats package.

## Slide 2
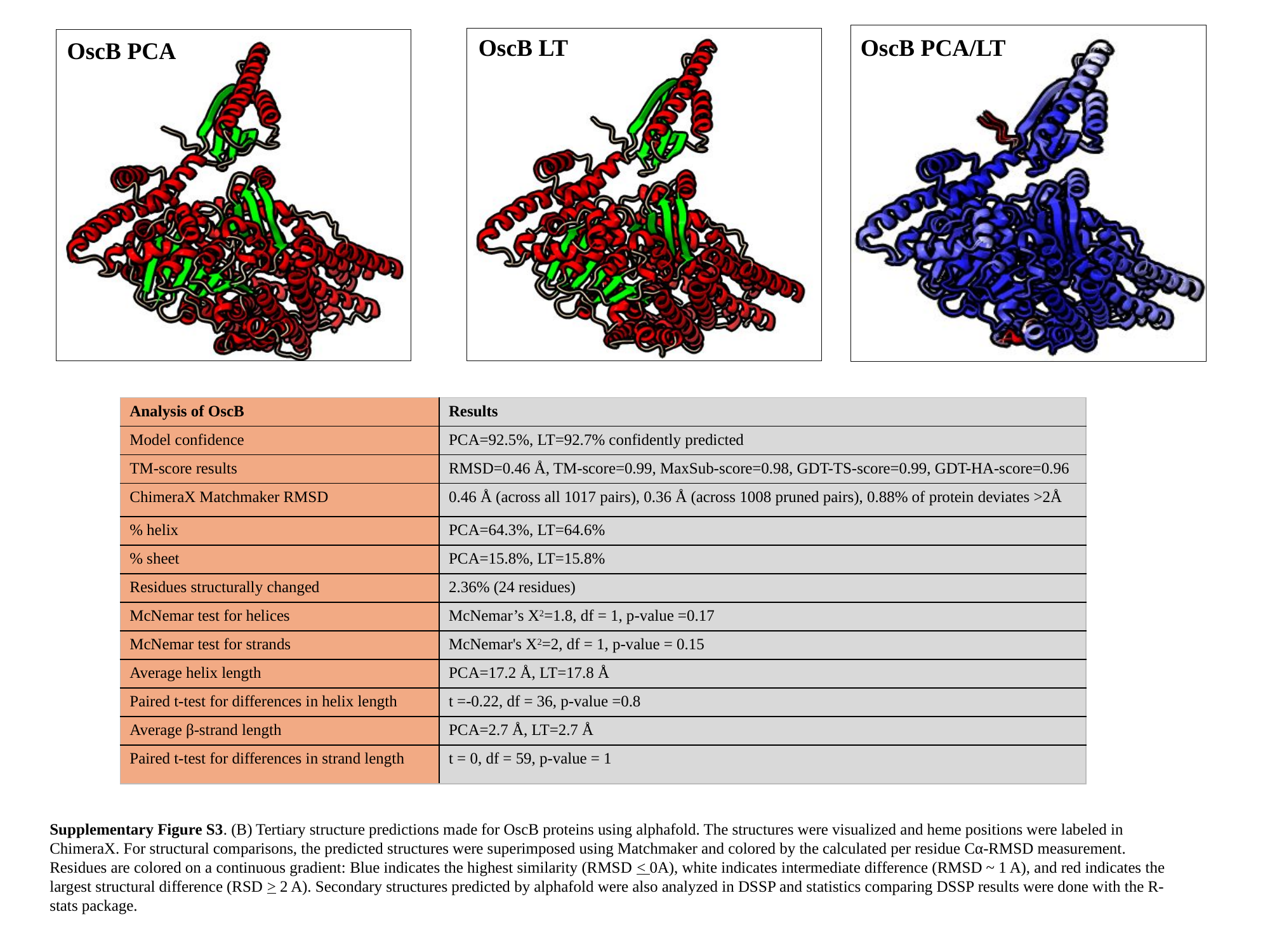

OscB LT
OscB PCA/LT
OscB PCA
| Analysis of OscB | Results |
| --- | --- |
| Model confidence | PCA=92.5%, LT=92.7% confidently predicted |
| TM-score results | RMSD=0.46 Å, TM-score=0.99, MaxSub-score=0.98, GDT-TS-score=0.99, GDT-HA-score=0.96 |
| ChimeraX Matchmaker RMSD | 0.46 Å (across all 1017 pairs), 0.36 Å (across 1008 pruned pairs), 0.88% of protein deviates >2Å |
| % helix | PCA=64.3%, LT=64.6% |
| % sheet | PCA=15.8%, LT=15.8% |
| Residues structurally changed | 2.36% (24 residues) |
| McNemar test for helices | McNemar’s X2=1.8, df = 1, p-value =0.17 |
| McNemar test for strands | McNemar's X2=2, df = 1, p-value = 0.15 |
| Average helix length | PCA=17.2 Å, LT=17.8 Å |
| Paired t-test for differences in helix length | t =-0.22, df = 36, p-value =0.8 |
| Average β-strand length | PCA=2.7 Å, LT=2.7 Å |
| Paired t-test for differences in strand length | t = 0, df = 59, p-value = 1 |
Supplementary Figure S3. (B) Tertiary structure predictions made for OscB proteins using alphafold. The structures were visualized and heme positions were labeled in ChimeraX. For structural comparisons, the predicted structures were superimposed using Matchmaker and colored by the calculated per residue Cα-RMSD measurement. Residues are colored on a continuous gradient: Blue indicates the highest similarity (RMSD < 0A), white indicates intermediate difference (RMSD ~ 1 A), and red indicates the largest structural difference (RSD > 2 A). Secondary structures predicted by alphafold were also analyzed in DSSP and statistics comparing DSSP results were done with the R-stats package.

## Slide 3
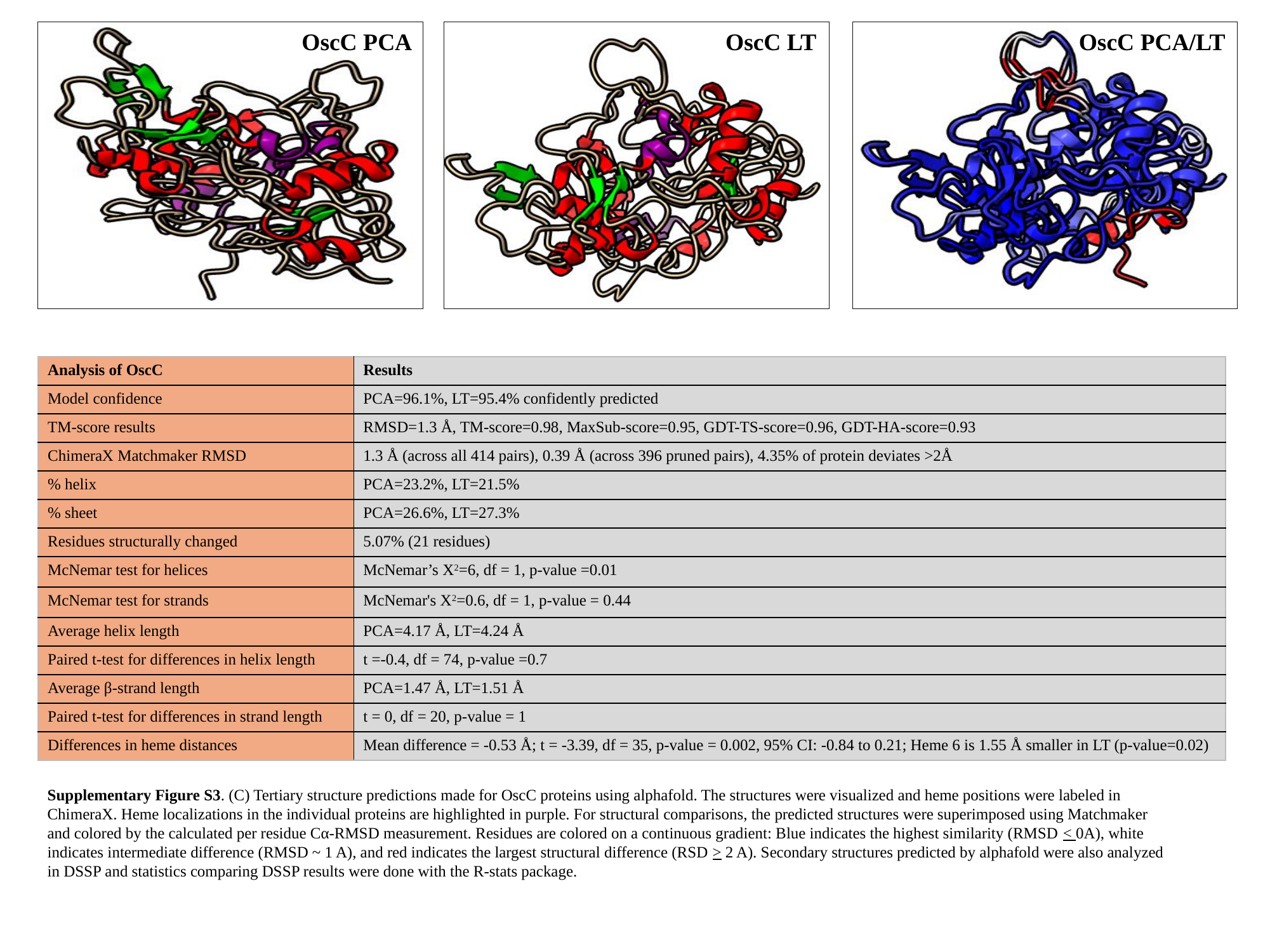

OscC PCA
OscC LT
OscC PCA/LT
| Analysis of OscC | Results |
| --- | --- |
| Model confidence | PCA=96.1%, LT=95.4% confidently predicted |
| TM-score results | RMSD=1.3 Å, TM-score=0.98, MaxSub-score=0.95, GDT-TS-score=0.96, GDT-HA-score=0.93 |
| ChimeraX Matchmaker RMSD | 1.3 Å (across all 414 pairs), 0.39 Å (across 396 pruned pairs), 4.35% of protein deviates >2Å |
| % helix | PCA=23.2%, LT=21.5% |
| % sheet | PCA=26.6%, LT=27.3% |
| Residues structurally changed | 5.07% (21 residues) |
| McNemar test for helices | McNemar’s X2=6, df = 1, p-value =0.01 |
| McNemar test for strands | McNemar's X2=0.6, df = 1, p-value = 0.44 |
| Average helix length | PCA=4.17 Å, LT=4.24 Å |
| Paired t-test for differences in helix length | t =-0.4, df = 74, p-value =0.7 |
| Average β-strand length | PCA=1.47 Å, LT=1.51 Å |
| Paired t-test for differences in strand length | t = 0, df = 20, p-value = 1 |
| Differences in heme distances | Mean difference = -0.53 Å; t = -3.39, df = 35, p-value = 0.002, 95% CI: -0.84 to 0.21; Heme 6 is 1.55 Å smaller in LT (p-value=0.02) |
Supplementary Figure S3. (C) Tertiary structure predictions made for OscC proteins using alphafold. The structures were visualized and heme positions were labeled in ChimeraX. Heme localizations in the individual proteins are highlighted in purple. For structural comparisons, the predicted structures were superimposed using Matchmaker and colored by the calculated per residue Cα-RMSD measurement. Residues are colored on a continuous gradient: Blue indicates the highest similarity (RMSD < 0A), white indicates intermediate difference (RMSD ~ 1 A), and red indicates the largest structural difference (RSD > 2 A). Secondary structures predicted by alphafold were also analyzed in DSSP and statistics comparing DSSP results were done with the R-stats package.

## Slide 4
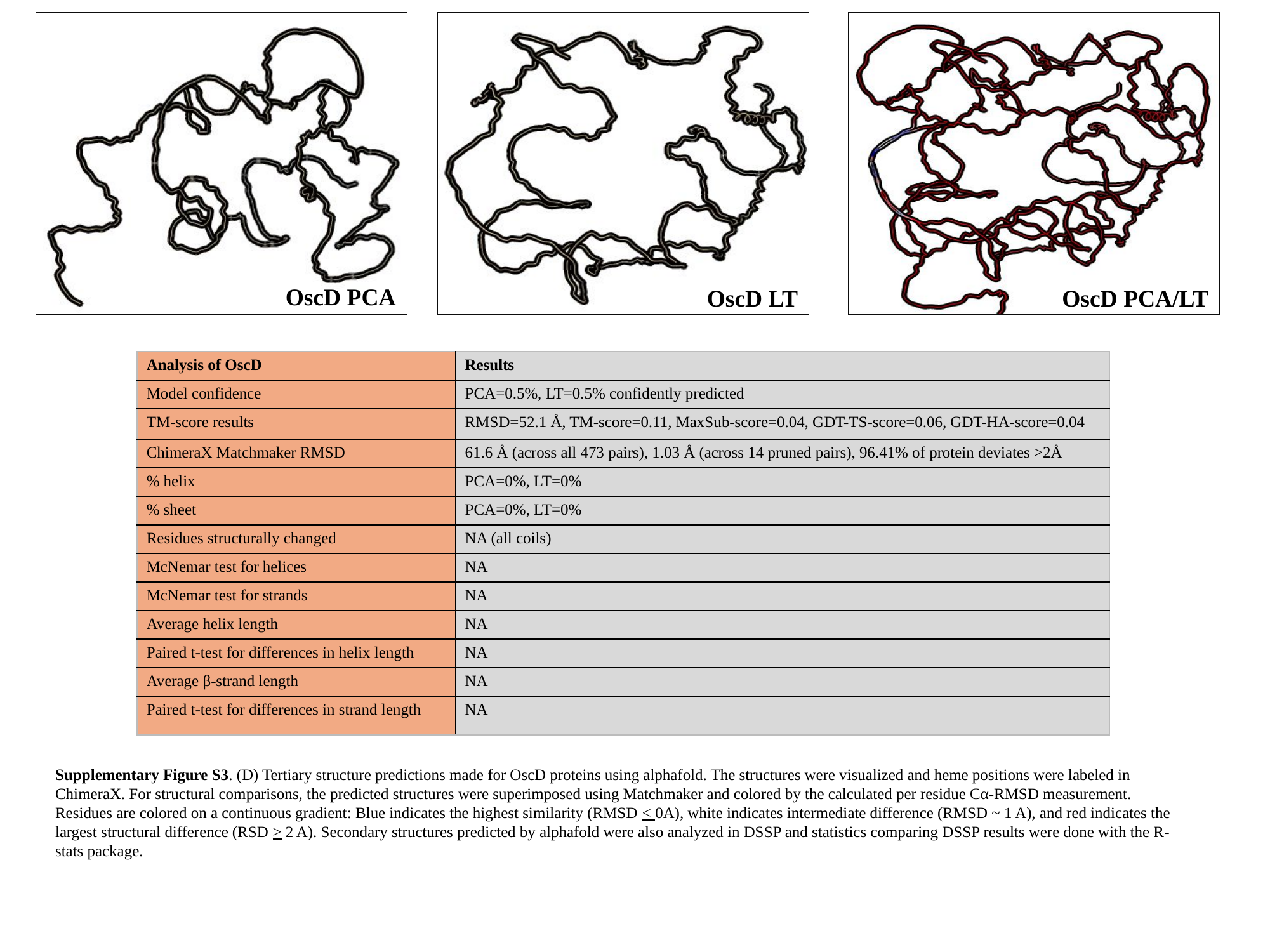

OscD PCA
OscD LT
OscD PCA/LT
| Analysis of OscD | Results |
| --- | --- |
| Model confidence | PCA=0.5%, LT=0.5% confidently predicted |
| TM-score results | RMSD=52.1 Å, TM-score=0.11, MaxSub-score=0.04, GDT-TS-score=0.06, GDT-HA-score=0.04 |
| ChimeraX Matchmaker RMSD | 61.6 Å (across all 473 pairs), 1.03 Å (across 14 pruned pairs), 96.41% of protein deviates >2Å |
| % helix | PCA=0%, LT=0% |
| % sheet | PCA=0%, LT=0% |
| Residues structurally changed | NA (all coils) |
| McNemar test for helices | NA |
| McNemar test for strands | NA |
| Average helix length | NA |
| Paired t-test for differences in helix length | NA |
| Average β-strand length | NA |
| Paired t-test for differences in strand length | NA |
Supplementary Figure S3. (D) Tertiary structure predictions made for OscD proteins using alphafold. The structures were visualized and heme positions were labeled in ChimeraX. For structural comparisons, the predicted structures were superimposed using Matchmaker and colored by the calculated per residue Cα-RMSD measurement. Residues are colored on a continuous gradient: Blue indicates the highest similarity (RMSD < 0A), white indicates intermediate difference (RMSD ~ 1 A), and red indicates the largest structural difference (RSD > 2 A). Secondary structures predicted by alphafold were also analyzed in DSSP and statistics comparing DSSP results were done with the R-stats package.

## Slide 5
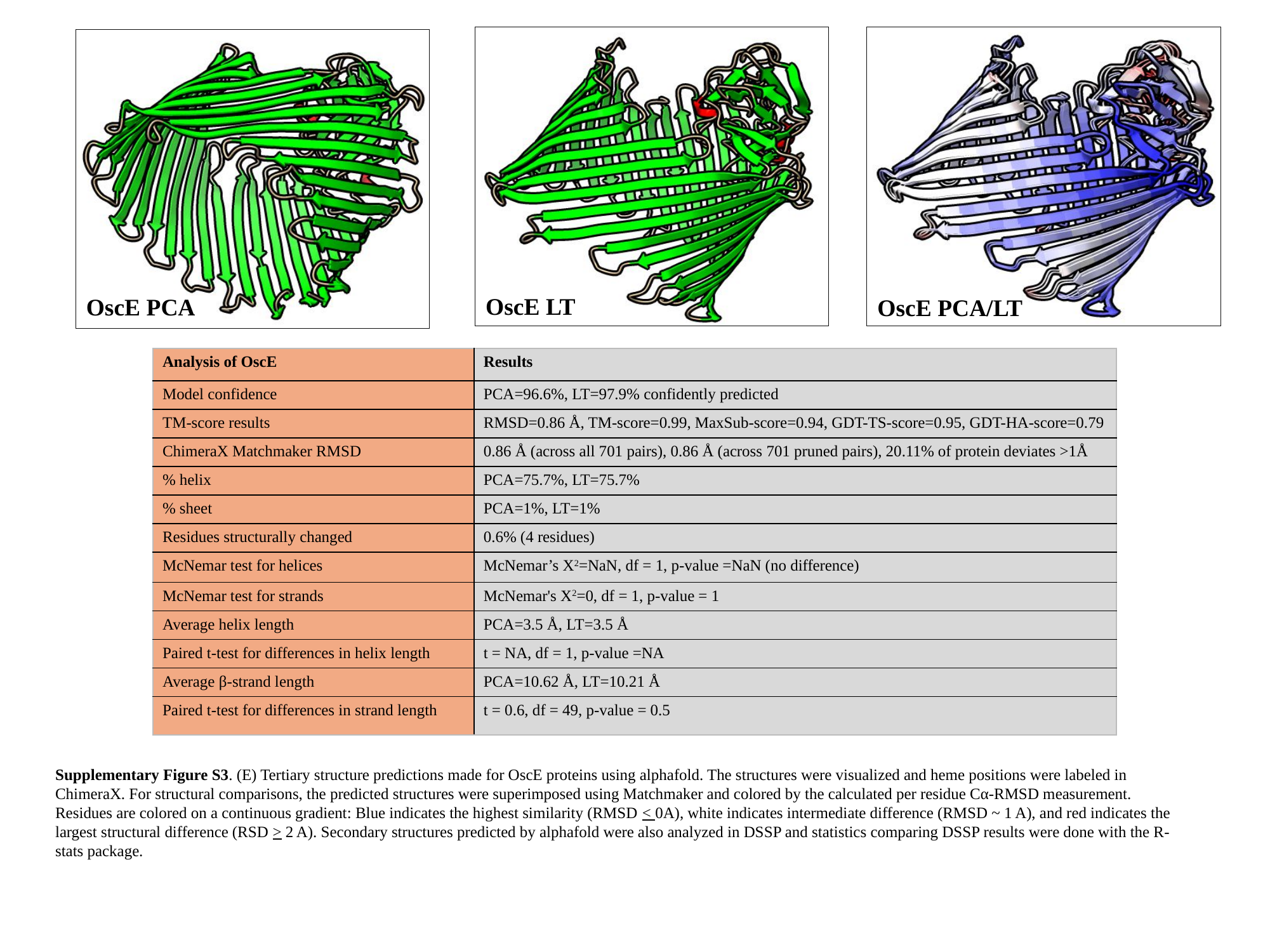

OscE LT
OscE PCA
OscE PCA/LT
| Analysis of OscE | Results |
| --- | --- |
| Model confidence | PCA=96.6%, LT=97.9% confidently predicted |
| TM-score results | RMSD=0.86 Å, TM-score=0.99, MaxSub-score=0.94, GDT-TS-score=0.95, GDT-HA-score=0.79 |
| ChimeraX Matchmaker RMSD | 0.86 Å (across all 701 pairs), 0.86 Å (across 701 pruned pairs), 20.11% of protein deviates >1Å |
| % helix | PCA=75.7%, LT=75.7% |
| % sheet | PCA=1%, LT=1% |
| Residues structurally changed | 0.6% (4 residues) |
| McNemar test for helices | McNemar’s X2=NaN, df = 1, p-value =NaN (no difference) |
| McNemar test for strands | McNemar's X2=0, df = 1, p-value = 1 |
| Average helix length | PCA=3.5 Å, LT=3.5 Å |
| Paired t-test for differences in helix length | t = NA, df = 1, p-value =NA |
| Average β-strand length | PCA=10.62 Å, LT=10.21 Å |
| Paired t-test for differences in strand length | t = 0.6, df = 49, p-value = 0.5 |
Supplementary Figure S3. (E) Tertiary structure predictions made for OscE proteins using alphafold. The structures were visualized and heme positions were labeled in ChimeraX. For structural comparisons, the predicted structures were superimposed using Matchmaker and colored by the calculated per residue Cα-RMSD measurement. Residues are colored on a continuous gradient: Blue indicates the highest similarity (RMSD < 0A), white indicates intermediate difference (RMSD ~ 1 A), and red indicates the largest structural difference (RSD > 2 A). Secondary structures predicted by alphafold were also analyzed in DSSP and statistics comparing DSSP results were done with the R-stats package.

## Slide 6
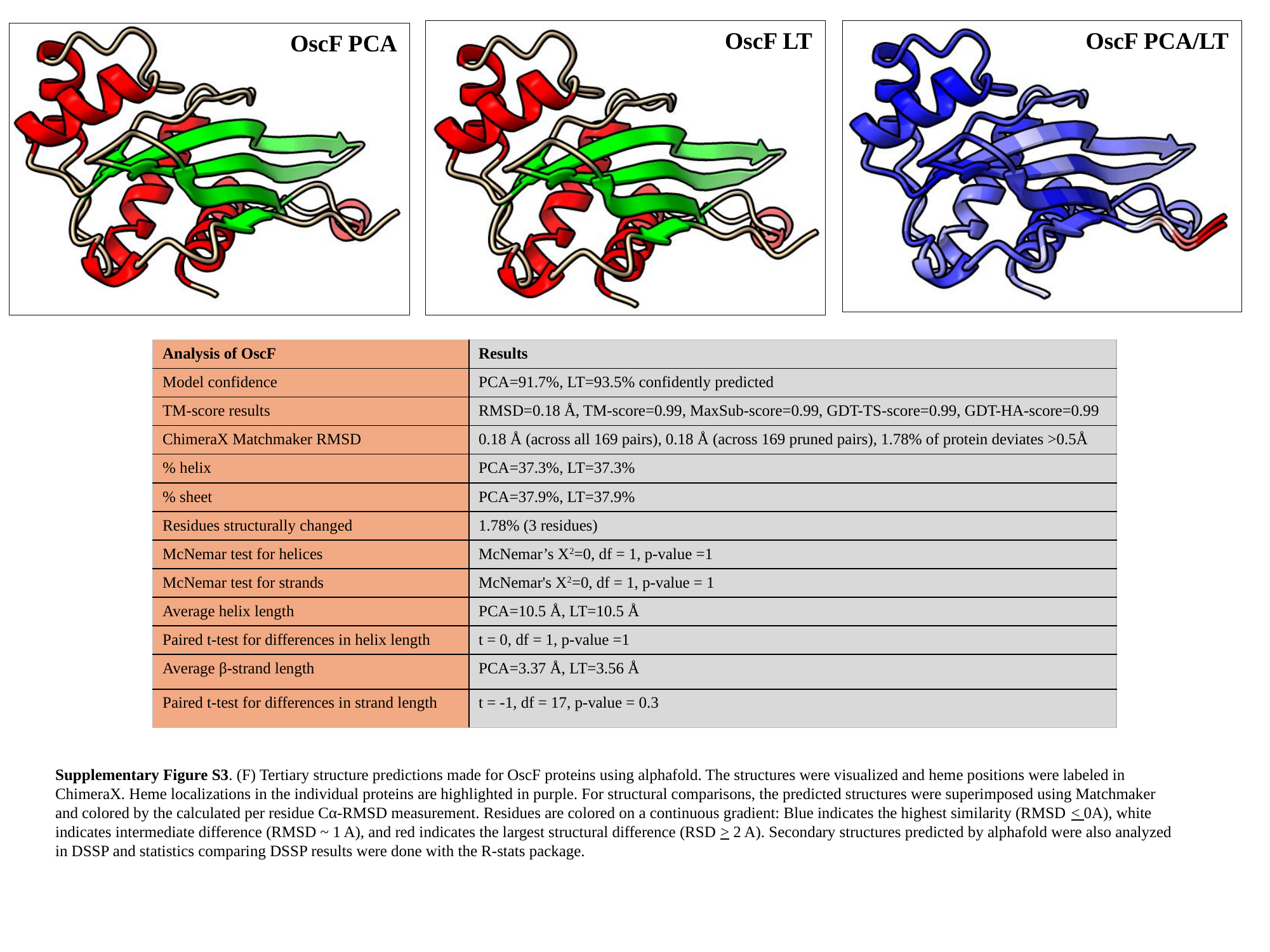

OscF LT
OscF PCA/LT
OscF PCA
| Analysis of OscF | Results |
| --- | --- |
| Model confidence | PCA=91.7%, LT=93.5% confidently predicted |
| TM-score results | RMSD=0.18 Å, TM-score=0.99, MaxSub-score=0.99, GDT-TS-score=0.99, GDT-HA-score=0.99 |
| ChimeraX Matchmaker RMSD | 0.18 Å (across all 169 pairs), 0.18 Å (across 169 pruned pairs), 1.78% of protein deviates >0.5Å |
| % helix | PCA=37.3%, LT=37.3% |
| % sheet | PCA=37.9%, LT=37.9% |
| Residues structurally changed | 1.78% (3 residues) |
| McNemar test for helices | McNemar’s X2=0, df = 1, p-value =1 |
| McNemar test for strands | McNemar's X2=0, df = 1, p-value = 1 |
| Average helix length | PCA=10.5 Å, LT=10.5 Å |
| Paired t-test for differences in helix length | t = 0, df = 1, p-value =1 |
| Average β-strand length | PCA=3.37 Å, LT=3.56 Å |
| Paired t-test for differences in strand length | t = -1, df = 17, p-value = 0.3 |
Supplementary Figure S3. (F) Tertiary structure predictions made for OscF proteins using alphafold. The structures were visualized and heme positions were labeled in ChimeraX. Heme localizations in the individual proteins are highlighted in purple. For structural comparisons, the predicted structures were superimposed using Matchmaker and colored by the calculated per residue Cα-RMSD measurement. Residues are colored on a continuous gradient: Blue indicates the highest similarity (RMSD < 0A), white indicates intermediate difference (RMSD ~ 1 A), and red indicates the largest structural difference (RSD > 2 A). Secondary structures predicted by alphafold were also analyzed in DSSP and statistics comparing DSSP results were done with the R-stats package.

## Slide 7
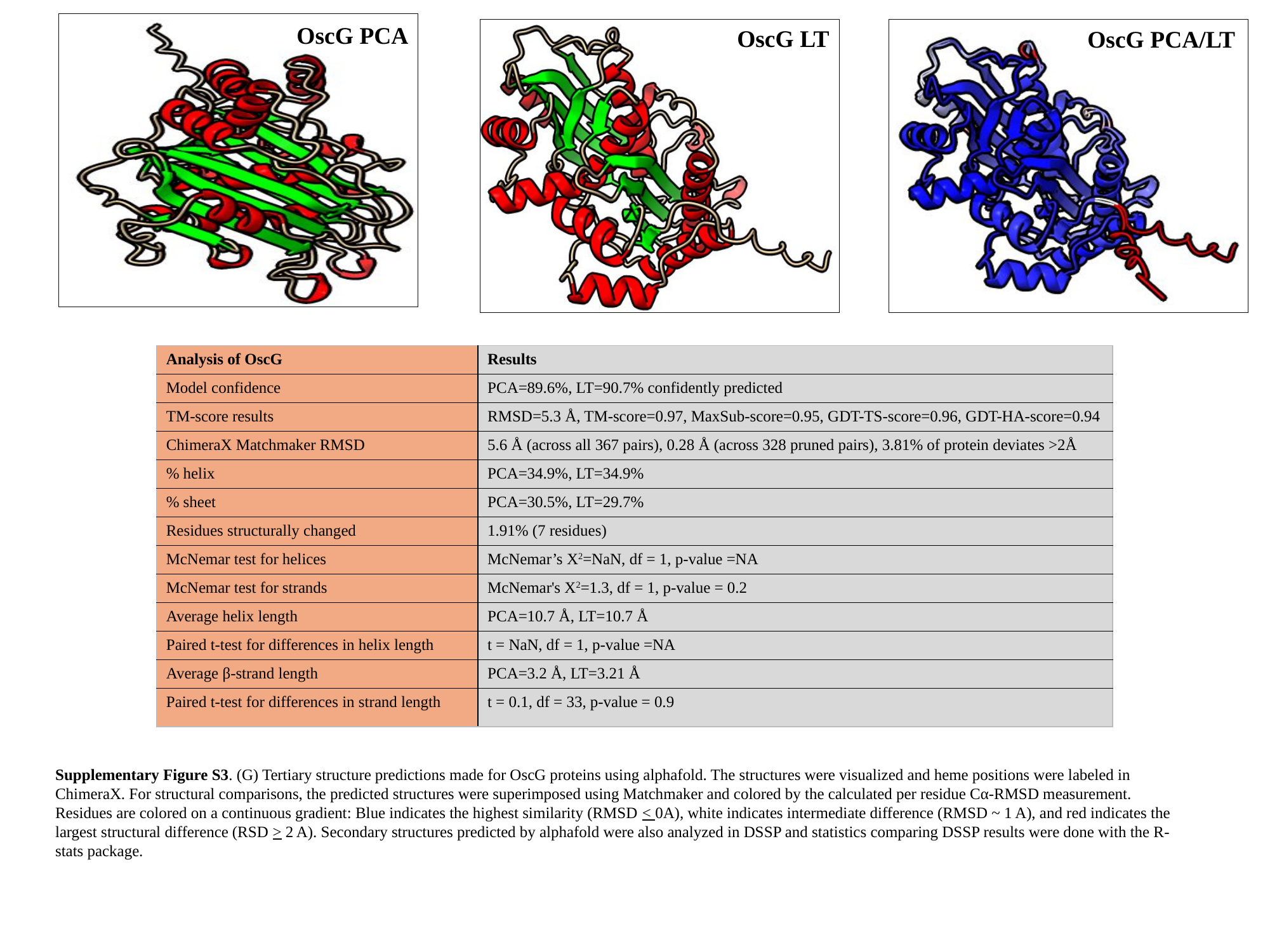

OscG PCA
OscG LT
OscG PCA/LT
| Analysis of OscG | Results |
| --- | --- |
| Model confidence | PCA=89.6%, LT=90.7% confidently predicted |
| TM-score results | RMSD=5.3 Å, TM-score=0.97, MaxSub-score=0.95, GDT-TS-score=0.96, GDT-HA-score=0.94 |
| ChimeraX Matchmaker RMSD | 5.6 Å (across all 367 pairs), 0.28 Å (across 328 pruned pairs), 3.81% of protein deviates >2Å |
| % helix | PCA=34.9%, LT=34.9% |
| % sheet | PCA=30.5%, LT=29.7% |
| Residues structurally changed | 1.91% (7 residues) |
| McNemar test for helices | McNemar’s X2=NaN, df = 1, p-value =NA |
| McNemar test for strands | McNemar's X2=1.3, df = 1, p-value = 0.2 |
| Average helix length | PCA=10.7 Å, LT=10.7 Å |
| Paired t-test for differences in helix length | t = NaN, df = 1, p-value =NA |
| Average β-strand length | PCA=3.2 Å, LT=3.21 Å |
| Paired t-test for differences in strand length | t = 0.1, df = 33, p-value = 0.9 |
Supplementary Figure S3. (G) Tertiary structure predictions made for OscG proteins using alphafold. The structures were visualized and heme positions were labeled in ChimeraX. For structural comparisons, the predicted structures were superimposed using Matchmaker and colored by the calculated per residue Cα-RMSD measurement. Residues are colored on a continuous gradient: Blue indicates the highest similarity (RMSD < 0A), white indicates intermediate difference (RMSD ~ 1 A), and red indicates the largest structural difference (RSD > 2 A). Secondary structures predicted by alphafold were also analyzed in DSSP and statistics comparing DSSP results were done with the R-stats package.

## Slide 8
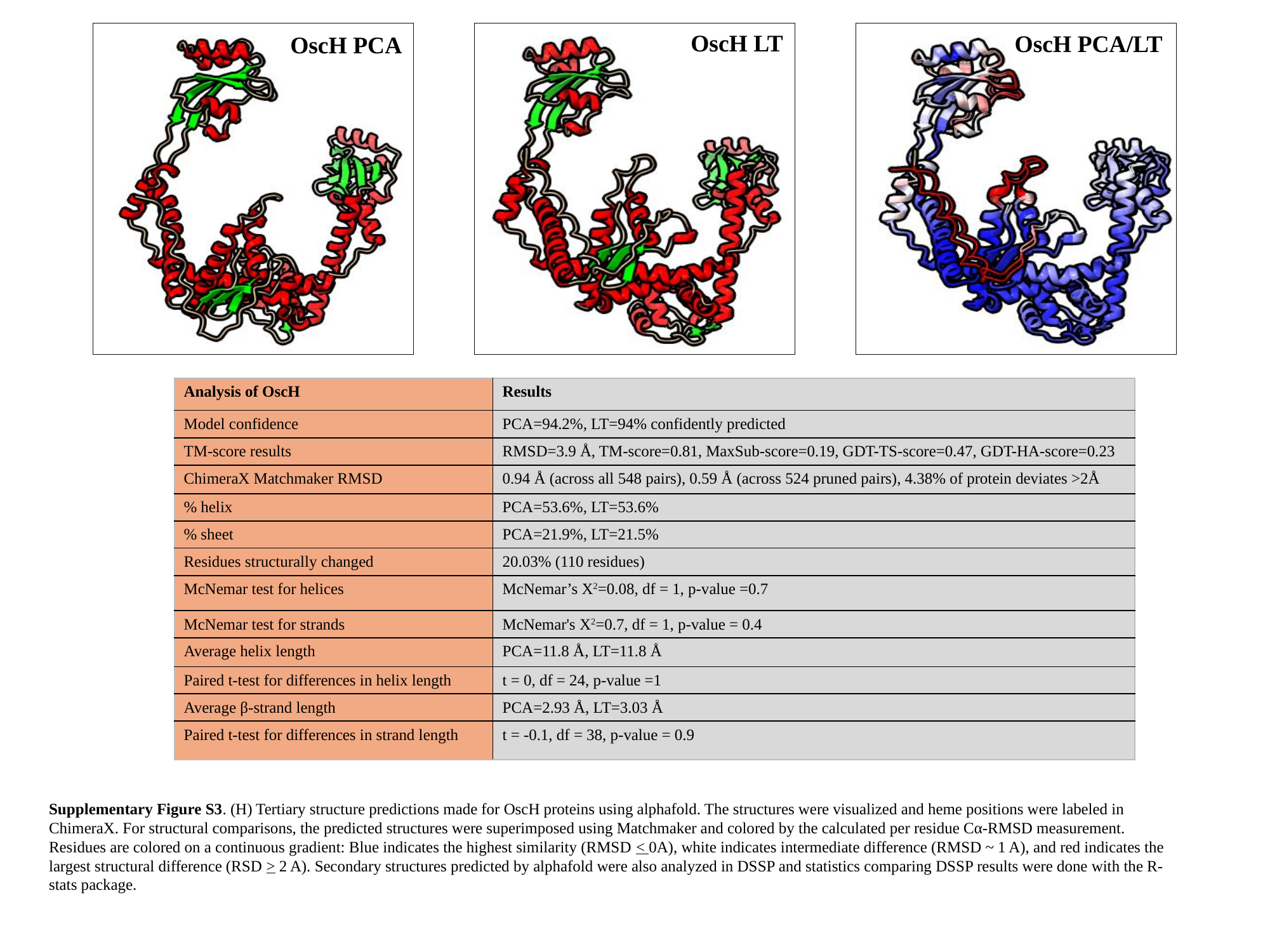

OscH LT
OscH PCA/LT
OscH PCA
| Analysis of OscH | Results |
| --- | --- |
| Model confidence | PCA=94.2%, LT=94% confidently predicted |
| TM-score results | RMSD=3.9 Å, TM-score=0.81, MaxSub-score=0.19, GDT-TS-score=0.47, GDT-HA-score=0.23 |
| ChimeraX Matchmaker RMSD | 0.94 Å (across all 548 pairs), 0.59 Å (across 524 pruned pairs), 4.38% of protein deviates >2Å |
| % helix | PCA=53.6%, LT=53.6% |
| % sheet | PCA=21.9%, LT=21.5% |
| Residues structurally changed | 20.03% (110 residues) |
| McNemar test for helices | McNemar’s X2=0.08, df = 1, p-value =0.7 |
| McNemar test for strands | McNemar's X2=0.7, df = 1, p-value = 0.4 |
| Average helix length | PCA=11.8 Å, LT=11.8 Å |
| Paired t-test for differences in helix length | t = 0, df = 24, p-value =1 |
| Average β-strand length | PCA=2.93 Å, LT=3.03 Å |
| Paired t-test for differences in strand length | t = -0.1, df = 38, p-value = 0.9 |
Supplementary Figure S3. (H) Tertiary structure predictions made for OscH proteins using alphafold. The structures were visualized and heme positions were labeled in ChimeraX. For structural comparisons, the predicted structures were superimposed using Matchmaker and colored by the calculated per residue Cα-RMSD measurement. Residues are colored on a continuous gradient: Blue indicates the highest similarity (RMSD < 0A), white indicates intermediate difference (RMSD ~ 1 A), and red indicates the largest structural difference (RSD > 2 A). Secondary structures predicted by alphafold were also analyzed in DSSP and statistics comparing DSSP results were done with the R-stats package.

## Slide 9
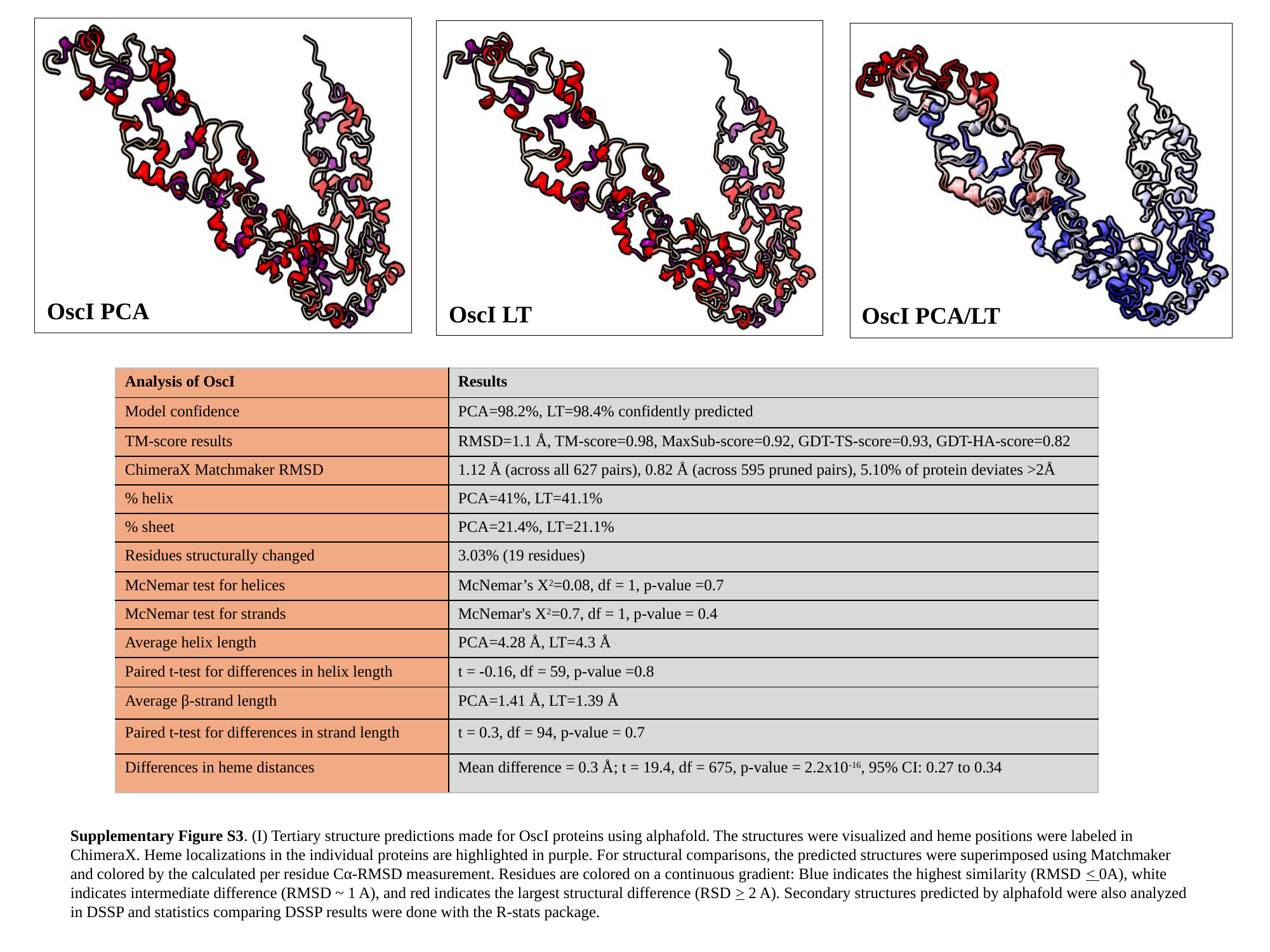

OscI PCA
OscI LT
OscI PCA/LT
| Analysis of OscI | Results |
| --- | --- |
| Model confidence | PCA=98.2%, LT=98.4% confidently predicted |
| TM-score results | RMSD=1.1 Å, TM-score=0.98, MaxSub-score=0.92, GDT-TS-score=0.93, GDT-HA-score=0.82 |
| ChimeraX Matchmaker RMSD | 1.12 Å (across all 627 pairs), 0.82 Å (across 595 pruned pairs), 5.10% of protein deviates >2Å |
| % helix | PCA=41%, LT=41.1% |
| % sheet | PCA=21.4%, LT=21.1% |
| Residues structurally changed | 3.03% (19 residues) |
| McNemar test for helices | McNemar’s X2=0.08, df = 1, p-value =0.7 |
| McNemar test for strands | McNemar's X2=0.7, df = 1, p-value = 0.4 |
| Average helix length | PCA=4.28 Å, LT=4.3 Å |
| Paired t-test for differences in helix length | t = -0.16, df = 59, p-value =0.8 |
| Average β-strand length | PCA=1.41 Å, LT=1.39 Å |
| Paired t-test for differences in strand length | t = 0.3, df = 94, p-value = 0.7 |
| Differences in heme distances | Mean difference = 0.3 Å; t = 19.4, df = 675, p-value = 2.2x10^-16^, 95% CI: 0.27 to 0.34 |
Supplementary Figure S3. (I) Tertiary structure predictions made for OscI proteins using alphafold. The structures were visualized and heme positions were labeled in ChimeraX. Heme localizations in the individual proteins are highlighted in purple. For structural comparisons, the predicted structures were superimposed using Matchmaker and colored by the calculated per residue Cα-RMSD measurement. Residues are colored on a continuous gradient: Blue indicates the highest similarity (RMSD < 0A), white indicates intermediate difference (RMSD ~ 1 A), and red indicates the largest structural difference (RSD > 2 A). Secondary structures predicted by alphafold were also analyzed in DSSP and statistics comparing DSSP results were done with the R-stats package.

## Slide 10
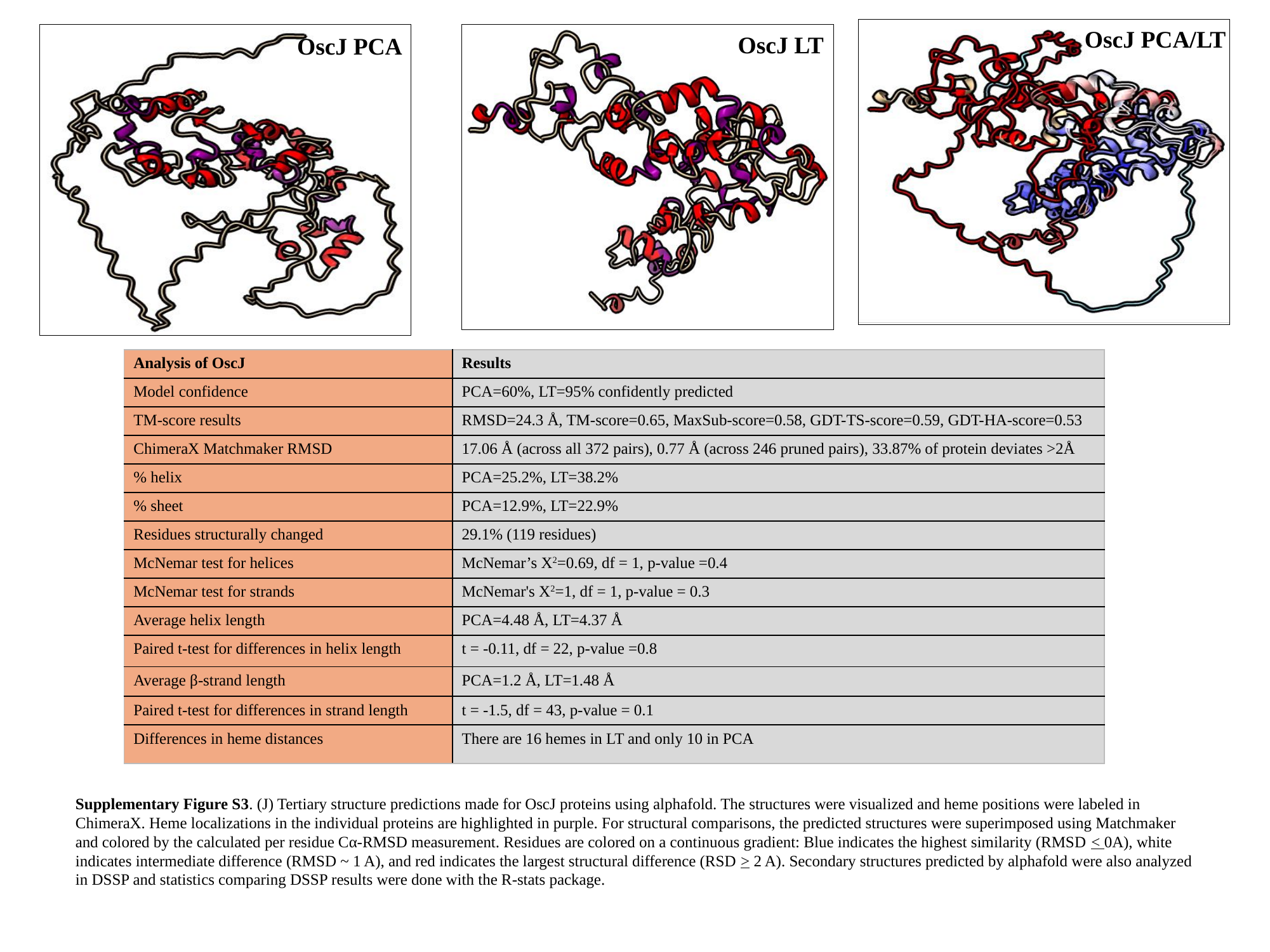

OscJ PCA/LT
OscJ LT
OscJ PCA
| Analysis of OscJ | Results |
| --- | --- |
| Model confidence | PCA=60%, LT=95% confidently predicted |
| TM-score results | RMSD=24.3 Å, TM-score=0.65, MaxSub-score=0.58, GDT-TS-score=0.59, GDT-HA-score=0.53 |
| ChimeraX Matchmaker RMSD | 17.06 Å (across all 372 pairs), 0.77 Å (across 246 pruned pairs), 33.87% of protein deviates >2Å |
| % helix | PCA=25.2%, LT=38.2% |
| % sheet | PCA=12.9%, LT=22.9% |
| Residues structurally changed | 29.1% (119 residues) |
| McNemar test for helices | McNemar’s X2=0.69, df = 1, p-value =0.4 |
| McNemar test for strands | McNemar's X2=1, df = 1, p-value = 0.3 |
| Average helix length | PCA=4.48 Å, LT=4.37 Å |
| Paired t-test for differences in helix length | t = -0.11, df = 22, p-value =0.8 |
| Average β-strand length | PCA=1.2 Å, LT=1.48 Å |
| Paired t-test for differences in strand length | t = -1.5, df = 43, p-value = 0.1 |
| Differences in heme distances | There are 16 hemes in LT and only 10 in PCA |
Supplementary Figure S3. (J) Tertiary structure predictions made for OscJ proteins using alphafold. The structures were visualized and heme positions were labeled in ChimeraX. Heme localizations in the individual proteins are highlighted in purple. For structural comparisons, the predicted structures were superimposed using Matchmaker and colored by the calculated per residue Cα-RMSD measurement. Residues are colored on a continuous gradient: Blue indicates the highest similarity (RMSD < 0A), white indicates intermediate difference (RMSD ~ 1 A), and red indicates the largest structural difference (RSD > 2 A). Secondary structures predicted by alphafold were also analyzed in DSSP and statistics comparing DSSP results were done with the R-stats package.

## Slide 11
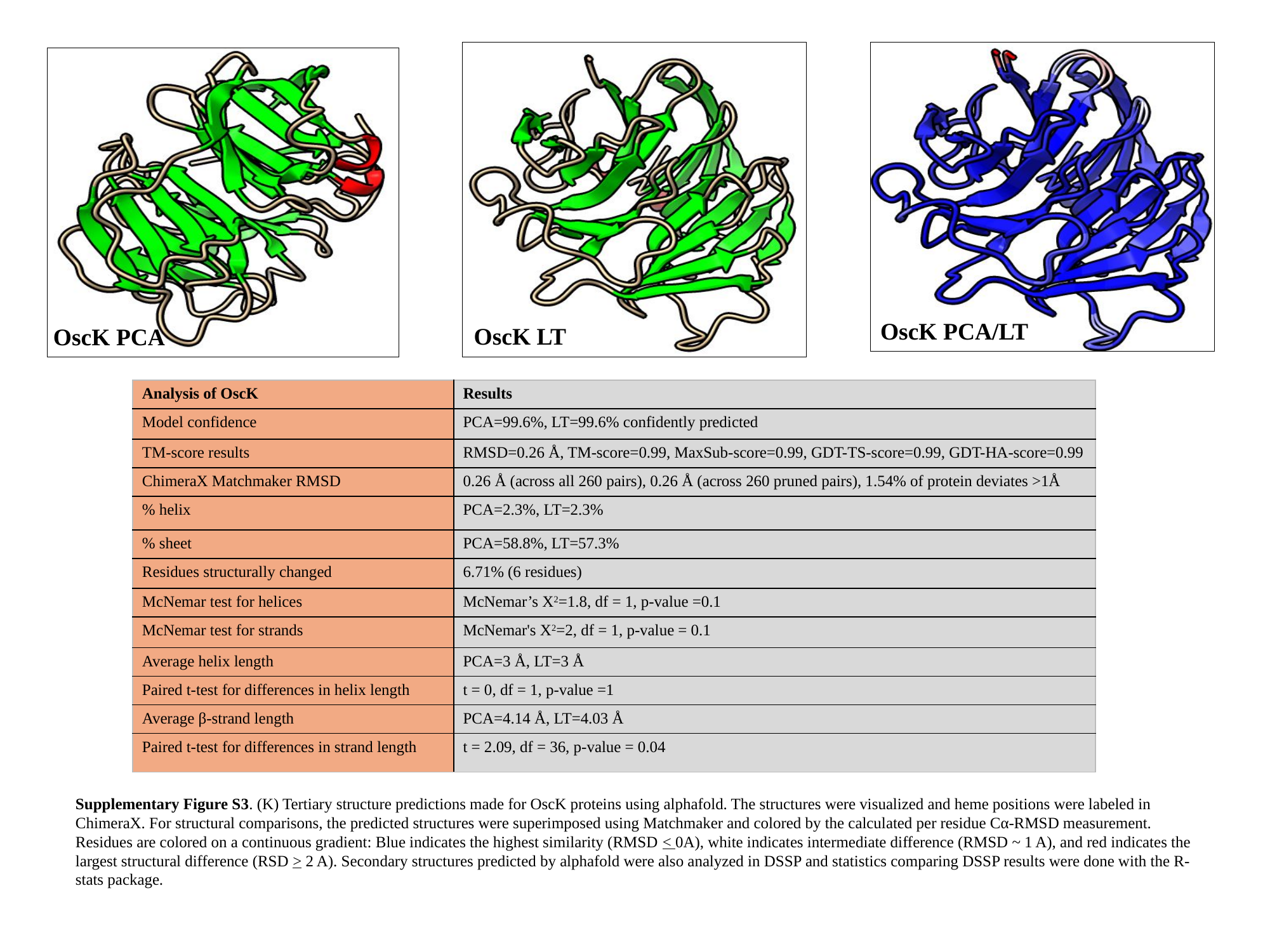

OscK PCA/LT
OscK LT
OscK PCA
| Analysis of OscK | Results |
| --- | --- |
| Model confidence | PCA=99.6%, LT=99.6% confidently predicted |
| TM-score results | RMSD=0.26 Å, TM-score=0.99, MaxSub-score=0.99, GDT-TS-score=0.99, GDT-HA-score=0.99 |
| ChimeraX Matchmaker RMSD | 0.26 Å (across all 260 pairs), 0.26 Å (across 260 pruned pairs), 1.54% of protein deviates >1Å |
| % helix | PCA=2.3%, LT=2.3% |
| % sheet | PCA=58.8%, LT=57.3% |
| Residues structurally changed | 6.71% (6 residues) |
| McNemar test for helices | McNemar’s X2=1.8, df = 1, p-value =0.1 |
| McNemar test for strands | McNemar's X2=2, df = 1, p-value = 0.1 |
| Average helix length | PCA=3 Å, LT=3 Å |
| Paired t-test for differences in helix length | t = 0, df = 1, p-value =1 |
| Average β-strand length | PCA=4.14 Å, LT=4.03 Å |
| Paired t-test for differences in strand length | t = 2.09, df = 36, p-value = 0.04 |
Supplementary Figure S3. (K) Tertiary structure predictions made for OscK proteins using alphafold. The structures were visualized and heme positions were labeled in ChimeraX. For structural comparisons, the predicted structures were superimposed using Matchmaker and colored by the calculated per residue Cα-RMSD measurement. Residues are colored on a continuous gradient: Blue indicates the highest similarity (RMSD < 0A), white indicates intermediate difference (RMSD ~ 1 A), and red indicates the largest structural difference (RSD > 2 A). Secondary structures predicted by alphafold were also analyzed in DSSP and statistics comparing DSSP results were done with the R-stats package.
