# Supplementary material for: Evolution for enhanced extracellular electron transfer in Geobacter sulfurreducens over seventeen years of continuous current generation
Source: Front Microbiol. 2026 May 8;17:1771963. doi: 10.3389/fmicb.2026.1771963 (PMC13194489; doi:10.3389/fmicb.2026.1771963)
Supplement: Supplementary file 1 [file Supplementary_file_1.zip › Supplementary Figure 4.PPTX]

## Slide 1
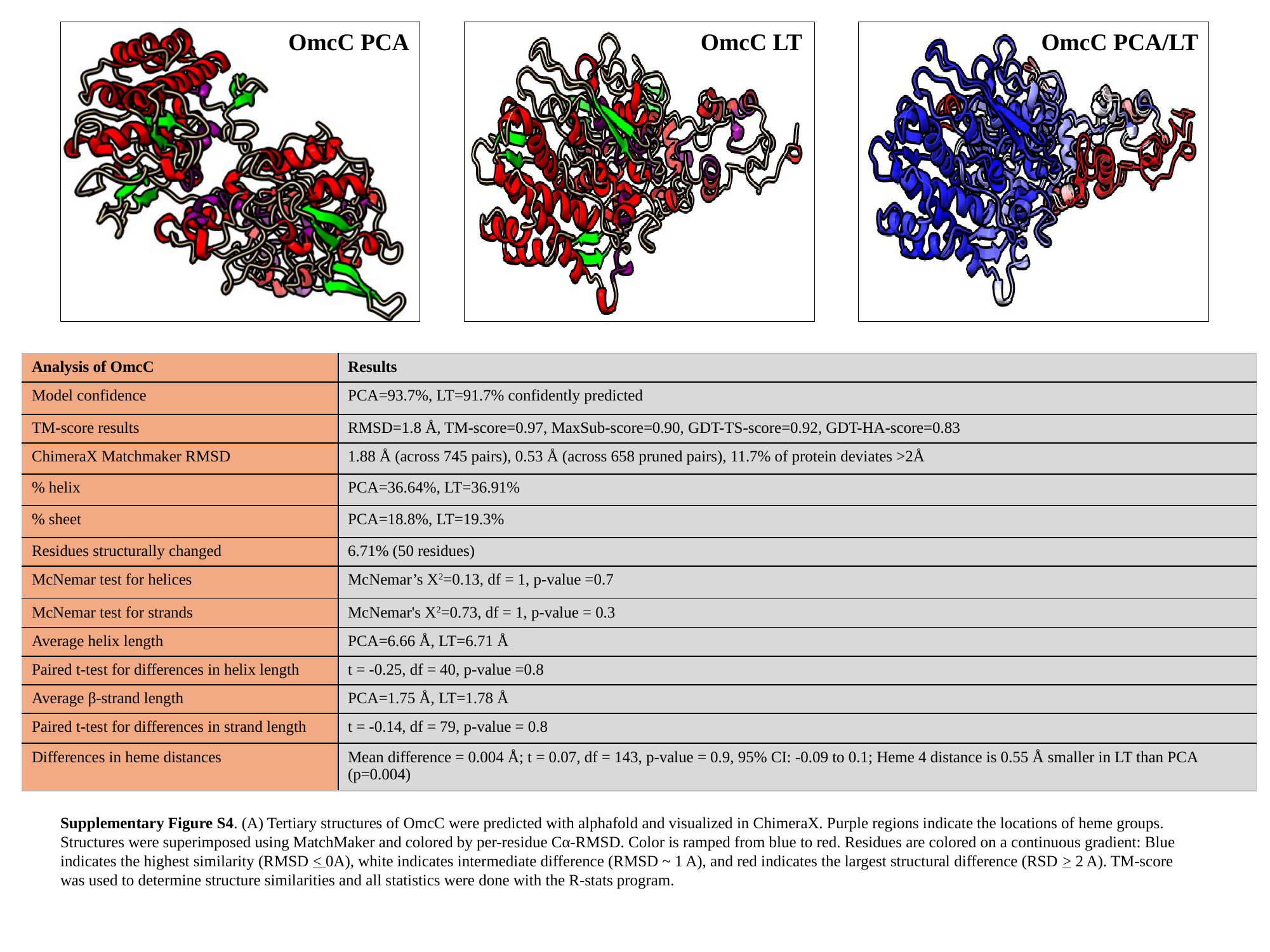

OmcC PCA
OmcC LT
OmcC PCA/LT
| Analysis of OmcC | Results |
| --- | --- |
| Model confidence | PCA=93.7%, LT=91.7% confidently predicted |
| TM-score results | RMSD=1.8 Å, TM-score=0.97, MaxSub-score=0.90, GDT-TS-score=0.92, GDT-HA-score=0.83 |
| ChimeraX Matchmaker RMSD | 1.88 Å (across 745 pairs), 0.53 Å (across 658 pruned pairs), 11.7% of protein deviates >2Å |
| % helix | PCA=36.64%, LT=36.91% |
| % sheet | PCA=18.8%, LT=19.3% |
| Residues structurally changed | 6.71% (50 residues) |
| McNemar test for helices | McNemar’s X2=0.13, df = 1, p-value =0.7 |
| McNemar test for strands | McNemar's X2=0.73, df = 1, p-value = 0.3 |
| Average helix length | PCA=6.66 Å, LT=6.71 Å |
| Paired t-test for differences in helix length | t = -0.25, df = 40, p-value =0.8 |
| Average β-strand length | PCA=1.75 Å, LT=1.78 Å |
| Paired t-test for differences in strand length | t = -0.14, df = 79, p-value = 0.8 |
| Differences in heme distances | Mean difference = 0.004 Å; t = 0.07, df = 143, p-value = 0.9, 95% CI: -0.09 to 0.1; Heme 4 distance is 0.55 Å smaller in LT than PCA (p=0.004) |
Supplementary Figure S4. (A) Tertiary structures of OmcC were predicted with alphafold and visualized in ChimeraX. Purple regions indicate the locations of heme groups. Structures were superimposed using MatchMaker and colored by per-residue Cα-RMSD. Color is ramped from blue to red. Residues are colored on a continuous gradient: Blue indicates the highest similarity (RMSD < 0A), white indicates intermediate difference (RMSD ~ 1 A), and red indicates the largest structural difference (RSD > 2 A). TM-score was used to determine structure similarities and all statistics were done with the R-stats program.

## Slide 2
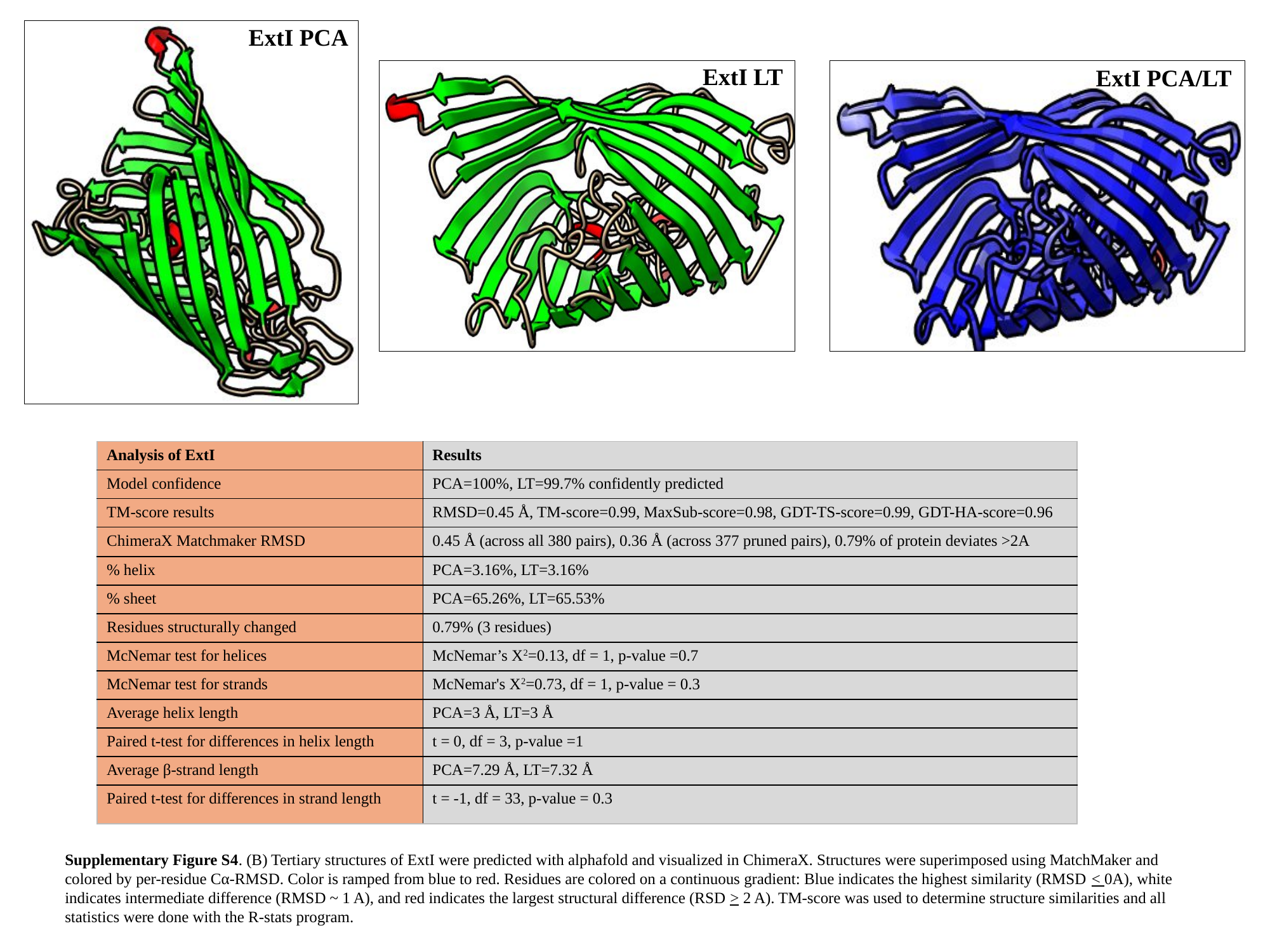

ExtI PCA
ExtI LT
ExtI PCA/LT
| Analysis of ExtI | Results |
| --- | --- |
| Model confidence | PCA=100%, LT=99.7% confidently predicted |
| TM-score results | RMSD=0.45 Å, TM-score=0.99, MaxSub-score=0.98, GDT-TS-score=0.99, GDT-HA-score=0.96 |
| ChimeraX Matchmaker RMSD | 0.45 Å (across all 380 pairs), 0.36 Å (across 377 pruned pairs), 0.79% of protein deviates >2A |
| % helix | PCA=3.16%, LT=3.16% |
| % sheet | PCA=65.26%, LT=65.53% |
| Residues structurally changed | 0.79% (3 residues) |
| McNemar test for helices | McNemar’s X2=0.13, df = 1, p-value =0.7 |
| McNemar test for strands | McNemar's X2=0.73, df = 1, p-value = 0.3 |
| Average helix length | PCA=3 Å, LT=3 Å |
| Paired t-test for differences in helix length | t = 0, df = 3, p-value =1 |
| Average β-strand length | PCA=7.29 Å, LT=7.32 Å |
| Paired t-test for differences in strand length | t = -1, df = 33, p-value = 0.3 |
Supplementary Figure S4. (B) Tertiary structures of ExtI were predicted with alphafold and visualized in ChimeraX. Structures were superimposed using MatchMaker and colored by per-residue Cα-RMSD. Color is ramped from blue to red. Residues are colored on a continuous gradient: Blue indicates the highest similarity (RMSD < 0A), white indicates intermediate difference (RMSD ~ 1 A), and red indicates the largest structural difference (RSD > 2 A). TM-score was used to determine structure similarities and all statistics were done with the R-stats program.

## Slide 3
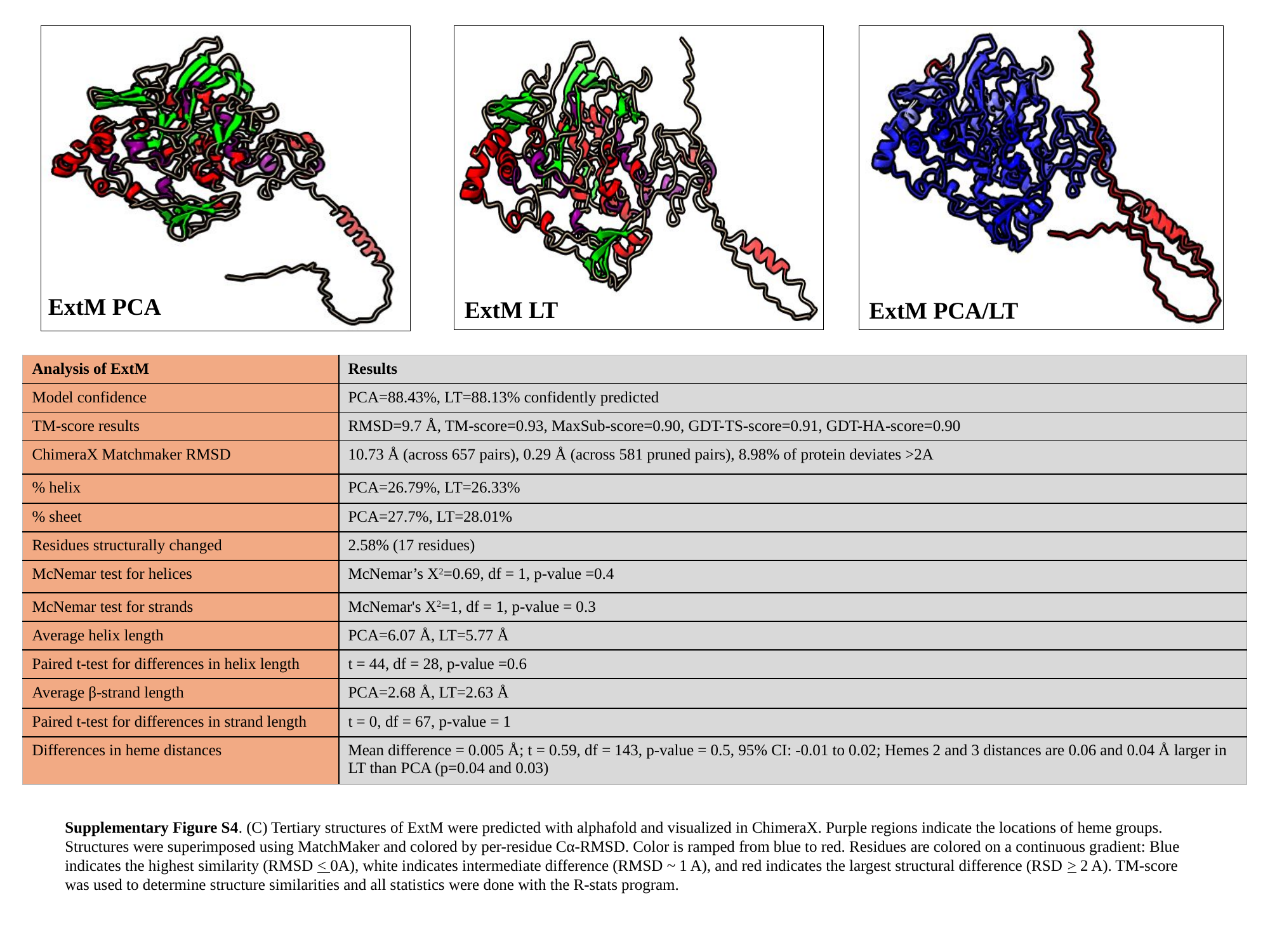

ExtM PCA
ExtM LT
ExtM PCA/LT
| Analysis of ExtM | Results |
| --- | --- |
| Model confidence | PCA=88.43%, LT=88.13% confidently predicted |
| TM-score results | RMSD=9.7 Å, TM-score=0.93, MaxSub-score=0.90, GDT-TS-score=0.91, GDT-HA-score=0.90 |
| ChimeraX Matchmaker RMSD | 10.73 Å (across 657 pairs), 0.29 Å (across 581 pruned pairs), 8.98% of protein deviates >2A |
| % helix | PCA=26.79%, LT=26.33% |
| % sheet | PCA=27.7%, LT=28.01% |
| Residues structurally changed | 2.58% (17 residues) |
| McNemar test for helices | McNemar’s X2=0.69, df = 1, p-value =0.4 |
| McNemar test for strands | McNemar's X2=1, df = 1, p-value = 0.3 |
| Average helix length | PCA=6.07 Å, LT=5.77 Å |
| Paired t-test for differences in helix length | t = 44, df = 28, p-value =0.6 |
| Average β-strand length | PCA=2.68 Å, LT=2.63 Å |
| Paired t-test for differences in strand length | t = 0, df = 67, p-value = 1 |
| Differences in heme distances | Mean difference = 0.005 Å; t = 0.59, df = 143, p-value = 0.5, 95% CI: -0.01 to 0.02; Hemes 2 and 3 distances are 0.06 and 0.04 Å larger in LT than PCA (p=0.04 and 0.03) |
Supplementary Figure S4. (C) Tertiary structures of ExtM were predicted with alphafold and visualized in ChimeraX. Purple regions indicate the locations of heme groups. Structures were superimposed using MatchMaker and colored by per-residue Cα-RMSD. Color is ramped from blue to red. Residues are colored on a continuous gradient: Blue indicates the highest similarity (RMSD < 0A), white indicates intermediate difference (RMSD ~ 1 A), and red indicates the largest structural difference (RSD > 2 A). TM-score was used to determine structure similarities and all statistics were done with the R-stats program.

## Slide 4
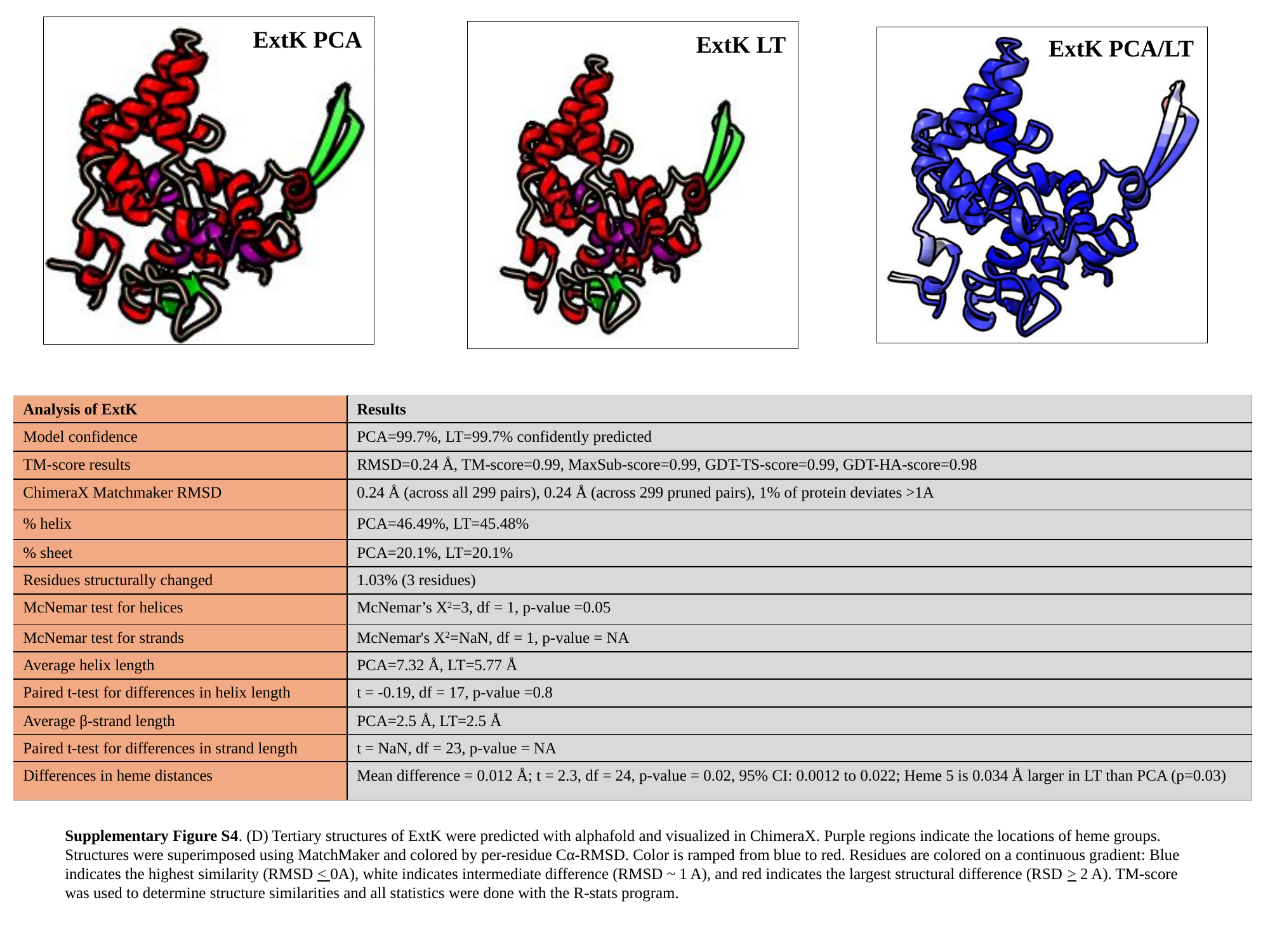

ExtK PCA
ExtK LT
ExtK PCA/LT
| Analysis of ExtK | Results |
| --- | --- |
| Model confidence | PCA=99.7%, LT=99.7% confidently predicted |
| TM-score results | RMSD=0.24 Å, TM-score=0.99, MaxSub-score=0.99, GDT-TS-score=0.99, GDT-HA-score=0.98 |
| ChimeraX Matchmaker RMSD | 0.24 Å (across all 299 pairs), 0.24 Å (across 299 pruned pairs), 1% of protein deviates >1A |
| % helix | PCA=46.49%, LT=45.48% |
| % sheet | PCA=20.1%, LT=20.1% |
| Residues structurally changed | 1.03% (3 residues) |
| McNemar test for helices | McNemar’s X2=3, df = 1, p-value =0.05 |
| McNemar test for strands | McNemar's X2=NaN, df = 1, p-value = NA |
| Average helix length | PCA=7.32 Å, LT=5.77 Å |
| Paired t-test for differences in helix length | t = -0.19, df = 17, p-value =0.8 |
| Average β-strand length | PCA=2.5 Å, LT=2.5 Å |
| Paired t-test for differences in strand length | t = NaN, df = 23, p-value = NA |
| Differences in heme distances | Mean difference = 0.012 Å; t = 2.3, df = 24, p-value = 0.02, 95% CI: 0.0012 to 0.022; Heme 5 is 0.034 Å larger in LT than PCA (p=0.03) |
Supplementary Figure S4. (D) Tertiary structures of ExtK were predicted with alphafold and visualized in ChimeraX. Purple regions indicate the locations of heme groups. Structures were superimposed using MatchMaker and colored by per-residue Cα-RMSD. Color is ramped from blue to red. Residues are colored on a continuous gradient: Blue indicates the highest similarity (RMSD < 0A), white indicates intermediate difference (RMSD ~ 1 A), and red indicates the largest structural difference (RSD > 2 A). TM-score was used to determine structure similarities and all statistics were done with the R-stats program.

## Slide 5
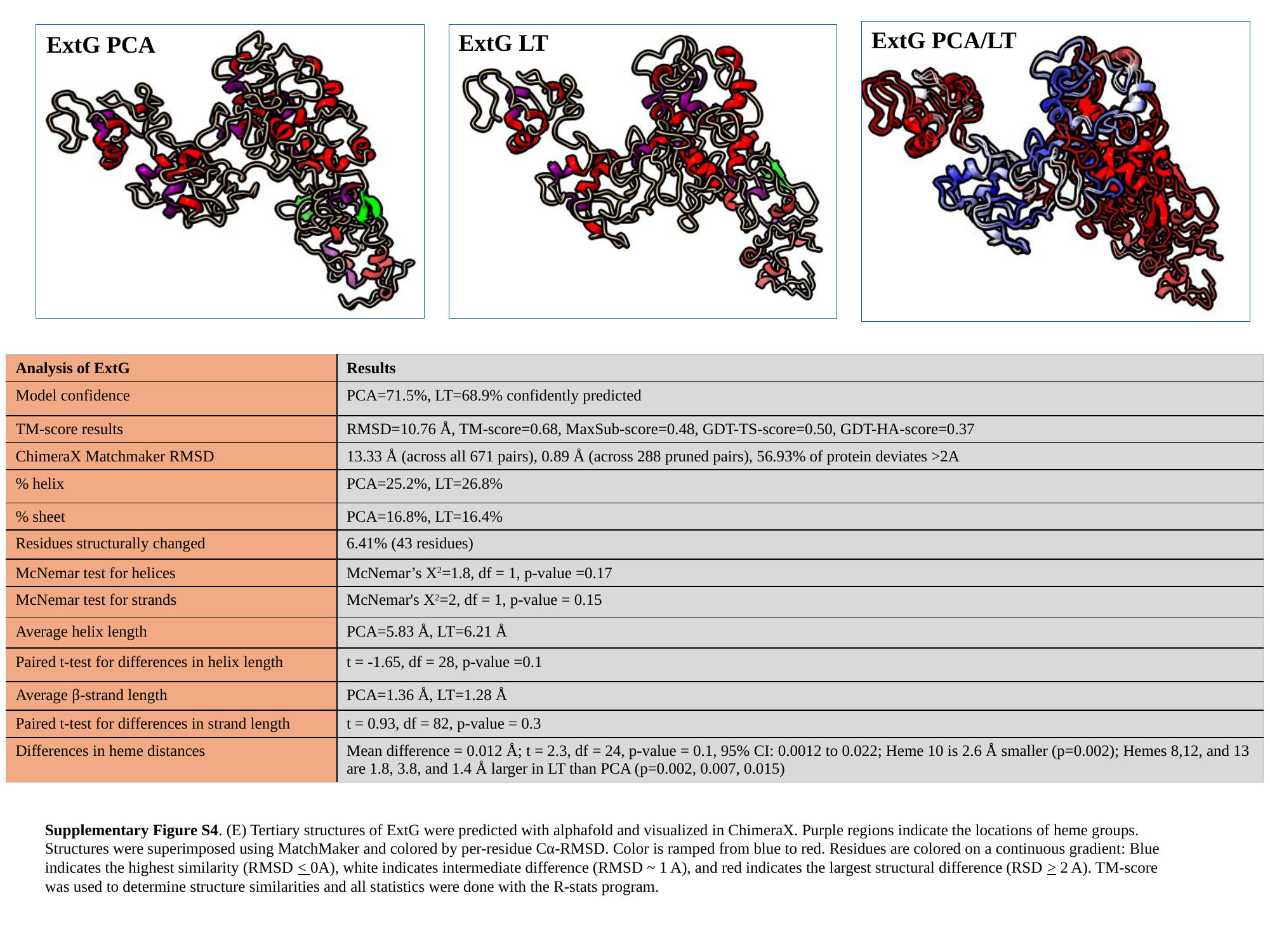

ExtG PCA/LT
ExtG LT
ExtG PCA
| Analysis of ExtG | Results |
| --- | --- |
| Model confidence | PCA=71.5%, LT=68.9% confidently predicted |
| TM-score results | RMSD=10.76 Å, TM-score=0.68, MaxSub-score=0.48, GDT-TS-score=0.50, GDT-HA-score=0.37 |
| ChimeraX Matchmaker RMSD | 13.33 Å (across all 671 pairs), 0.89 Å (across 288 pruned pairs), 56.93% of protein deviates >2A |
| % helix | PCA=25.2%, LT=26.8% |
| % sheet | PCA=16.8%, LT=16.4% |
| Residues structurally changed | 6.41% (43 residues) |
| McNemar test for helices | McNemar’s X2=1.8, df = 1, p-value =0.17 |
| McNemar test for strands | McNemar's X2=2, df = 1, p-value = 0.15 |
| Average helix length | PCA=5.83 Å, LT=6.21 Å |
| Paired t-test for differences in helix length | t = -1.65, df = 28, p-value =0.1 |
| Average β-strand length | PCA=1.36 Å, LT=1.28 Å |
| Paired t-test for differences in strand length | t = 0.93, df = 82, p-value = 0.3 |
| Differences in heme distances | Mean difference = 0.012 Å; t = 2.3, df = 24, p-value = 0.1, 95% CI: 0.0012 to 0.022; Heme 10 is 2.6 Å smaller (p=0.002); Hemes 8,12, and 13 are 1.8, 3.8, and 1.4 Å larger in LT than PCA (p=0.002, 0.007, 0.015) |
Supplementary Figure S4. (E) Tertiary structures of ExtG were predicted with alphafold and visualized in ChimeraX. Purple regions indicate the locations of heme groups. Structures were superimposed using MatchMaker and colored by per-residue Cα-RMSD. Color is ramped from blue to red. Residues are colored on a continuous gradient: Blue indicates the highest similarity (RMSD < 0A), white indicates intermediate difference (RMSD ~ 1 A), and red indicates the largest structural difference (RSD > 2 A). TM-score was used to determine structure similarities and all statistics were done with the R-stats program.

## Slide 6
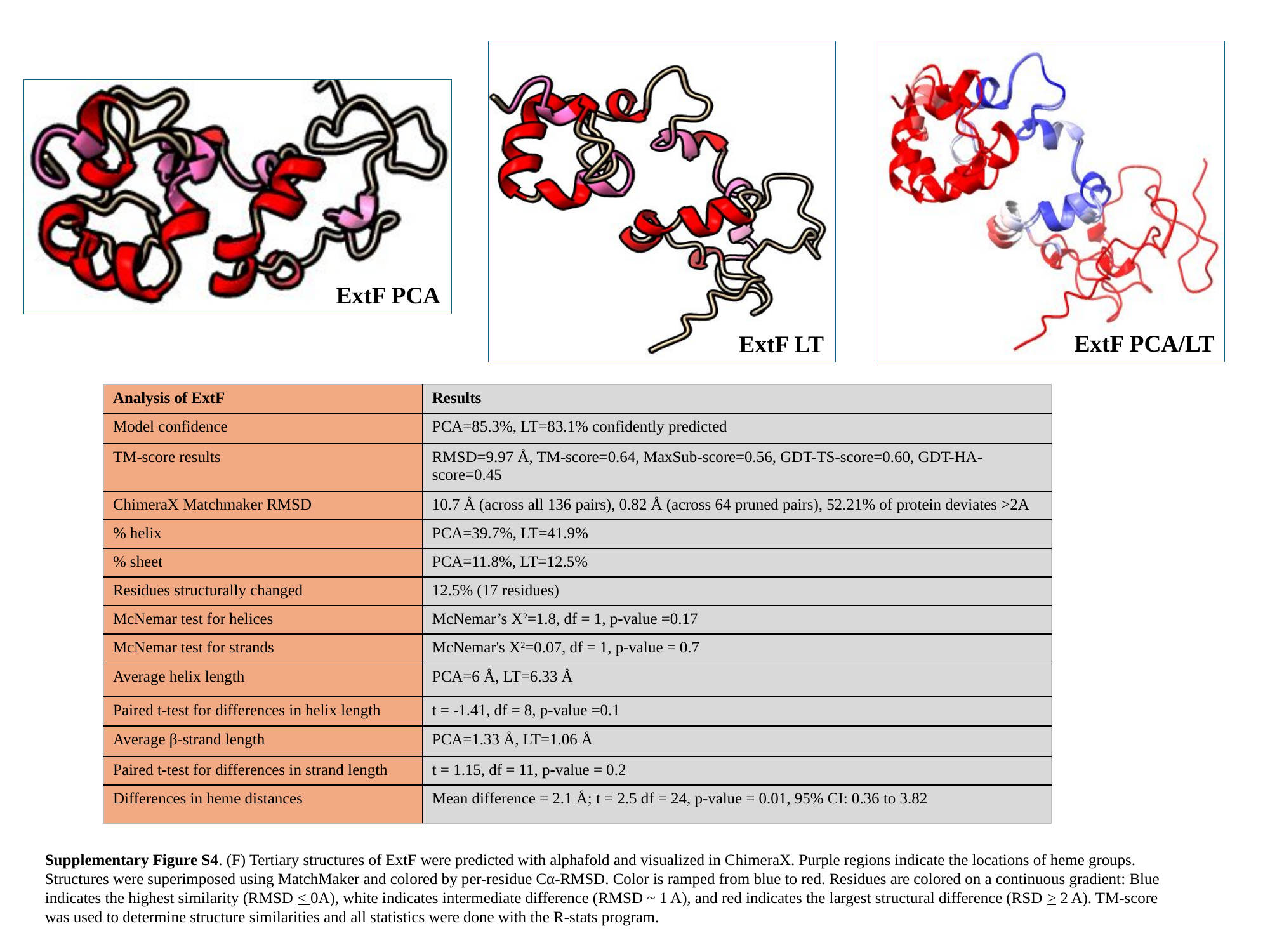

ExtF PCA
ExtF PCA/LT
ExtF LT
| Analysis of ExtF | Results |
| --- | --- |
| Model confidence | PCA=85.3%, LT=83.1% confidently predicted |
| TM-score results | RMSD=9.97 Å, TM-score=0.64, MaxSub-score=0.56, GDT-TS-score=0.60, GDT-HA-score=0.45 |
| ChimeraX Matchmaker RMSD | 10.7 Å (across all 136 pairs), 0.82 Å (across 64 pruned pairs), 52.21% of protein deviates >2A |
| % helix | PCA=39.7%, LT=41.9% |
| % sheet | PCA=11.8%, LT=12.5% |
| Residues structurally changed | 12.5% (17 residues) |
| McNemar test for helices | McNemar’s X2=1.8, df = 1, p-value =0.17 |
| McNemar test for strands | McNemar's X2=0.07, df = 1, p-value = 0.7 |
| Average helix length | PCA=6 Å, LT=6.33 Å |
| Paired t-test for differences in helix length | t = -1.41, df = 8, p-value =0.1 |
| Average β-strand length | PCA=1.33 Å, LT=1.06 Å |
| Paired t-test for differences in strand length | t = 1.15, df = 11, p-value = 0.2 |
| Differences in heme distances | Mean difference = 2.1 Å; t = 2.5 df = 24, p-value = 0.01, 95% CI: 0.36 to 3.82 |
Supplementary Figure S4. (F) Tertiary structures of ExtF were predicted with alphafold and visualized in ChimeraX. Purple regions indicate the locations of heme groups. Structures were superimposed using MatchMaker and colored by per-residue Cα-RMSD. Color is ramped from blue to red. Residues are colored on a continuous gradient: Blue indicates the highest similarity (RMSD < 0A), white indicates intermediate difference (RMSD ~ 1 A), and red indicates the largest structural difference (RSD > 2 A). TM-score was used to determine structure similarities and all statistics were done with the R-stats program.

## Slide 7
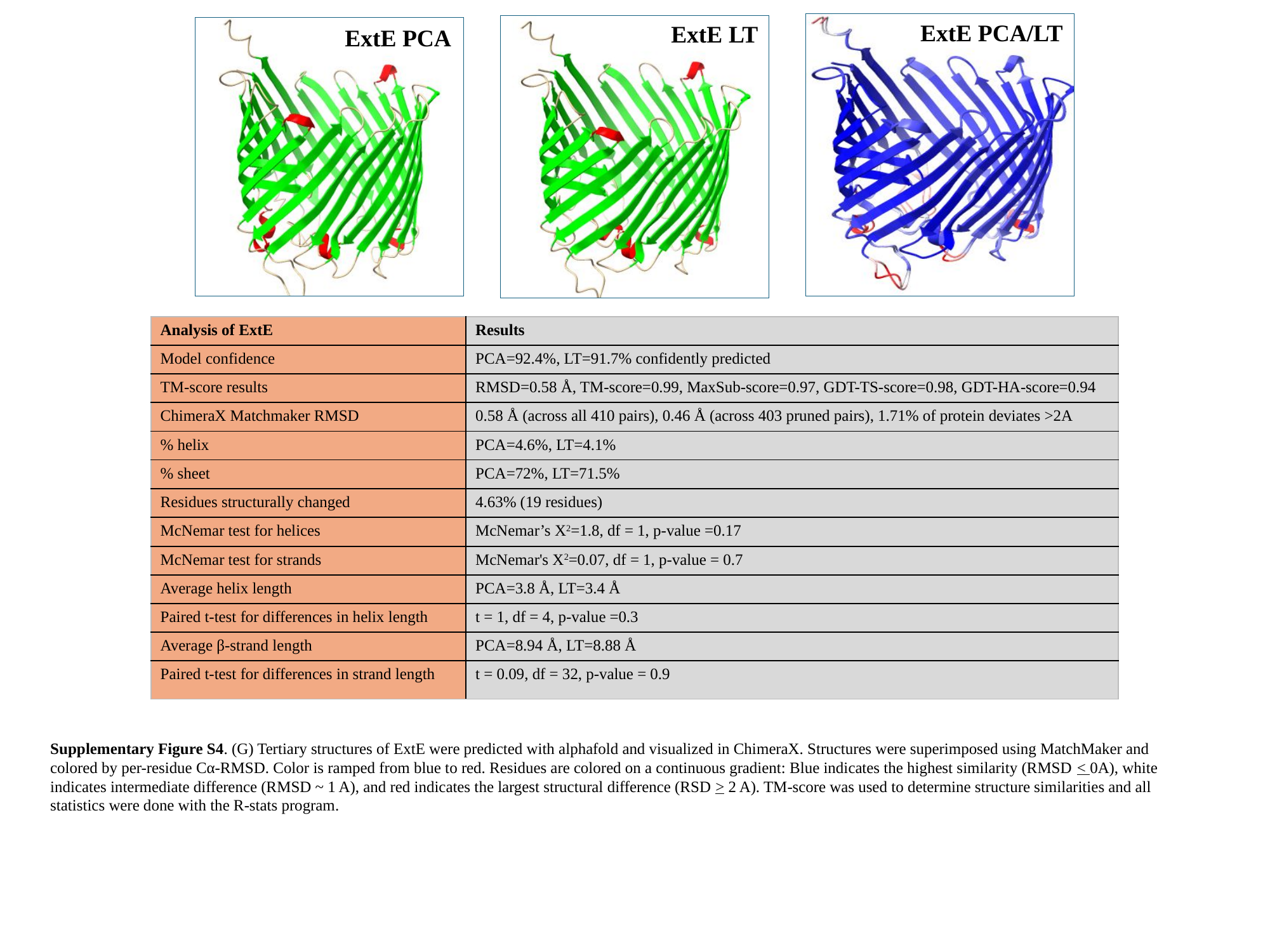

ExtE PCA/LT
ExtE LT
ExtE PCA
| Analysis of ExtE | Results |
| --- | --- |
| Model confidence | PCA=92.4%, LT=91.7% confidently predicted |
| TM-score results | RMSD=0.58 Å, TM-score=0.99, MaxSub-score=0.97, GDT-TS-score=0.98, GDT-HA-score=0.94 |
| ChimeraX Matchmaker RMSD | 0.58 Å (across all 410 pairs), 0.46 Å (across 403 pruned pairs), 1.71% of protein deviates >2A |
| % helix | PCA=4.6%, LT=4.1% |
| % sheet | PCA=72%, LT=71.5% |
| Residues structurally changed | 4.63% (19 residues) |
| McNemar test for helices | McNemar’s X2=1.8, df = 1, p-value =0.17 |
| McNemar test for strands | McNemar's X2=0.07, df = 1, p-value = 0.7 |
| Average helix length | PCA=3.8 Å, LT=3.4 Å |
| Paired t-test for differences in helix length | t = 1, df = 4, p-value =0.3 |
| Average β-strand length | PCA=8.94 Å, LT=8.88 Å |
| Paired t-test for differences in strand length | t = 0.09, df = 32, p-value = 0.9 |
Supplementary Figure S4. (G) Tertiary structures of ExtE were predicted with alphafold and visualized in ChimeraX. Structures were superimposed using MatchMaker and colored by per-residue Cα-RMSD. Color is ramped from blue to red. Residues are colored on a continuous gradient: Blue indicates the highest similarity (RMSD < 0A), white indicates intermediate difference (RMSD ~ 1 A), and red indicates the largest structural difference (RSD > 2 A). TM-score was used to determine structure similarities and all statistics were done with the R-stats program.
