# Supplementary material for: Evolution for enhanced extracellular electron transfer in Geobacter sulfurreducens over seventeen years of continuous current generation
Source: Front Microbiol. 2026 May 8;17:1771963. doi: 10.3389/fmicb.2026.1771963 (PMC13194489; doi:10.3389/fmicb.2026.1771963)
Supplement: Supplementary file 1 [file Supplementary_file_1.zip › Supplementary Figure 5.PPTX]

## Slide 1
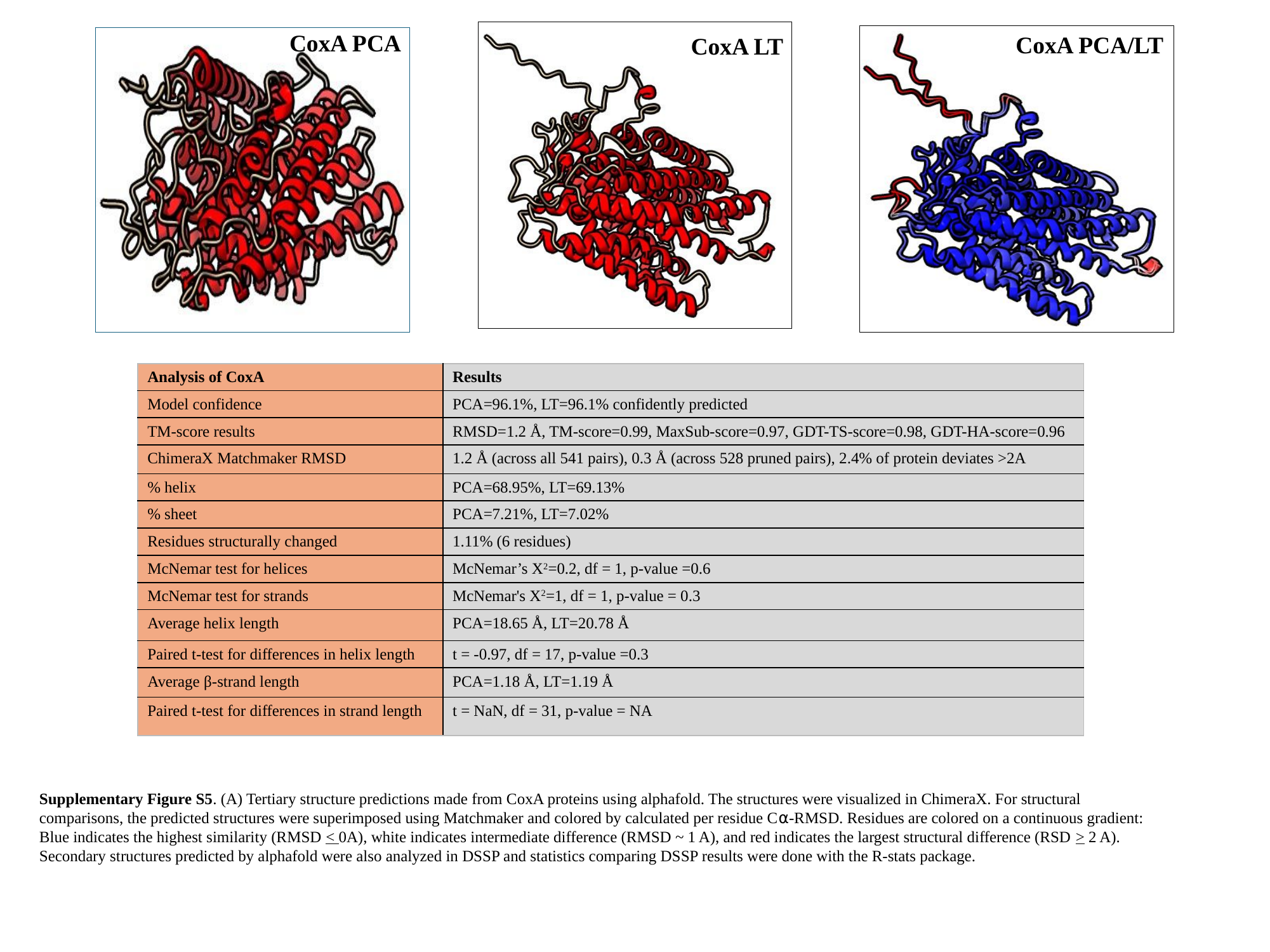

CoxA PCA
CoxA PCA/LT
CoxA LT
| Analysis of CoxA | Results |
| --- | --- |
| Model confidence | PCA=96.1%, LT=96.1% confidently predicted |
| TM-score results | RMSD=1.2 Å, TM-score=0.99, MaxSub-score=0.97, GDT-TS-score=0.98, GDT-HA-score=0.96 |
| ChimeraX Matchmaker RMSD | 1.2 Å (across all 541 pairs), 0.3 Å (across 528 pruned pairs), 2.4% of protein deviates >2A |
| % helix | PCA=68.95%, LT=69.13% |
| % sheet | PCA=7.21%, LT=7.02% |
| Residues structurally changed | 1.11% (6 residues) |
| McNemar test for helices | McNemar’s X2=0.2, df = 1, p-value =0.6 |
| McNemar test for strands | McNemar's X2=1, df = 1, p-value = 0.3 |
| Average helix length | PCA=18.65 Å, LT=20.78 Å |
| Paired t-test for differences in helix length | t = -0.97, df = 17, p-value =0.3 |
| Average β-strand length | PCA=1.18 Å, LT=1.19 Å |
| Paired t-test for differences in strand length | t = NaN, df = 31, p-value = NA |
Supplementary Figure S5. (A) Tertiary structure predictions made from CoxA proteins using alphafold. The structures were visualized in ChimeraX. For structural comparisons, the predicted structures were superimposed using Matchmaker and colored by calculated per residue Cα-RMSD. Residues are colored on a continuous gradient: Blue indicates the highest similarity (RMSD < 0A), white indicates intermediate difference (RMSD ~ 1 A), and red indicates the largest structural difference (RSD > 2 A). Secondary structures predicted by alphafold were also analyzed in DSSP and statistics comparing DSSP results were done with the R-stats package.

## Slide 2
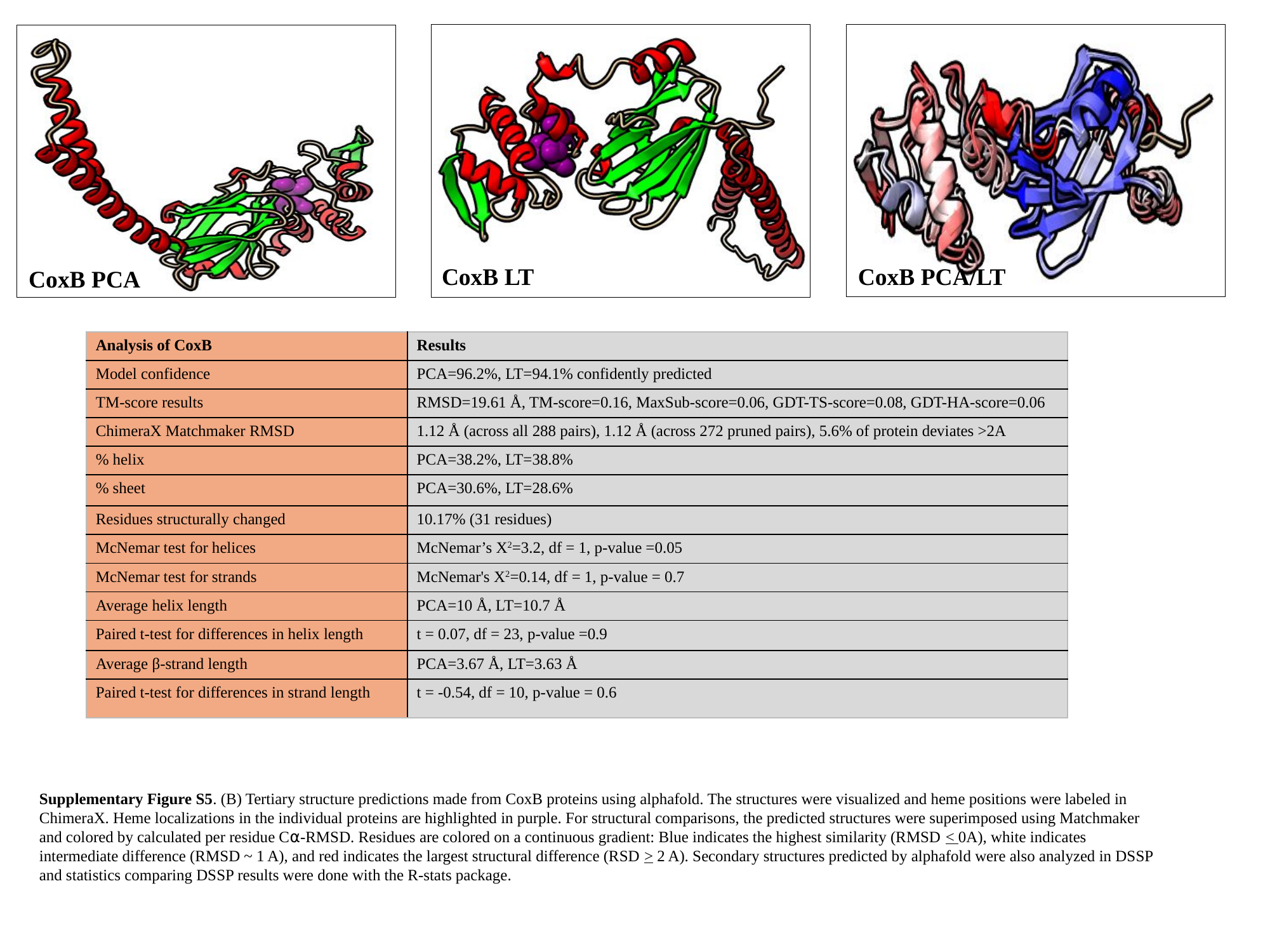

CoxB LT
CoxB PCA/LT
CoxB PCA
| Analysis of CoxB | Results |
| --- | --- |
| Model confidence | PCA=96.2%, LT=94.1% confidently predicted |
| TM-score results | RMSD=19.61 Å, TM-score=0.16, MaxSub-score=0.06, GDT-TS-score=0.08, GDT-HA-score=0.06 |
| ChimeraX Matchmaker RMSD | 1.12 Å (across all 288 pairs), 1.12 Å (across 272 pruned pairs), 5.6% of protein deviates >2A |
| % helix | PCA=38.2%, LT=38.8% |
| % sheet | PCA=30.6%, LT=28.6% |
| Residues structurally changed | 10.17% (31 residues) |
| McNemar test for helices | McNemar’s X2=3.2, df = 1, p-value =0.05 |
| McNemar test for strands | McNemar's X2=0.14, df = 1, p-value = 0.7 |
| Average helix length | PCA=10 Å, LT=10.7 Å |
| Paired t-test for differences in helix length | t = 0.07, df = 23, p-value =0.9 |
| Average β-strand length | PCA=3.67 Å, LT=3.63 Å |
| Paired t-test for differences in strand length | t = -0.54, df = 10, p-value = 0.6 |
Supplementary Figure S5. (B) Tertiary structure predictions made from CoxB proteins using alphafold. The structures were visualized and heme positions were labeled in ChimeraX. Heme localizations in the individual proteins are highlighted in purple. For structural comparisons, the predicted structures were superimposed using Matchmaker and colored by calculated per residue Cα-RMSD. Residues are colored on a continuous gradient: Blue indicates the highest similarity (RMSD < 0A), white indicates intermediate difference (RMSD ~ 1 A), and red indicates the largest structural difference (RSD > 2 A). Secondary structures predicted by alphafold were also analyzed in DSSP and statistics comparing DSSP results were done with the R-stats package.

## Slide 3
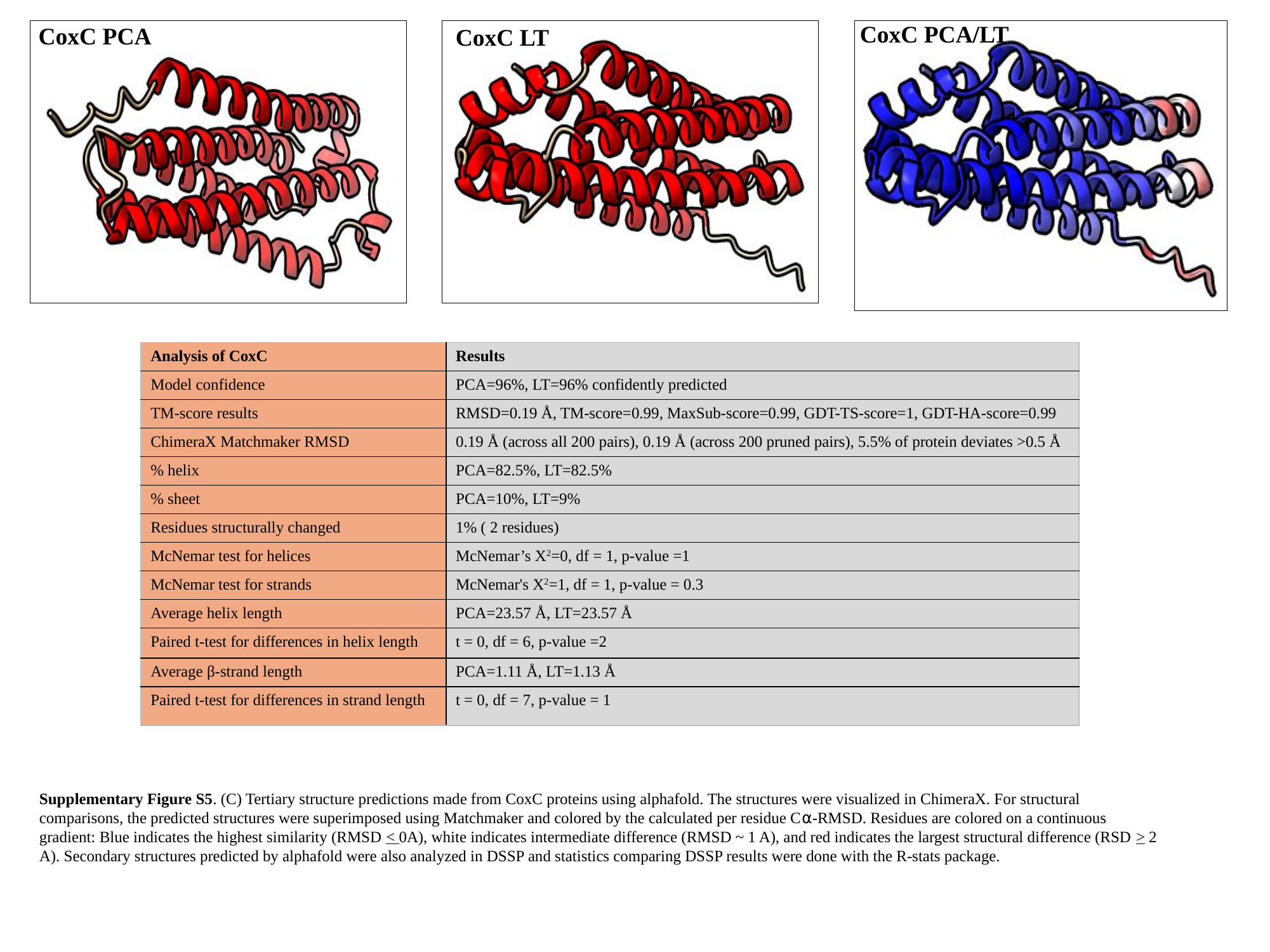

CoxC PCA/LT
CoxC PCA
CoxC LT
| Analysis of CoxC | Results |
| --- | --- |
| Model confidence | PCA=96%, LT=96% confidently predicted |
| TM-score results | RMSD=0.19 Å, TM-score=0.99, MaxSub-score=0.99, GDT-TS-score=1, GDT-HA-score=0.99 |
| ChimeraX Matchmaker RMSD | 0.19 Å (across all 200 pairs), 0.19 Å (across 200 pruned pairs), 5.5% of protein deviates >0.5 Å |
| % helix | PCA=82.5%, LT=82.5% |
| % sheet | PCA=10%, LT=9% |
| Residues structurally changed | 1% ( 2 residues) |
| McNemar test for helices | McNemar’s X2=0, df = 1, p-value =1 |
| McNemar test for strands | McNemar's X2=1, df = 1, p-value = 0.3 |
| Average helix length | PCA=23.57 Å, LT=23.57 Å |
| Paired t-test for differences in helix length | t = 0, df = 6, p-value =2 |
| Average β-strand length | PCA=1.11 Å, LT=1.13 Å |
| Paired t-test for differences in strand length | t = 0, df = 7, p-value = 1 |
Supplementary Figure S5. (C) Tertiary structure predictions made from CoxC proteins using alphafold. The structures were visualized in ChimeraX. For structural comparisons, the predicted structures were superimposed using Matchmaker and colored by the calculated per residue Cα-RMSD. Residues are colored on a continuous gradient: Blue indicates the highest similarity (RMSD < 0A), white indicates intermediate difference (RMSD ~ 1 A), and red indicates the largest structural difference (RSD > 2 A). Secondary structures predicted by alphafold were also analyzed in DSSP and statistics comparing DSSP results were done with the R-stats package.

## Slide 4
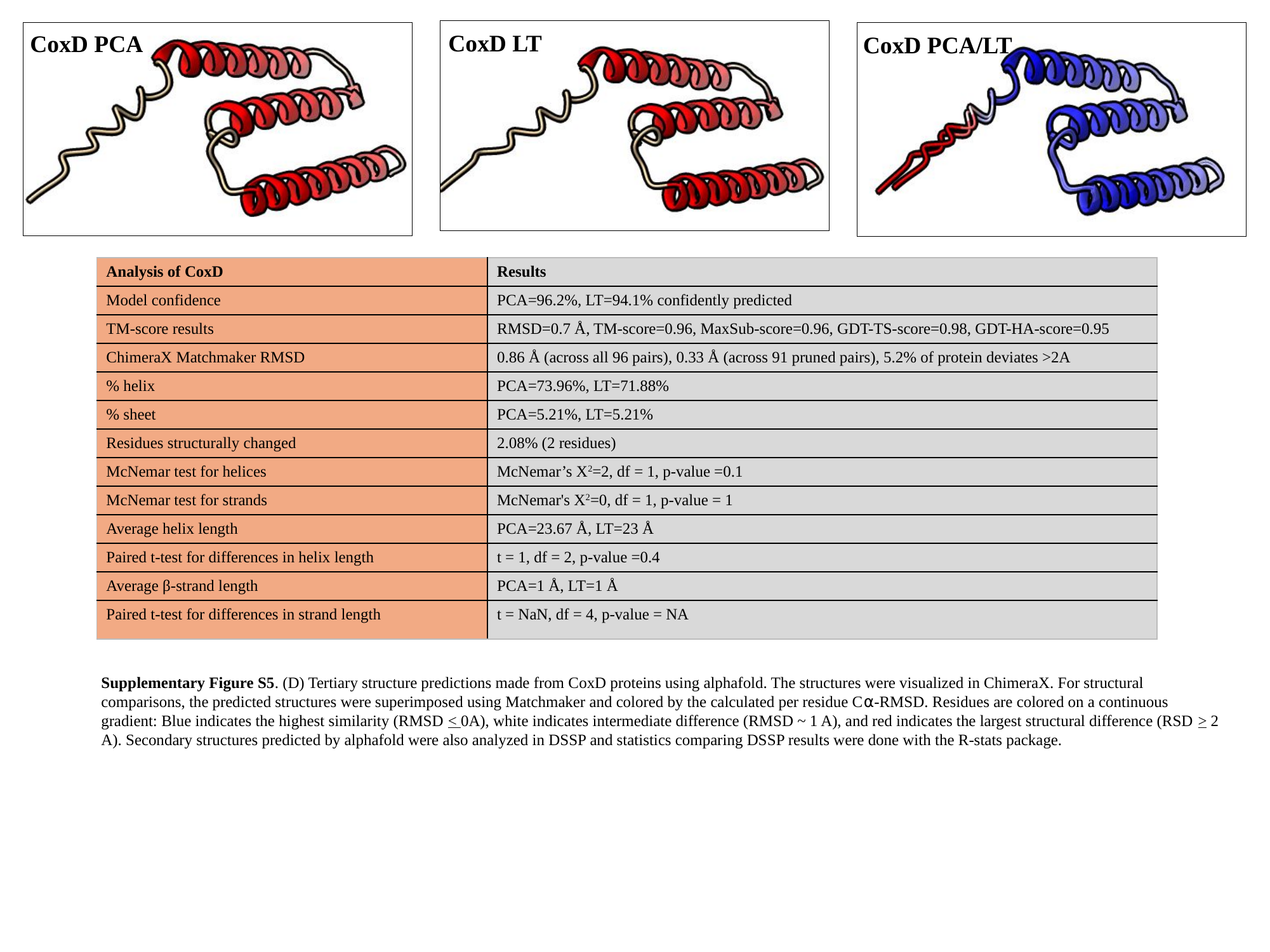

CoxD LT
CoxD PCA
CoxD PCA/LT
| Analysis of CoxD | Results |
| --- | --- |
| Model confidence | PCA=96.2%, LT=94.1% confidently predicted |
| TM-score results | RMSD=0.7 Å, TM-score=0.96, MaxSub-score=0.96, GDT-TS-score=0.98, GDT-HA-score=0.95 |
| ChimeraX Matchmaker RMSD | 0.86 Å (across all 96 pairs), 0.33 Å (across 91 pruned pairs), 5.2% of protein deviates >2A |
| % helix | PCA=73.96%, LT=71.88% |
| % sheet | PCA=5.21%, LT=5.21% |
| Residues structurally changed | 2.08% (2 residues) |
| McNemar test for helices | McNemar’s X2=2, df = 1, p-value =0.1 |
| McNemar test for strands | McNemar's X2=0, df = 1, p-value = 1 |
| Average helix length | PCA=23.67 Å, LT=23 Å |
| Paired t-test for differences in helix length | t = 1, df = 2, p-value =0.4 |
| Average β-strand length | PCA=1 Å, LT=1 Å |
| Paired t-test for differences in strand length | t = NaN, df = 4, p-value = NA |
Supplementary Figure S5. (D) Tertiary structure predictions made from CoxD proteins using alphafold. The structures were visualized in ChimeraX. For structural comparisons, the predicted structures were superimposed using Matchmaker and colored by the calculated per residue Cα-RMSD. Residues are colored on a continuous gradient: Blue indicates the highest similarity (RMSD < 0A), white indicates intermediate difference (RMSD ~ 1 A), and red indicates the largest structural difference (RSD > 2 A). Secondary structures predicted by alphafold were also analyzed in DSSP and statistics comparing DSSP results were done with the R-stats package.
