# Supplementary material for: Evolution for enhanced extracellular electron transfer in Geobacter sulfurreducens over seventeen years of continuous current generation
Source: Front Microbiol. 2026 May 8;17:1771963. doi: 10.3389/fmicb.2026.1771963 (PMC13194489; doi:10.3389/fmicb.2026.1771963)
Supplement: Supplementary file 1 [file Supplementary_file_1.zip › Supplementary Figure 6.PPTX]

## Slide 1
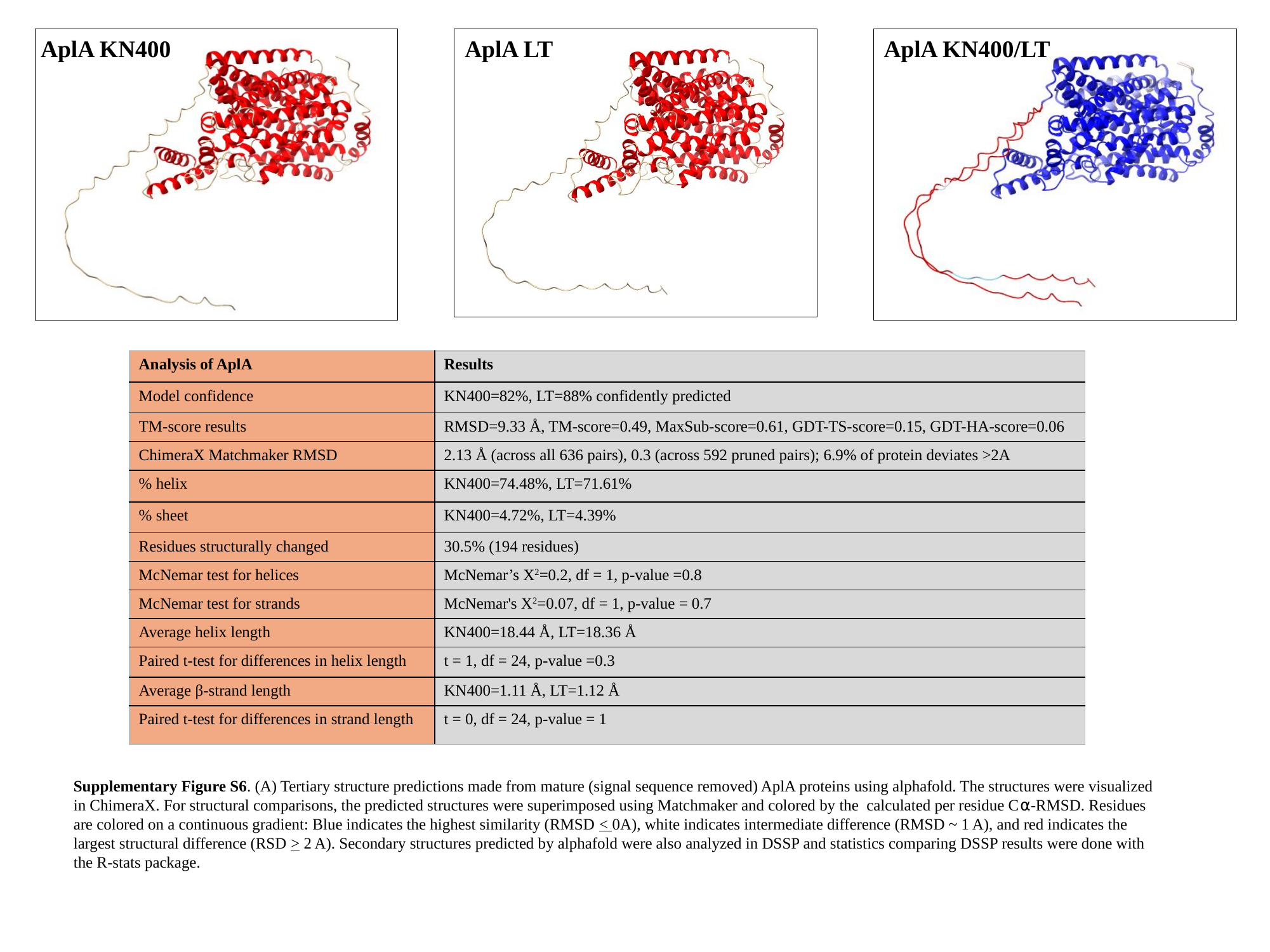

AplA KN400
AplA LT
AplA KN400/LT
| Analysis of AplA | Results |
| --- | --- |
| Model confidence | KN400=82%, LT=88% confidently predicted |
| TM-score results | RMSD=9.33 Å, TM-score=0.49, MaxSub-score=0.61, GDT-TS-score=0.15, GDT-HA-score=0.06 |
| ChimeraX Matchmaker RMSD | 2.13 Å (across all 636 pairs), 0.3 (across 592 pruned pairs); 6.9% of protein deviates >2A |
| % helix | KN400=74.48%, LT=71.61% |
| % sheet | KN400=4.72%, LT=4.39% |
| Residues structurally changed | 30.5% (194 residues) |
| McNemar test for helices | McNemar’s X2=0.2, df = 1, p-value =0.8 |
| McNemar test for strands | McNemar's X2=0.07, df = 1, p-value = 0.7 |
| Average helix length | KN400=18.44 Å, LT=18.36 Å |
| Paired t-test for differences in helix length | t = 1, df = 24, p-value =0.3 |
| Average β-strand length | KN400=1.11 Å, LT=1.12 Å |
| Paired t-test for differences in strand length | t = 0, df = 24, p-value = 1 |
Supplementary Figure S6. (A) Tertiary structure predictions made from mature (signal sequence removed) AplA proteins using alphafold. The structures were visualized in ChimeraX. For structural comparisons, the predicted structures were superimposed using Matchmaker and colored by the calculated per residue Cα-RMSD. Residues are colored on a continuous gradient: Blue indicates the highest similarity (RMSD < 0A), white indicates intermediate difference (RMSD ~ 1 A), and red indicates the largest structural difference (RSD > 2 A). Secondary structures predicted by alphafold were also analyzed in DSSP and statistics comparing DSSP results were done with the R-stats package.

## Slide 2
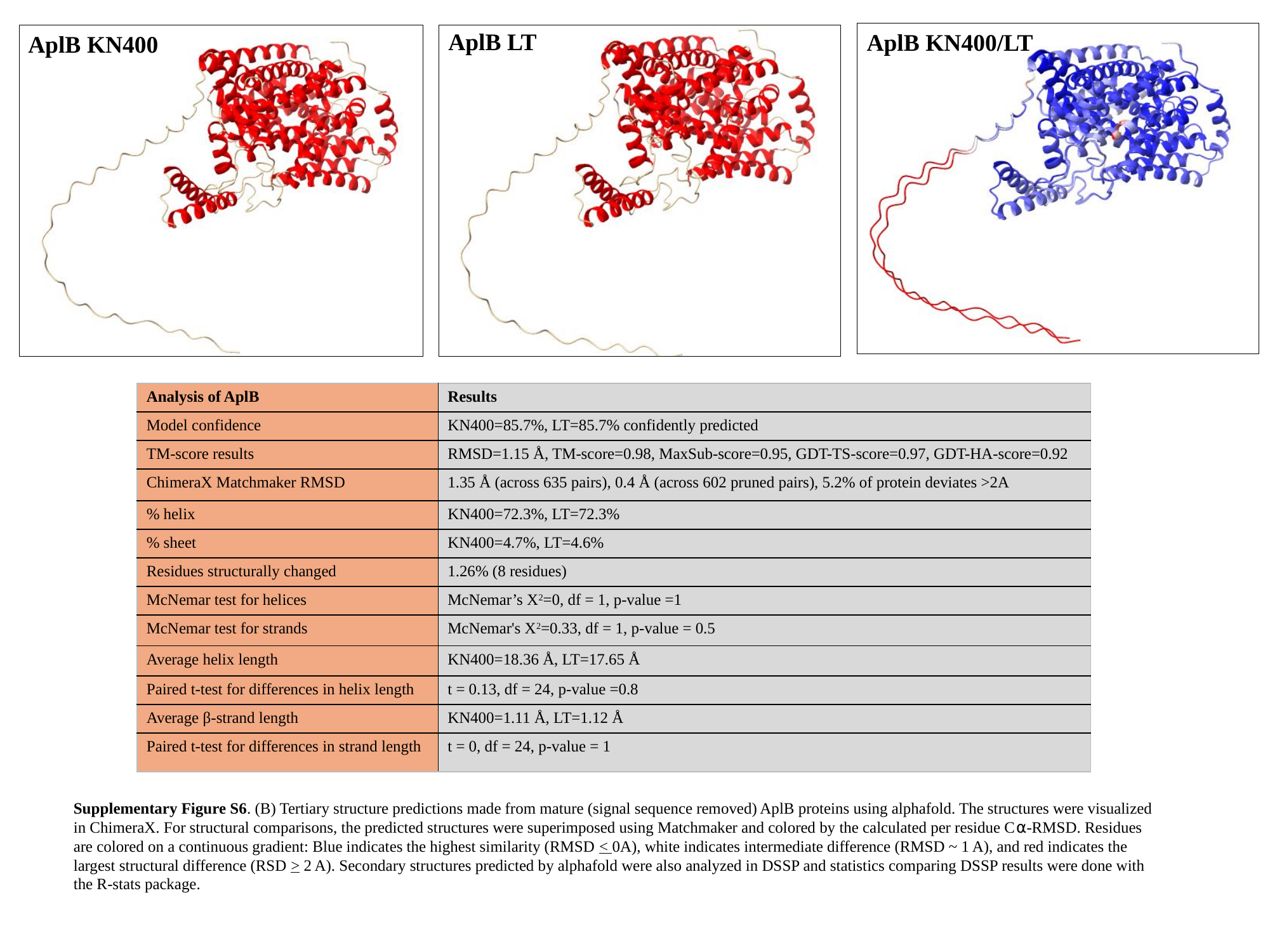

AplB LT
AplB KN400/LT
AplB KN400
| Analysis of AplB | Results |
| --- | --- |
| Model confidence | KN400=85.7%, LT=85.7% confidently predicted |
| TM-score results | RMSD=1.15 Å, TM-score=0.98, MaxSub-score=0.95, GDT-TS-score=0.97, GDT-HA-score=0.92 |
| ChimeraX Matchmaker RMSD | 1.35 Å (across 635 pairs), 0.4 Å (across 602 pruned pairs), 5.2% of protein deviates >2A |
| % helix | KN400=72.3%, LT=72.3% |
| % sheet | KN400=4.7%, LT=4.6% |
| Residues structurally changed | 1.26% (8 residues) |
| McNemar test for helices | McNemar’s X2=0, df = 1, p-value =1 |
| McNemar test for strands | McNemar's X2=0.33, df = 1, p-value = 0.5 |
| Average helix length | KN400=18.36 Å, LT=17.65 Å |
| Paired t-test for differences in helix length | t = 0.13, df = 24, p-value =0.8 |
| Average β-strand length | KN400=1.11 Å, LT=1.12 Å |
| Paired t-test for differences in strand length | t = 0, df = 24, p-value = 1 |
Supplementary Figure S6. (B) Tertiary structure predictions made from mature (signal sequence removed) AplB proteins using alphafold. The structures were visualized in ChimeraX. For structural comparisons, the predicted structures were superimposed using Matchmaker and colored by the calculated per residue Cα-RMSD. Residues are colored on a continuous gradient: Blue indicates the highest similarity (RMSD < 0A), white indicates intermediate difference (RMSD ~ 1 A), and red indicates the largest structural difference (RSD > 2 A). Secondary structures predicted by alphafold were also analyzed in DSSP and statistics comparing DSSP results were done with the R-stats package.

## Slide 3
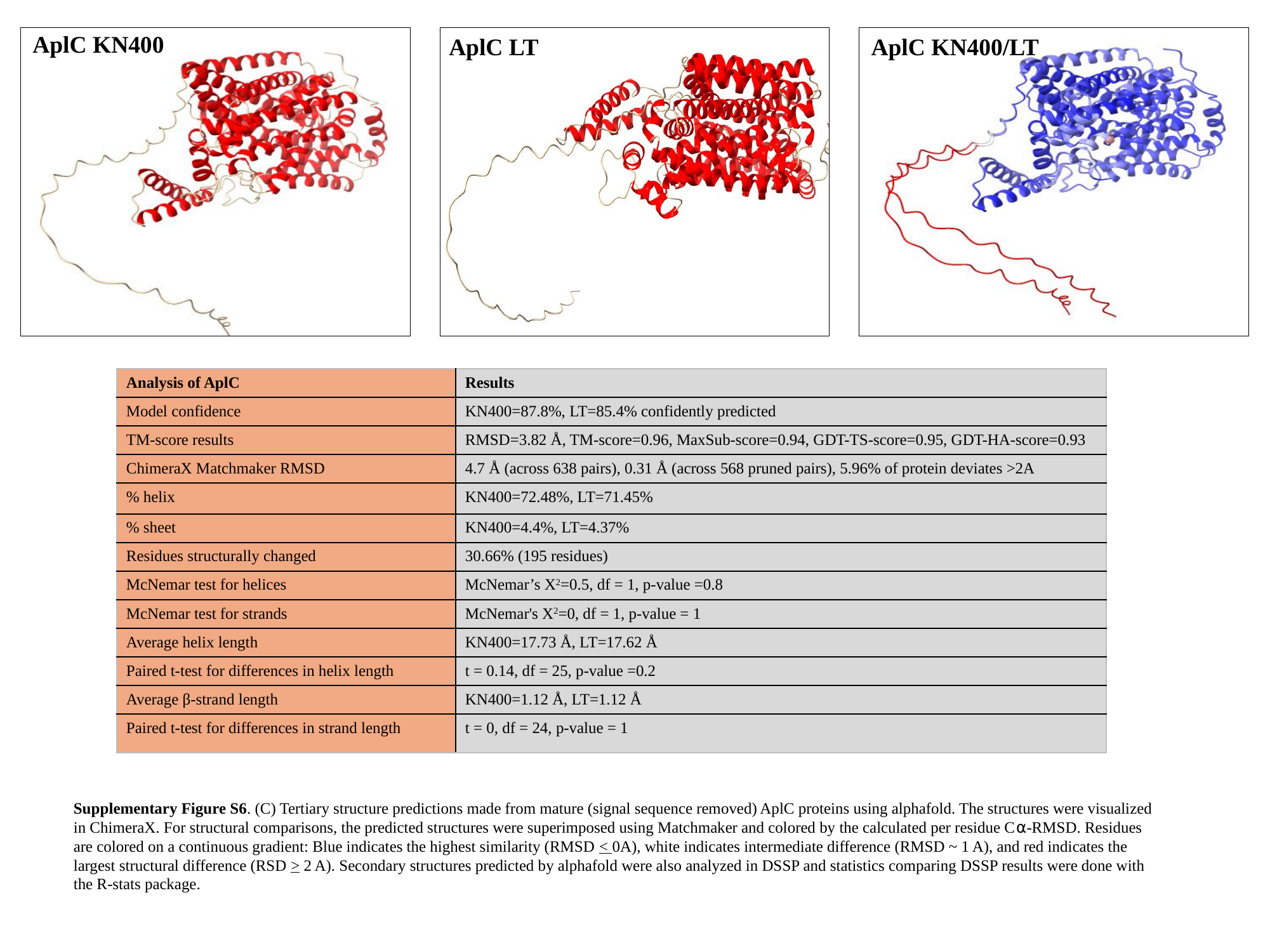

AplC KN400
AplC LT
AplC KN400/LT
| Analysis of AplC | Results |
| --- | --- |
| Model confidence | KN400=87.8%, LT=85.4% confidently predicted |
| TM-score results | RMSD=3.82 Å, TM-score=0.96, MaxSub-score=0.94, GDT-TS-score=0.95, GDT-HA-score=0.93 |
| ChimeraX Matchmaker RMSD | 4.7 Å (across 638 pairs), 0.31 Å (across 568 pruned pairs), 5.96% of protein deviates >2A |
| % helix | KN400=72.48%, LT=71.45% |
| % sheet | KN400=4.4%, LT=4.37% |
| Residues structurally changed | 30.66% (195 residues) |
| McNemar test for helices | McNemar’s X2=0.5, df = 1, p-value =0.8 |
| McNemar test for strands | McNemar's X2=0, df = 1, p-value = 1 |
| Average helix length | KN400=17.73 Å, LT=17.62 Å |
| Paired t-test for differences in helix length | t = 0.14, df = 25, p-value =0.2 |
| Average β-strand length | KN400=1.12 Å, LT=1.12 Å |
| Paired t-test for differences in strand length | t = 0, df = 24, p-value = 1 |
Supplementary Figure S6. (C) Tertiary structure predictions made from mature (signal sequence removed) AplC proteins using alphafold. The structures were visualized in ChimeraX. For structural comparisons, the predicted structures were superimposed using Matchmaker and colored by the calculated per residue Cα-RMSD. Residues are colored on a continuous gradient: Blue indicates the highest similarity (RMSD < 0A), white indicates intermediate difference (RMSD ~ 1 A), and red indicates the largest structural difference (RSD > 2 A). Secondary structures predicted by alphafold were also analyzed in DSSP and statistics comparing DSSP results were done with the R-stats package.

## Slide 4
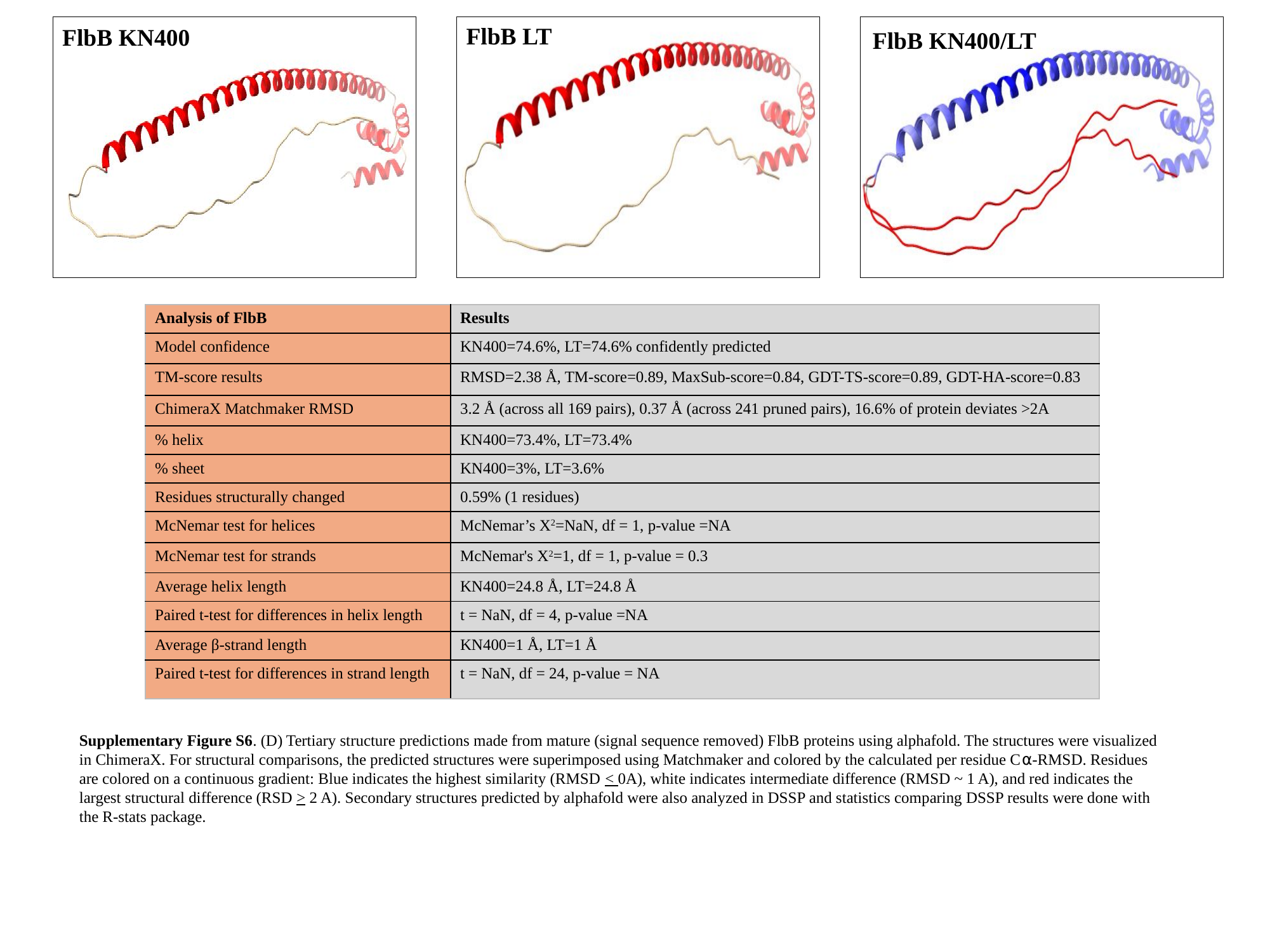

FlbB LT
FlbB KN400
FlbB KN400/LT
| Analysis of FlbB | Results |
| --- | --- |
| Model confidence | KN400=74.6%, LT=74.6% confidently predicted |
| TM-score results | RMSD=2.38 Å, TM-score=0.89, MaxSub-score=0.84, GDT-TS-score=0.89, GDT-HA-score=0.83 |
| ChimeraX Matchmaker RMSD | 3.2 Å (across all 169 pairs), 0.37 Å (across 241 pruned pairs), 16.6% of protein deviates >2A |
| % helix | KN400=73.4%, LT=73.4% |
| % sheet | KN400=3%, LT=3.6% |
| Residues structurally changed | 0.59% (1 residues) |
| McNemar test for helices | McNemar’s X2=NaN, df = 1, p-value =NA |
| McNemar test for strands | McNemar's X2=1, df = 1, p-value = 0.3 |
| Average helix length | KN400=24.8 Å, LT=24.8 Å |
| Paired t-test for differences in helix length | t = NaN, df = 4, p-value =NA |
| Average β-strand length | KN400=1 Å, LT=1 Å |
| Paired t-test for differences in strand length | t = NaN, df = 24, p-value = NA |
Supplementary Figure S6. (D) Tertiary structure predictions made from mature (signal sequence removed) FlbB proteins using alphafold. The structures were visualized in ChimeraX. For structural comparisons, the predicted structures were superimposed using Matchmaker and colored by the calculated per residue Cα-RMSD. Residues are colored on a continuous gradient: Blue indicates the highest similarity (RMSD < 0A), white indicates intermediate difference (RMSD ~ 1 A), and red indicates the largest structural difference (RSD > 2 A). Secondary structures predicted by alphafold were also analyzed in DSSP and statistics comparing DSSP results were done with the R-stats package.

## Slide 5
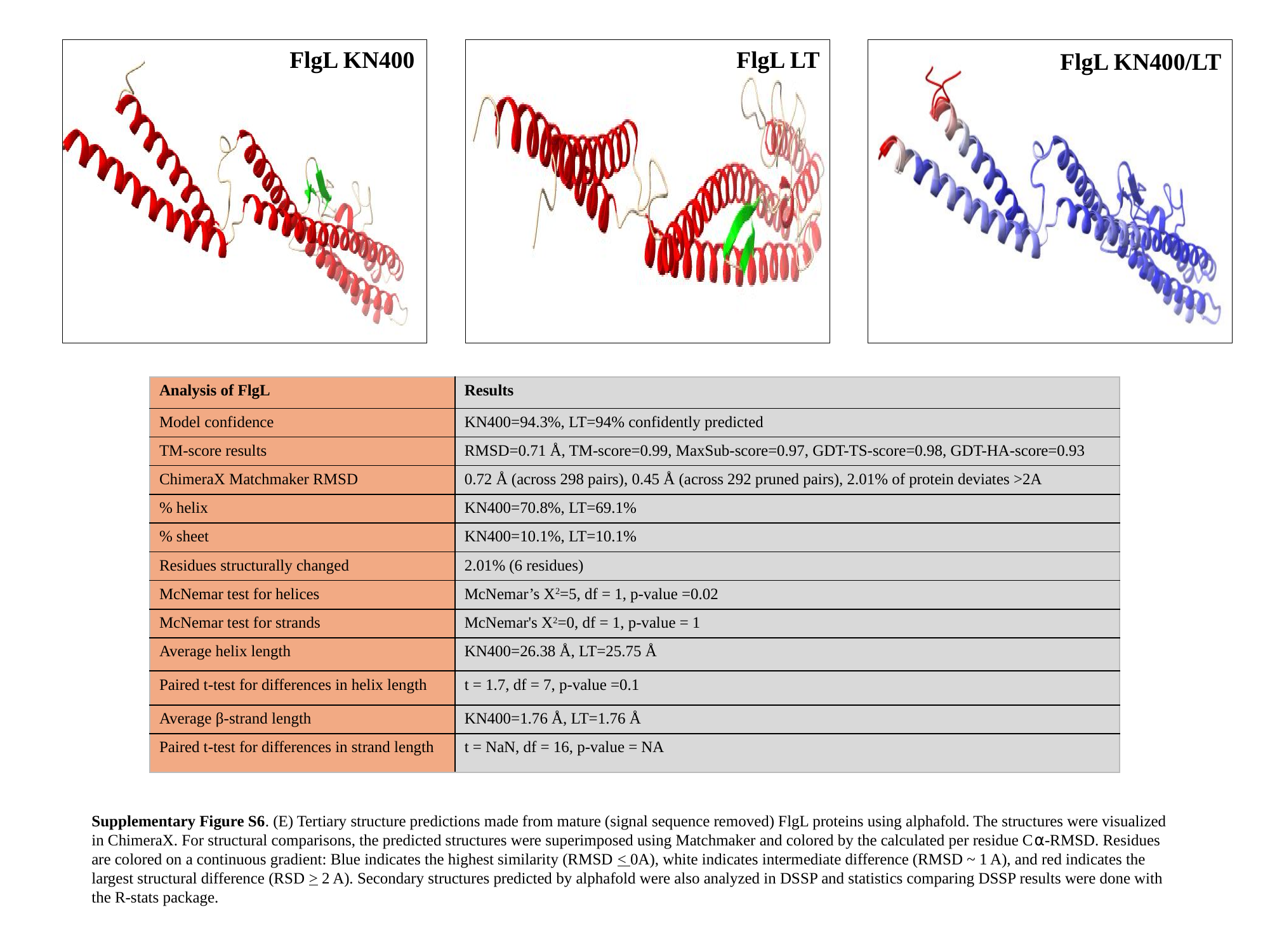

FlgL KN400
FlgL LT
FlgL KN400/LT
| Analysis of FlgL | Results |
| --- | --- |
| Model confidence | KN400=94.3%, LT=94% confidently predicted |
| TM-score results | RMSD=0.71 Å, TM-score=0.99, MaxSub-score=0.97, GDT-TS-score=0.98, GDT-HA-score=0.93 |
| ChimeraX Matchmaker RMSD | 0.72 Å (across 298 pairs), 0.45 Å (across 292 pruned pairs), 2.01% of protein deviates >2A |
| % helix | KN400=70.8%, LT=69.1% |
| % sheet | KN400=10.1%, LT=10.1% |
| Residues structurally changed | 2.01% (6 residues) |
| McNemar test for helices | McNemar’s X2=5, df = 1, p-value =0.02 |
| McNemar test for strands | McNemar's X2=0, df = 1, p-value = 1 |
| Average helix length | KN400=26.38 Å, LT=25.75 Å |
| Paired t-test for differences in helix length | t = 1.7, df = 7, p-value =0.1 |
| Average β-strand length | KN400=1.76 Å, LT=1.76 Å |
| Paired t-test for differences in strand length | t = NaN, df = 16, p-value = NA |
Supplementary Figure S6. (E) Tertiary structure predictions made from mature (signal sequence removed) FlgL proteins using alphafold. The structures were visualized in ChimeraX. For structural comparisons, the predicted structures were superimposed using Matchmaker and colored by the calculated per residue Cα-RMSD. Residues are colored on a continuous gradient: Blue indicates the highest similarity (RMSD < 0A), white indicates intermediate difference (RMSD ~ 1 A), and red indicates the largest structural difference (RSD > 2 A). Secondary structures predicted by alphafold were also analyzed in DSSP and statistics comparing DSSP results were done with the R-stats package.
