# Supplementary material for: Evolution for enhanced extracellular electron transfer in Geobacter sulfurreducens over seventeen years of continuous current generation
Source: Front Microbiol. 2026 May 8;17:1771963. doi: 10.3389/fmicb.2026.1771963 (PMC13194489; doi:10.3389/fmicb.2026.1771963)
Supplement: Supplementary file 1 [file Supplementary_file_1.zip › Supplementary Table 3.DOCX]

**Supplementary Table S3.** Tertiary structure predictions made from mature (signal sequence removed) OmcE proteins using alphafold. The structures were visualized and heme positions were labeled in ChimeraX. Heme localizations in the individual proteins are highlighted in purple. For structural comparisons, the predicted structures were superimposed using Matchmaker and colored by the calculated per residue Cα-RMSD measurement. Residues are colored on a continuous gradient: Blue indicates the highest similarity (RMSD < 0A), white indicates intermediate difference (RMSD ~ 1 A), and red indicates the largest structural difference (RSD > 2 A). Secondary structures predicted by alphafold were also analyzed in DSSP and statistics comparing DSSP results were done with the R-stats package.

| **Analysis of OmcE** | **Results** |
| --- | --- |
| Model confidence | PCA=97.6%, LT=95.7% confidently predicted |
| TM-score results | RMSD=0.8 Å, TM-score=0.97, MaxSub-score=0.95, GDT-TS-score=0.96, GDT-HA-score=0.83 |
| ChimeraX Matchmaker RMSD | 0.8 Å (across 209 pairs), 0.71 Å (across 202 pruned pairs), 3.35% of protein deviates >2Å |
| % helix | PCA=18.2%, LT=15.8% |
| % sheet | PCA=24.9%, LT=24.9% |
| Residues structurally changed | 9.6% (20 residues) |
| McNemar test for helices | McNemar’s X^2^=5, df = 1, p-value =0.02 |
| McNemar test for strands | McNemar's X^2^=0, df = 1, p-value = 1 |
| Average helix length | PCA=4.4 Å, LT=4 Å |
| Paired t-test for differences in helix length | t = 0.2, df = 7, p-value =0.8 |
| Average β-strand length | PCA=1.41 Å, LT=1.43 Å |
| Paired t-test for differences in strand length | t = -0.15, df = 36, p-value = 0.8 |
| Differences in heme distances | Mean difference = 0.1 Å; t = 1.74, df = 15, p-value = 0.05, 95% CI: -0.024 to 0.24 |
