# Supplementary material for: Evolution for enhanced extracellular electron transfer in Geobacter sulfurreducens over seventeen years of continuous current generation
Source: Front Microbiol. 2026 May 8;17:1771963. doi: 10.3389/fmicb.2026.1771963 (PMC13194489; doi:10.3389/fmicb.2026.1771963)
Supplement: Supplementary file 1 [file Supplementary_file_1.zip › Supplementary Table 1.PDF]

Supplementary Table S1. Primers used for qRT-PCR analysis.

Primer name	Sequence (5' to 3')
GS_pilA_8f	ATTACCCCCATACCCCAACA
GS_pilA_146r	GCAATTGCAGCGAGAATACC
GS_omcZ_404f	ACGGCAACTTCATCGACAAC
GS_omcZ_518r	ACAACATTGCCATCCGTAGC
GS_omcE_558f	CCTCCCCCTCTTCAAGTCAG
GS_omcE_696r	CTTCTTGTGGCAACCCAGAC
GS_omcB_1825f	TATGTGGCATCCCTTGAAGC
GS_omcB_1970r	TTGGTGAAGGCGTTAGAGGA
GS_recA_576f	CAACCAGATCCGGATGAAGA
GS_recA_679r	TGCGGATATCGAGACGTACC
GS_omcS_1097f	GCATGACCCGCTTCAACCTG
GS_omcS_1227r	GTAGGCAGCGGTCATCTCGT
GS_pgcA_164f	CGACTACCGCGGCAGTATCA
GS_pgcA_291r	TGTGAAGGAGCCGTCTGTGG
