# Supplementary material for: Evolution for enhanced extracellular electron transfer in Geobacter sulfurreducens over seventeen years of continuous current generation
Source: Front Microbiol. 2026 May 8;17:1771963. doi: 10.3389/fmicb.2026.1771963 (PMC13194489; doi:10.3389/fmicb.2026.1771963)
Supplement: Supplementary file 1 [file Supplementary_file_1.zip › Supplementary Figure 2.PPTX]

## Slide 1
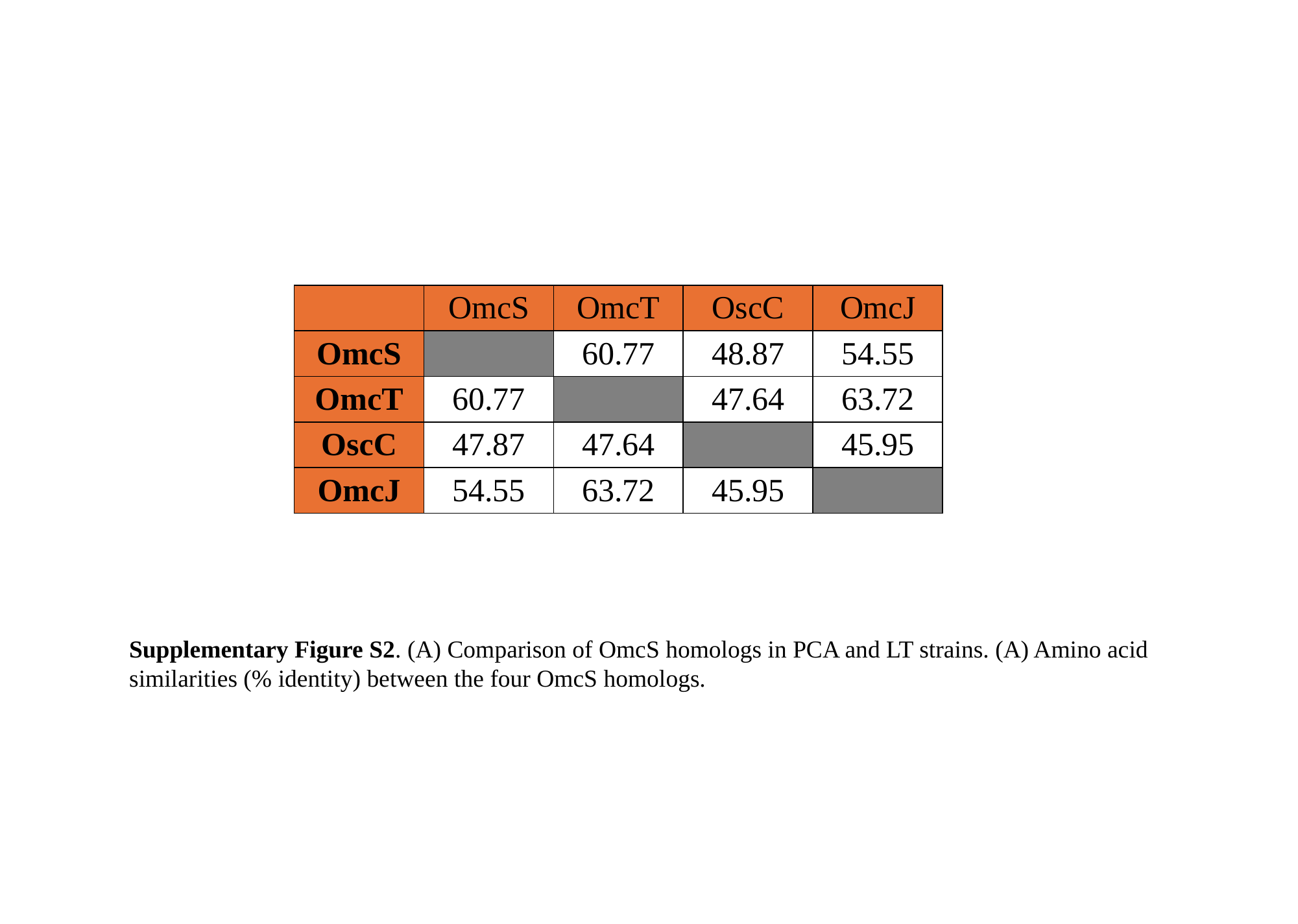

| | OmcS | OmcT | OscC | OmcJ |
| --- | --- | --- | --- | --- |
| OmcS | | 60.77 | 48.87 | 54.55 |
| OmcT | 60.77 | | 47.64 | 63.72 |
| OscC | 47.87 | 47.64 | | 45.95 |
| OmcJ | 54.55 | 63.72 | 45.95 | |
Supplementary Figure S2. (A) Comparison of OmcS homologs in PCA and LT strains. (A) Amino acid similarities (% identity) between the four OmcS homologs.

## Slide 2
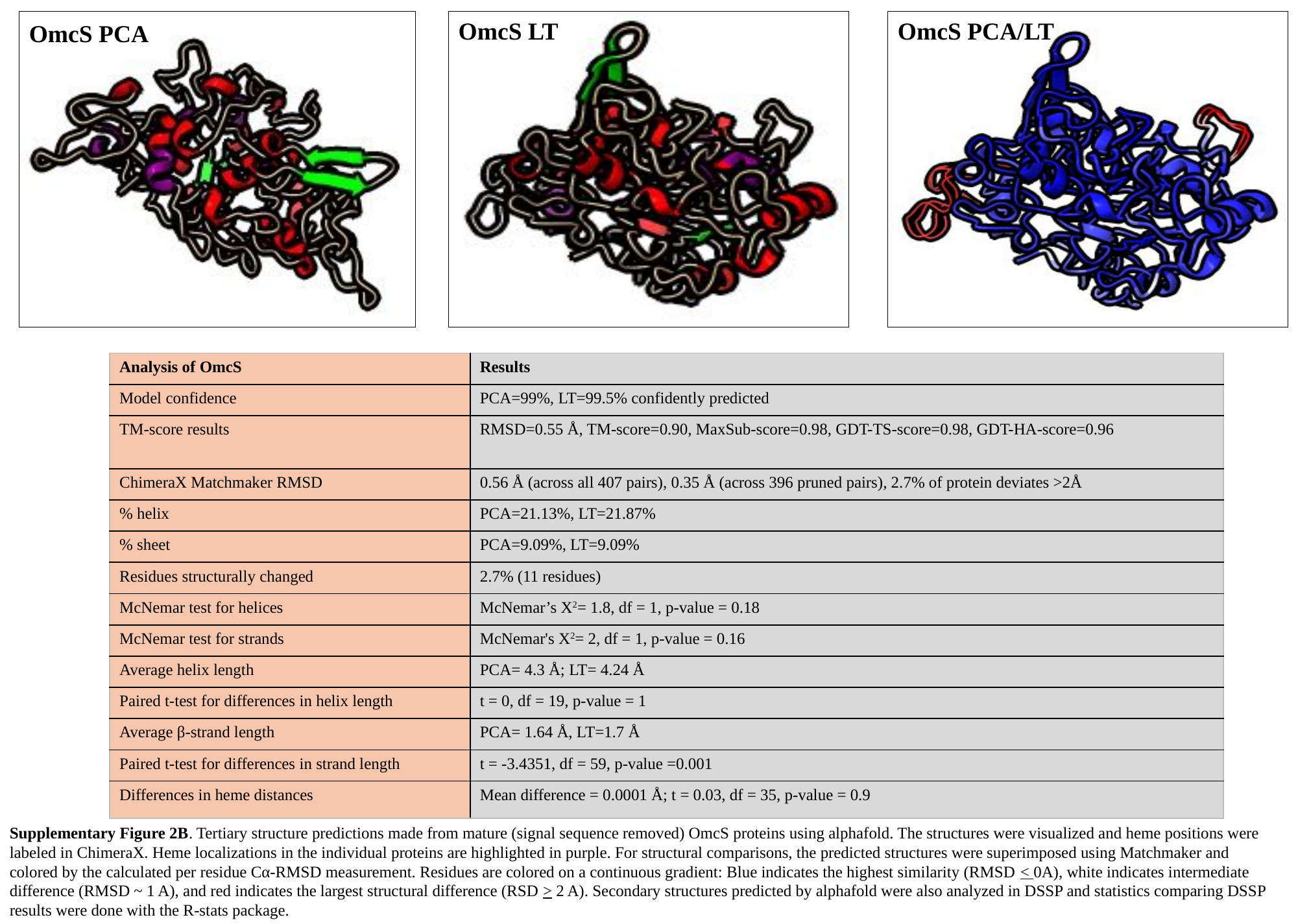

OmcS PCA/LT
OmcS LT
OmcS PCA
| Analysis of OmcS | Results |
| --- | --- |
| Model confidence | PCA=99%, LT=99.5% confidently predicted |
| TM-score results | RMSD=0.55 Å, TM-score=0.90, MaxSub-score=0.98, GDT-TS-score=0.98, GDT-HA-score=0.96 |
| ChimeraX Matchmaker RMSD | 0.56 Å (across all 407 pairs), 0.35 Å (across 396 pruned pairs), 2.7% of protein deviates >2Å |
| % helix | PCA=21.13%, LT=21.87% |
| % sheet | PCA=9.09%, LT=9.09% |
| Residues structurally changed | 2.7% (11 residues) |
| McNemar test for helices | McNemar’s X2= 1.8, df = 1, p-value = 0.18 |
| McNemar test for strands | McNemar's X2= 2, df = 1, p-value = 0.16 |
| Average helix length | PCA= 4.3 Å; LT= 4.24 Å |
| Paired t-test for differences in helix length | t = 0, df = 19, p-value = 1 |
| Average β-strand length | PCA= 1.64 Å, LT=1.7 Å |
| Paired t-test for differences in strand length | t = -3.4351, df = 59, p-value =0.001 |
| Differences in heme distances | Mean difference = 0.0001 Å; t = 0.03, df = 35, p-value = 0.9 |
Supplementary Figure 2B. Tertiary structure predictions made from mature (signal sequence removed) OmcS proteins using alphafold. The structures were visualized and heme positions were labeled in ChimeraX. Heme localizations in the individual proteins are highlighted in purple. For structural comparisons, the predicted structures were superimposed using Matchmaker and colored by the calculated per residue Cα-RMSD measurement. Residues are colored on a continuous gradient: Blue indicates the highest similarity (RMSD < 0A), white indicates intermediate difference (RMSD ~ 1 A), and red indicates the largest structural difference (RSD > 2 A). Secondary structures predicted by alphafold were also analyzed in DSSP and statistics comparing DSSP results were done with the R-stats package.

## Slide 3
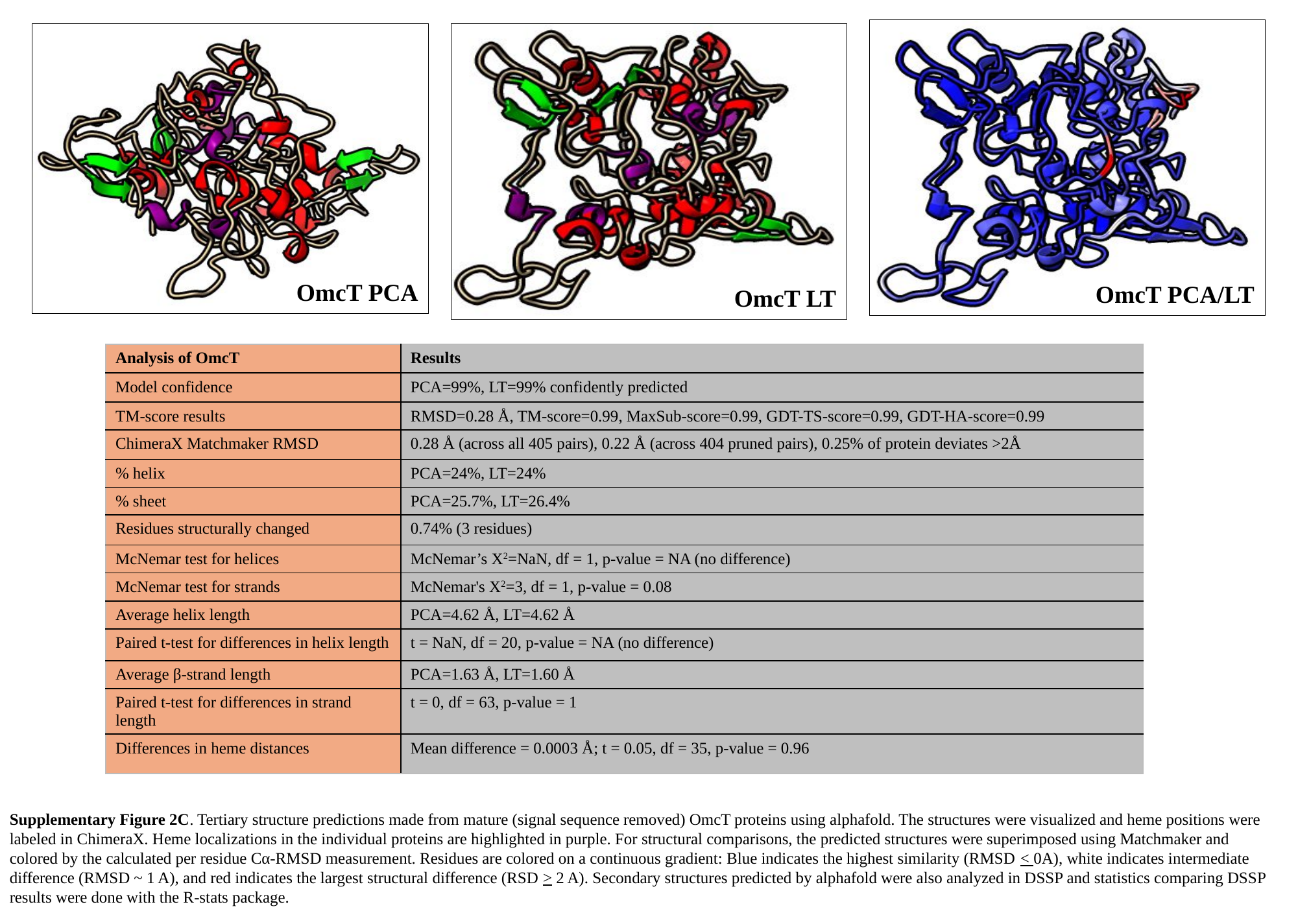

OmcT PCA
OmcT PCA/LT
OmcT LT
| Analysis of OmcT | Results |
| --- | --- |
| Model confidence | PCA=99%, LT=99% confidently predicted |
| TM-score results | RMSD=0.28 Å, TM-score=0.99, MaxSub-score=0.99, GDT-TS-score=0.99, GDT-HA-score=0.99 |
| ChimeraX Matchmaker RMSD | 0.28 Å (across all 405 pairs), 0.22 Å (across 404 pruned pairs), 0.25% of protein deviates >2Å |
| % helix | PCA=24%, LT=24% |
| % sheet | PCA=25.7%, LT=26.4% |
| Residues structurally changed | 0.74% (3 residues) |
| McNemar test for helices | McNemar’s X2=NaN, df = 1, p-value = NA (no difference) |
| McNemar test for strands | McNemar's X2=3, df = 1, p-value = 0.08 |
| Average helix length | PCA=4.62 Å, LT=4.62 Å |
| Paired t-test for differences in helix length | t = NaN, df = 20, p-value = NA (no difference) |
| Average β-strand length | PCA=1.63 Å, LT=1.60 Å |
| Paired t-test for differences in strand length | t = 0, df = 63, p-value = 1 |
| Differences in heme distances | Mean difference = 0.0003 Å; t = 0.05, df = 35, p-value = 0.96 |
Supplementary Figure 2C. Tertiary structure predictions made from mature (signal sequence removed) OmcT proteins using alphafold. The structures were visualized and heme positions were labeled in ChimeraX. Heme localizations in the individual proteins are highlighted in purple. For structural comparisons, the predicted structures were superimposed using Matchmaker and colored by the calculated per residue Cα-RMSD measurement. Residues are colored on a continuous gradient: Blue indicates the highest similarity (RMSD < 0A), white indicates intermediate difference (RMSD ~ 1 A), and red indicates the largest structural difference (RSD > 2 A). Secondary structures predicted by alphafold were also analyzed in DSSP and statistics comparing DSSP results were done with the R-stats package.

## Slide 4
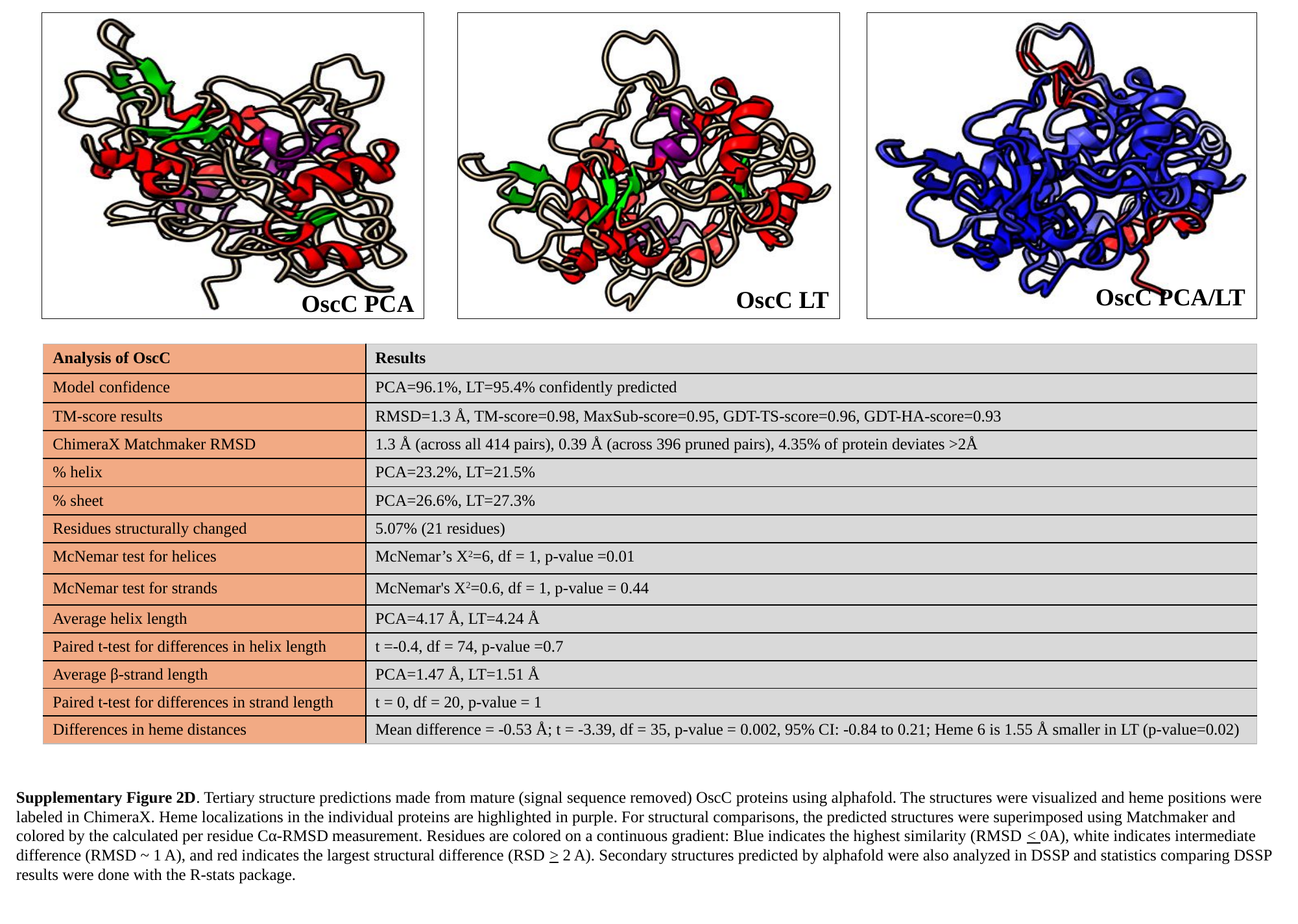

OscC PCA/LT
OscC LT
OscC PCA
| Analysis of OscC | Results |
| --- | --- |
| Model confidence | PCA=96.1%, LT=95.4% confidently predicted |
| TM-score results | RMSD=1.3 Å, TM-score=0.98, MaxSub-score=0.95, GDT-TS-score=0.96, GDT-HA-score=0.93 |
| ChimeraX Matchmaker RMSD | 1.3 Å (across all 414 pairs), 0.39 Å (across 396 pruned pairs), 4.35% of protein deviates >2Å |
| % helix | PCA=23.2%, LT=21.5% |
| % sheet | PCA=26.6%, LT=27.3% |
| Residues structurally changed | 5.07% (21 residues) |
| McNemar test for helices | McNemar’s X2=6, df = 1, p-value =0.01 |
| McNemar test for strands | McNemar's X2=0.6, df = 1, p-value = 0.44 |
| Average helix length | PCA=4.17 Å, LT=4.24 Å |
| Paired t-test for differences in helix length | t =-0.4, df = 74, p-value =0.7 |
| Average β-strand length | PCA=1.47 Å, LT=1.51 Å |
| Paired t-test for differences in strand length | t = 0, df = 20, p-value = 1 |
| Differences in heme distances | Mean difference = -0.53 Å; t = -3.39, df = 35, p-value = 0.002, 95% CI: -0.84 to 0.21; Heme 6 is 1.55 Å smaller in LT (p-value=0.02) |
Supplementary Figure 2D. Tertiary structure predictions made from mature (signal sequence removed) OscC proteins using alphafold. The structures were visualized and heme positions were labeled in ChimeraX. Heme localizations in the individual proteins are highlighted in purple. For structural comparisons, the predicted structures were superimposed using Matchmaker and colored by the calculated per residue Cα-RMSD measurement. Residues are colored on a continuous gradient: Blue indicates the highest similarity (RMSD < 0A), white indicates intermediate difference (RMSD ~ 1 A), and red indicates the largest structural difference (RSD > 2 A). Secondary structures predicted by alphafold were also analyzed in DSSP and statistics comparing DSSP results were done with the R-stats package.

## Slide 5
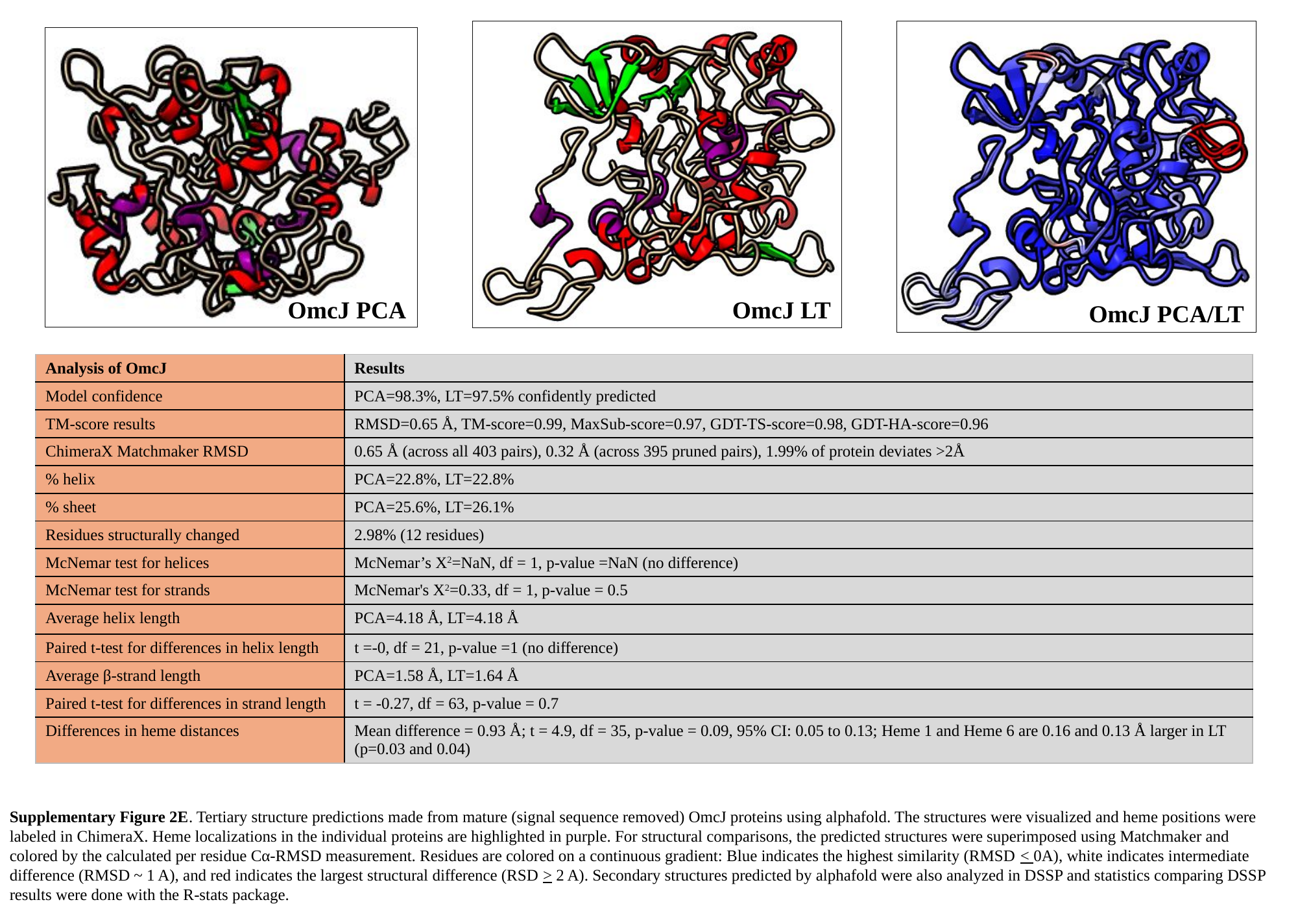

OmcJ LT
OmcJ PCA
OmcJ PCA/LT
| Analysis of OmcJ | Results |
| --- | --- |
| Model confidence | PCA=98.3%, LT=97.5% confidently predicted |
| TM-score results | RMSD=0.65 Å, TM-score=0.99, MaxSub-score=0.97, GDT-TS-score=0.98, GDT-HA-score=0.96 |
| ChimeraX Matchmaker RMSD | 0.65 Å (across all 403 pairs), 0.32 Å (across 395 pruned pairs), 1.99% of protein deviates >2Å |
| % helix | PCA=22.8%, LT=22.8% |
| % sheet | PCA=25.6%, LT=26.1% |
| Residues structurally changed | 2.98% (12 residues) |
| McNemar test for helices | McNemar’s X2=NaN, df = 1, p-value =NaN (no difference) |
| McNemar test for strands | McNemar's X2=0.33, df = 1, p-value = 0.5 |
| Average helix length | PCA=4.18 Å, LT=4.18 Å |
| Paired t-test for differences in helix length | t =-0, df = 21, p-value =1 (no difference) |
| Average β-strand length | PCA=1.58 Å, LT=1.64 Å |
| Paired t-test for differences in strand length | t = -0.27, df = 63, p-value = 0.7 |
| Differences in heme distances | Mean difference = 0.93 Å; t = 4.9, df = 35, p-value = 0.09, 95% CI: 0.05 to 0.13; Heme 1 and Heme 6 are 0.16 and 0.13 Å larger in LT (p=0.03 and 0.04) |
Supplementary Figure 2E. Tertiary structure predictions made from mature (signal sequence removed) OmcJ proteins using alphafold. The structures were visualized and heme positions were labeled in ChimeraX. Heme localizations in the individual proteins are highlighted in purple. For structural comparisons, the predicted structures were superimposed using Matchmaker and colored by the calculated per residue Cα-RMSD measurement. Residues are colored on a continuous gradient: Blue indicates the highest similarity (RMSD < 0A), white indicates intermediate difference (RMSD ~ 1 A), and red indicates the largest structural difference (RSD > 2 A). Secondary structures predicted by alphafold were also analyzed in DSSP and statistics comparing DSSP results were done with the R-stats package.
